# *Talaromyces marneffei* suppresses macrophage inflammation by regulating host alternative splicing

**DOI:** 10.1038/s42003-023-05409-6

**Published:** 2023-10-16

**Authors:** Wudi Wei, Gang Wang, Hong Zhang, Xiuli Bao, Sanqi An, Qiang Luo, Jinhao He, Lixiang Chen, Chuanyi Ning, Jingzhen Lai, Zongxiang Yuan, Rongfeng Chen, Junjun Jiang, Li Ye, Hao Liang

**Affiliations:** 1 Guangxi Key Laboratory of AIDS Prevention and Treatment, School of Public Health, Guangxi Medical University, Nanning, 530021 Guangxi China; 2 Guangxi-ASEAN Collaborative Innovation Center for Major Disease Prevention and Treatment, Life Sciences Institute, Guangxi Medical University, Nanning, 530021 Guangxi China; 3 Nursing College, Guangxi Medical University, Nanning, 530021 Guangxi China; 4 Guangxi Biobank, Life Sciences Institute, Guangxi Medical University, Nanning, 530021 Guangxi China

**Keywords:** Fungal host response, Fungal immune evasion

## Abstract

*Talaromyces marneffei* (*T. marneffei*) immune escape is essential in the pathogenesis of talaromycosis. It is currently known that *T. marneffei* achieves immune escape through various strategies. However, the role of cellular alternative splicing (AS) in immune escape remains unclear. Here, we depict the AS landscape in macrophages upon *T. marneffei* infection via high-throughput RNA sequencing and detect a truncated protein of NCOR2 / SMRT, named NCOR2-013, which is significantly upregulated after *T. marneffei* infection. Mechanistic analysis indicates that NCOR2-013 forms a co-repression complex with TBL1XR1 / TBLR1 and HDAC3, thereby inhibiting JunB-mediated transcriptional activation of pro-inflammatory cytokines via the inhibition of histone acetylation. Furthermore, we identify TUT1 as the AS regulator that regulates NCOR2-013 production and promotes *T. marneffei* immune evasion. Collectively, these findings indicate that *T. marneffei* escapes macrophage killing through TUT1-mediated alternative splicing of NCOR2 / SMRT, providing insight into the molecular mechanisms of *T. marneffei* immune evasion and potential targets for talaromycosis therapy.

## Abstract (web summary)

The study explores immune evasion mechanisms of *T. marneffei* infection, highlighting the role of alternative splicing (AS). *T. marneffei* triggers the upregulation of NCOR2-013, inhibiting pro-inflammatory cytokine production via histone acetylation. TUT1 regulates NCOR2-013, contributing to immune evasion. This sheds light on *T. marneffei*’s immune evasion at the molecular level, offering potential talaromycosis therapy targets.

## Introduction

Talaromycosis (formerly penicilliosis) is caused by a thermodimorphic fungus *Talaromyces marneffei* (*T. marneffei*) and is one of the important causes of death among AIDS patients in epidemic areas. *T. marneffei*, the only known dimorphic species in the genus, exhibits filamentous growth at 25 °C and a yeast phase at 37 °C, but only the yeast-like form has pathogenic potential^1^. Talaromycosis is a regional high-incidence opportunistic infection that is endemic throughout southern China, southeast Asia, and northeastern India^2,3^. Due to the HIV epidemic, the number of talaromycosis cases has rapidly increased and ranked 3rd as the most common HIV-associated opportunistic infections, accounting for up to 16% of HIV hospital admissions, and the leading cause of death among patients with advanced HIV in Thailand, Vietnam, and southern China^2–4^. By the end of 2018, 288,000 cases have been reported in 33 countries, with an estimated 17,300 cases (95% CI 9900–23,700) and 4900 deaths (95% CI 2500–7300) each year^5^. It is worth mentioning that with the wide application of bone marrow (organ) transplantation technology and immunosuppressive therapy, the incidence of HIV-negative talaromycosis patients is also increasing. Exploring differences in pathobiology between HIV-negative and positive talaromycosis is another important direction in the future research. As a result, talaromycosis has become a serious endemic health problem and has been proposed to be included in the “List of Priority Fungal Pathogens” by the World Health Organization, which is recognized as a neglected tropical infectious disease^6,7^.

Immune evasion of *T. marneffei* is an important reason for the poor prognosis of talaromycosis. Previous studies have shown that *T. marneffei* can survive in host macrophages. Ellett et al found that zebrafish embryo macrophages protect *T. marneffei* from neutrophil fungicidal activity during infection^8^. It is clear that *T. marneffei* has a set of macrophage-based immune evasion strategies in the humans. As a facultative intracellular fungus, *T. marneffei* is easily phagocytosed by resident macrophages after invasion^2,9^. Paradoxically, as one of the major cells in the innate immune line of defense against pathogen infection, macrophages also provide a niche for *T. marneffei* to evade immune killing, which is known as the “macrophage paradox”^10^. In fact, several pathogens have similar strategies, such as *Mycobacterium tuberculosis (M. tuberculosis)* and *Candida albicans* (*C. albicans*)^10–12^. Therefore, understanding the molecular mechanism of *T. marneffei* immune evasion is of great significance for the treatment of talaromycosis patients and the reduction of mortality. Previous studies have demonstrated that *T. marneffei* may escape macrophage killing by inducing M2-like macrophage polarization via regulating SOCS3-STAT6, TLR9, Jun1/2, and p38 signaling pathways, as well as LncRNAs^13–16^. However, there is still an urgent need for a deeper understanding of these mechanisms.

Alternative splicing (AS) is a ubiquitous mechanism for regulating gene expression, which allows a single gene to produce multiple unique mRNAs. It has been previously reported that 90–95% of human genes undergo multiple levels of AS, of which 37% have been shown to generate multiple protein isoforms that may play distinct biological functions^17–20^. In general, AS includes five categories of events, namely, retained intron (RI), exon skipping (ES), alternative 5’ splice site (A5SS), alternative 3’ splice site (A3SS), and mutually exclusive exon splicing (MXE), to generate multiple mature mRNA isoforms from the same pre-mRNA^21^. Thus, AS plays an important role in the complexity and functional diversity of the proteome. Bacterial pathogens are known to strategically modulate specific host factors via regulating AS to facilitate their replication in host cells^22–24^. For instance, *M. tuberculosis* can survive in macrophages by limiting the phagosome maturation by producing an abundance of truncated RAB8B variants^23^. As a highly dynamic and complex process, AS is regulated by a broad array of RNA-binding proteins (RBPs). Mechanistically, RBP binds to target pre-mRNA through specific motifs to mediate the occurrence of AS^17,19^. Intriguingly, global changes in gene AS events in macrophages after *T. marneffei* infection have not been understood, and there is no clear description of whether specific isoforms components contribute to *T. marneffei* immune evasion.

In this study, we performed high-throughput RNA sequencing and depicted a global view of the changes in gene AS events in human macrophages upon *T. marneffei* infection. Moreover, we identified a TUT1-modulated AS event of NCOR2-013 involved in facilitating *T. marneffei* immune evasion.

## Results

### Profiles of alternative splicing in *T. marneffei*-infected THP-1 macrophages

**Fig. 1:**
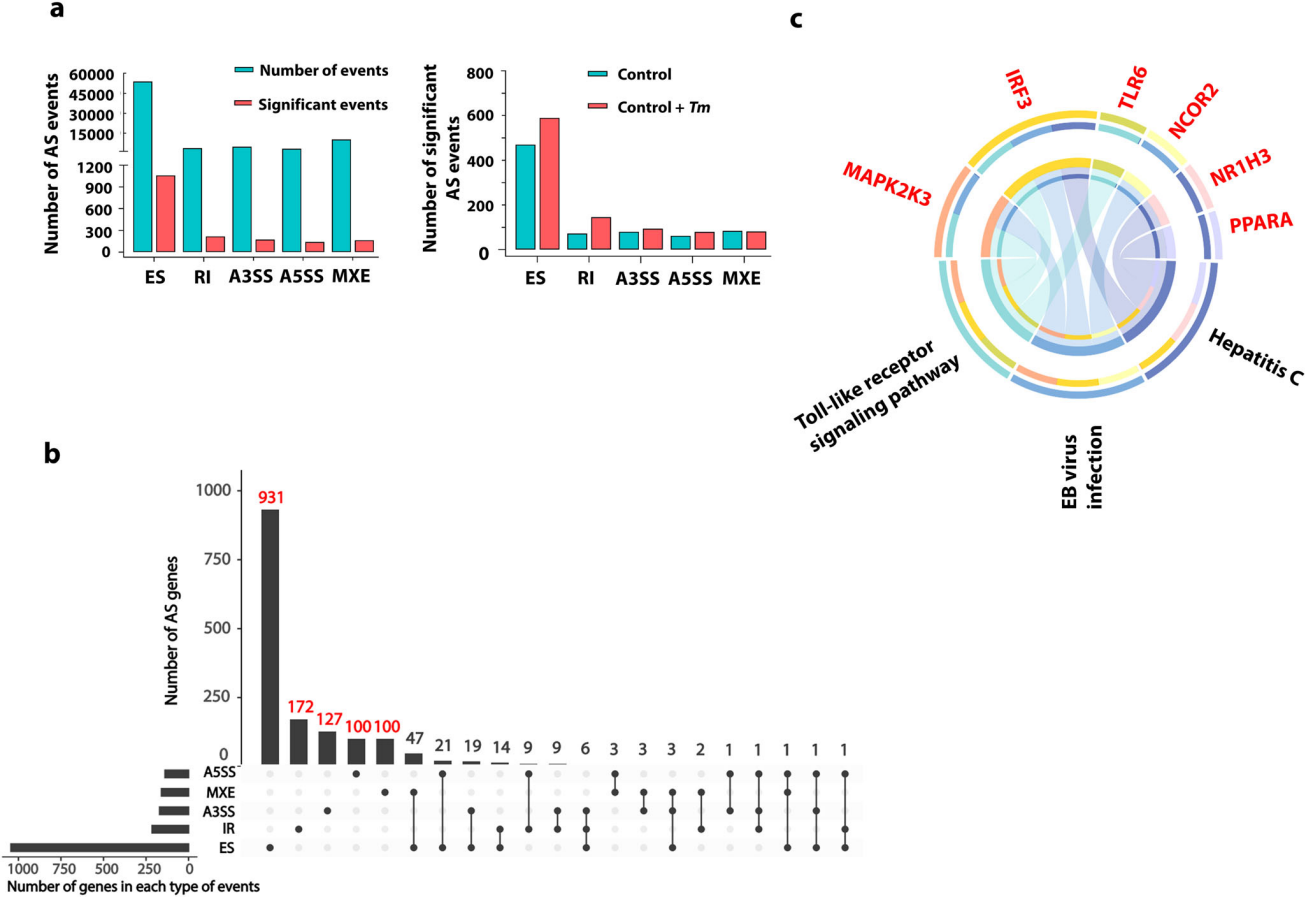
Landscape of alternative splicing (AS) in *T. marneffei*-infected THP-1 macrophages. **a** The numbers of different types of AS events in *T. marneffei*-infected and -uninfected THP-1 macrophages at 24 h post-infection. The left panel represents all AS events and significant differentially AS events (FDR < 0.05), the right panel represents the distribution of significant differentially AS events (FDR < 0.05) in macrophages with or without *T. marneffei* infection. **b** UpSet plot (an alternative Venn diagram) of different types of significant AS genes in *T. marneffei*-infected THP-1 macrophages at 24 h post-infection. The black bars on the left represent the numbers of each type of AS events. The black dots in the matrix at the right represent the intersections of AS events. The black dot-lines indicate the gene may have one or more alternative splicing patterns. **c** Chord diagram of the abundance of KEGG pathways according to ES-type AS genes with FDR < 0.05 and |Lnclevel| > 0.5 after infection of *T. marneffei* for 24 h. Supplementary material is available (Supplementary Fig. 1; Supplementary Data 1). AS, alternative splicing; ES exon skipping, RI retained intron, A5SS alternative 5’splice site, A3SS alternative 3’splice site, MXE mutually exclusive exon splicing, *Tm* *T. marneffei*.

To investigate the potential function of AS events involved in *T. marneffei*-infected macrophages, we performed RNA sequencing (RNA-seq) in *T. marneffei*-infected or -uninfected THP-1 macrophages at 24 h post-infection. Overall, 75,939 AS events were found in *T. marneffei*-infected macrophages, of which, 1757 were statistically significant. The proportion of ES in significant AS events was 60.33%, which was the highest compared with other types of AS events (Fig. 1a). The UpSet plot showed that 90.60% genes (1234/1362) were involved in only one type of AS event, among which ES type (763) was the largest group, followed by RI (166), A3SS (119), A5SS (95) and MXE (91), and the rest of genes were involved in 2 (8.96%, 122/1362) or 3 (0.44%, 6/1362) AS events simultaneously (Fig. 1b). Since ES type was the most common AS type in *T. marneffei*-infected macrophages, we next selected ES-type AS genes with FDR < 0.05 and |Lnclevel| > 0.5 for further KEGG enrichment analysis. Finally 6 genes (*NCOR2, NR1H3, PPARA, IRF3, MAP2K3*, and *TLR6*) were enriched in 3 immune-related pathways, including toll-like receptor signaling pathway, EB virus infection, and hepatitis C (Fig. 1c, Supplementary Fig. 1a). Therefore, we focused on these 6 genes for the further study.

### *T. marneffei* induces the expression of NCOR2-013 isoforms in macrophages

**Fig. 2:**
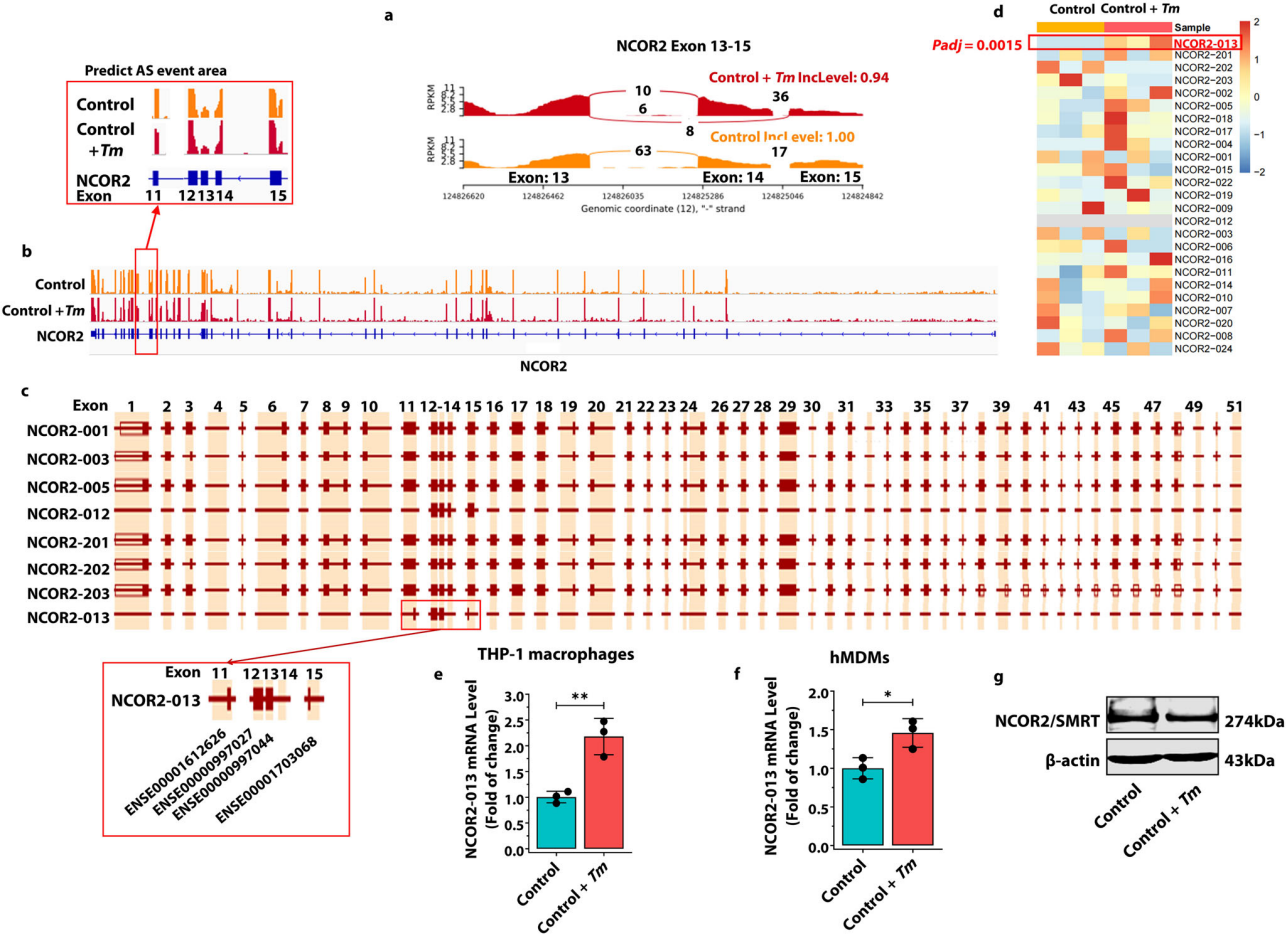
*T. marneffei* induces the expression of NCOR2-013 isoforms in macrophages. **a** Representative image of Sashimi plot depicting alternative splicing pattern of exons 13–15 of *NCOR2* in *T. marneffei*-infected or uninfected macrophages at 24 h post infection. The numbers of observed reads spanning the respective splice junctions are indicated on the Bezier curves connecting exons. **b** Genome tracks plots show the reads from *NCOR2* genes and the predicted AS areas by rMATS in *T. marneffei*-infected or -uninfected macrophages at 24 h post infection. Gene coding regions and annotations are shown in blue at the bottom. The exons and introns are represented by rectangles and straight lines, respectively. Exon 1 is on the left. The height of the peak represents the expression level of exons. The red box represents the site of AS predicted. **c** Ensembl genome browser tracks show the exon-intron organization of *NCOR2* transcripts. *NCOR2-013* exhibited the complete loss of exon 14 and partial loss of exons 11 and 15, suggesting an ES-type AS events. **d** The heatmap exhibits the expression levels of different *NCOR2* isoforms. The red box represents that the *NCOR2-13* isoforms was significantly up-regulated (*Padj* = 0.0015) at 24 h post infection (*n* = 3 biological replicates). RT-qPCR analysis of *NCOR2-013* isoforms expression in THP-1 macrophages (**e**) and hMDMs (**f**) at 24 h post infection. *GAPDH* was used as the loading control. The hMDMs were differentiated from PBMCs isolated from 3 healthy blood donors (*n* = 3 biological replicates, data are presented as mean values ± SD). **g** WB analysis of NCOR2 / SMRT expression in THP-1 macrophages at 24 h post infection. β-actin was used as the loading control. All data are shown as mean ± SD from three independent experiments (*n* = 3 biological replicates). WB were shown were the representative blot. Two-tailed Student’s *t* test was used to determine significance, denoted by * (*P* < 0.05), ** (*P* < 0.01), and ns (not significant). Supplementary material is available (Supplementary Figs. 1, 6; Supplementary Tables 1, 2; Supplementary Data 1). AS alternative splicing, *Tm* *T. marneffei*, hMDMs human monocyte-derived macrophages, PBMCs peripheral blood mononuclear cells.

Previous studies have shown that truncated transcripts produced by ES-type AS events play an important role in pathogen immunity^22–24^. Therefore, we aimed to identify specific truncated transcripts in the 6 ES-related AS genes (*NCOR2, NR1H3, PPARA, IRF3, MAP2K3*, and *TLR6*) that may influence the cellular responses to *T. marneffei* infection. We developed a strategy for the screening of particular truncated transcript. Firstly, the truncated transcripts generated by ES-type AS events predicted by rMATS should match the currently known transcripts. Secondly, the truncated transcript is differentially highly expressed in *T. marneffei*-infected macrophages, but not detected in uninfected cells. Due to the expression in uninfected cells or / and insignificant difference in transcript expression between *T. marneffei*-infected and -uninfected macrophages, 5 genes were excluded (Supplementary Table 1, Supplementary Fig. 1b–k) and only *NCOR2-013* was selected for in-depth study. Considerable differences were observed in the number of reads corresponding to exon-exon junctions between the *T. marneffei-*infected and -uninfected macrophages, as shown in Sashimi plots and track plots (Fig. 2a, b). Among different *NCOR2* isoforms, *NCOR2-013* exhibited the complete loss of exon 14 and partial loss of exons 11 and 15 (Fig. 2c). Subsequently, RNA-seq results revealed that *NCOR2-13* isoforms were not detected in control cells and significantly up-regulated *T. marneffei-*infected cells at 24 h post-infection (*padj* = 0.0015) (Fig. 2d, Supplementary Table 2). The RT-qPCR results confirmed that the *NCOR2-013* isoforms increased 2-fold at the mRNA level in *T. marneffei*-infected THP-1 macrophages at 24 h post-infection (Fig. 2e). In addition, the same trend was exhibited in *T. marneffei*-infected human monocyte-derived macrophages (hMDMs), with an approximately 1.5-fold increase (Fig. 2f). Interestingly, the maternal protein of NCOR2, namely SMRT, was not altered after *T. marneffei* infection, indicating that the biologically functional protein may not be the NCOR2 / SMRT, but more likely NCOR2-013 isoforms (Fig. 2g). Therefore, we focused on the functional roles and related mechanisms of NCOR2-013 in *T. marneffei*-infected macrophages.

### NCOR2-013 inhibits macrophage inflammatory response and antifungal activity

**Fig. 3:**
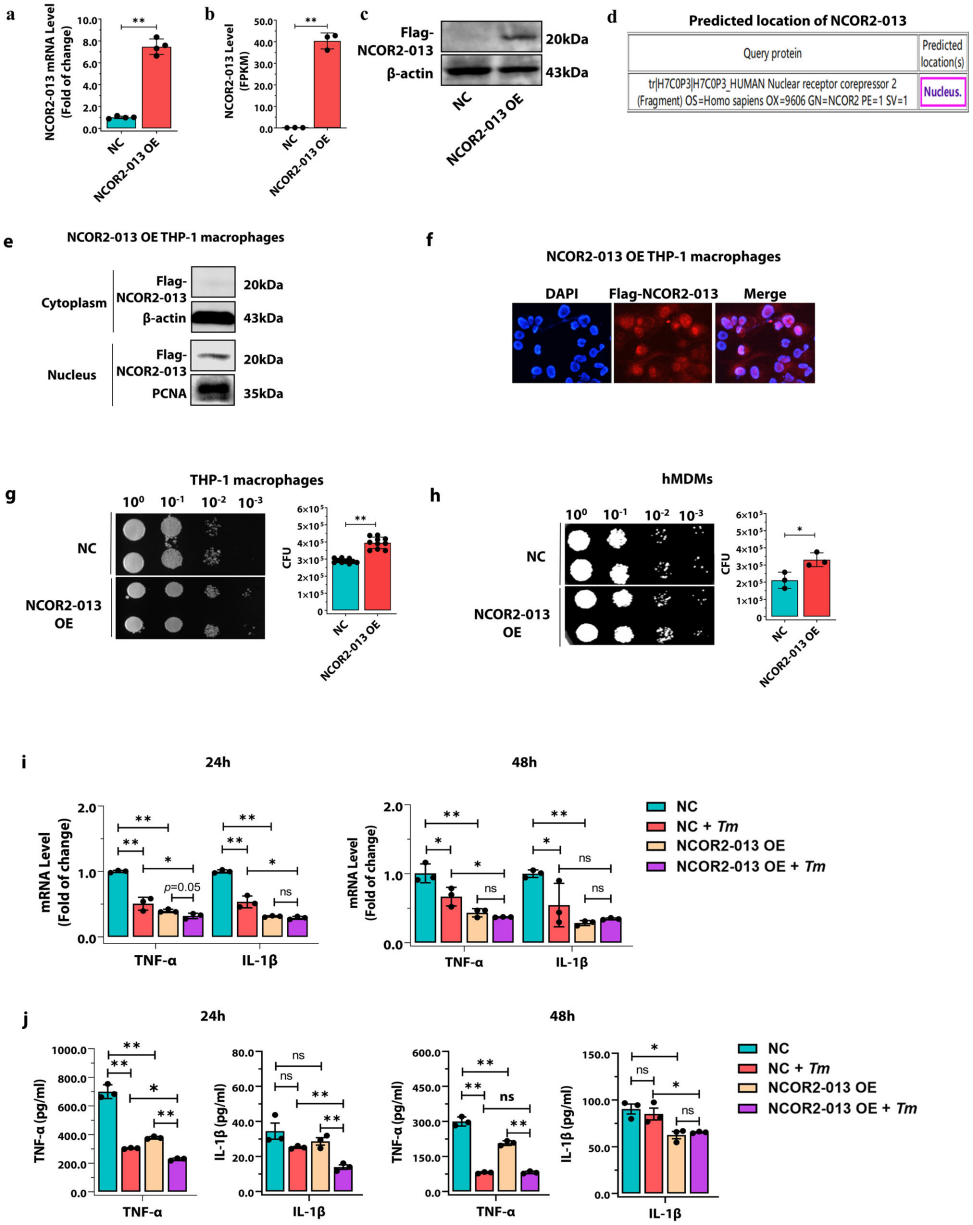
NCOR2-013 inhibits macrophage inflammatory response and antifungal activity. The human NCOR2-013 overexpressing THP-1 macrophages or NCOR2-013 overexpressing hMDMs were infected with *T. marneffei* conidia (MOI = 10) for 24 h. (a-c) NCOR2-013 overexpression in THP-1 macrophages was confirmed by RT-qPCR (**a**) (*n* = 4 biological replicates, data are presented as mean values ± SD), RNA-seq analysis (**b**) (*n* = 3 biological replicates, data are presented as mean values ± SD), and WB (**c**) (*n* = 3 biological replicates). **d** Prediction of subcellular localization for NCOR2-013 using the Hum-mPLoc 2.0 (http://www.csbio.sjtu.edu.cn/bioinf/hum-multi-2/). **e** The expression levels of NCOR2-013 in the cytoplasm and nucleus in NCOR2-013 overexpressing THP-1 macrophages were detected by WB. β-actin and PCNA were used as the loading control for cytoplasm and nucleus, respectively. **f** The subcellular localization and quantification of NCOR2-013 in NCOR2-013 overexpressing THP-1 macrophages were demonstrated by immunofluorescence. Quantification of the data using software ImageJ (*n* = 3 biological replicates). *T. marneffei* colony forming units (CFU) in NCOR2-013 overexpressing THP-1 macrophages (**g**) (*n* = 10 biological replicates, data are presented as mean values ± SD) or hMDMs (**h**) *n* = 3 biological replicates, data are presented as mean values ± SD, respectively. Four gradient serial dilutions (10^0^, 10^−1^, 10^−2^, 10^−3^) were performed. **i** The mRNA expression levels of *TNF-α* and *IL-1β* were detected by RT-qPCR in NCOR2-013 overexpressing THP-1 macrophages and control cells infected or uninfected with *T. marneffei* for 24 h and 48 h. *GAPDH* was used as the loading control (*n* = 3 biological replicates, data are presented as mean values ± SD). **j** The protein levels of TNF-α and IL-1β were measured using CBA human inflammatory cytokines kit in NCOR2-013 overexpressing THP-1 macrophages and control cells infected or uninfected with *T. marneffei* at 24 h and 48 h (*n* = 3 biological replicates, data are presented as mean values ± SD). WB were shown were the representative blot. Two-tailed Student’s *t* test was used to determine significance, denoted by * (*P* < 0.05), ** (*P* < 0.01), and ns (not significant). Supplementary material is available (Supplementary Fig. 6; Supplementary Data 1). hMDMs human monocyte-derived macrophages, MOI multiplicity of infection, NC negative control, NCOR2-013 OE NCRO2-013 overexpression, *Tm* *T. marneffei*.

To determine the subcellular localization and function of NCOR2-013, we successfully constructed a Flag-NCOR2-013 overexpressing THP-1 macrophage cell line using lentiviruses vectors and validated the cell line by RT-qPCR, RNA-seq and WB (Fig. 3a–c). We used the Hum-mPLoc 2.0 (http://www.csbio.sjtu.edu.cn/bioinf/hum-multi-2/) to predict subcellular localization of NCOR2-013, indicating that the protein is mainly located in the nucleus (Fig. 3d). Furthermore, we extracted cytoplasm and nucleus proteins of Flag-NCOR2-013 overexpressing macrophages, and found that Flag-NCOR2-013 was mainly localized in the nucleus and almost undetectable in the cytoplasm (Fig. 3e), which was also confirmed by immunofluorescence assay (Fig. 3f). CFUs assay revealed that more *T. marneffei* were detected in NCOR2-013 overexpressing macrophages compared with *T. marneffei*-infected control cells (Fig. 3g), suggesting that NCOR2-013 inhibits the ability of macrophages to clear *T. marneffei*. Similar results of CFUs assay were also observed in hMDMs (Fig. 3h). We further explored the effect of NCOR2-013 overexpression on the inflammatory response of macrophages, and found that *T. marneffei* infection and NCOR2-013 overexpression down-regulated the levels of *TNF-α* and *IL-1β* (NC vs. NC + *T. marneffei*, NC vs. NCOR2-013 overexpression). Meanwhile, with the premise of *T. marneffei* infection, the overexpression of NCOR2-013 can further suppress the expression of pro-inflammatory factors (NC + *T. marneffei* vs. NCOR2-013 overexpression+*T. marneffei*) (Fig. 3i, j).

### NCOR2-013 suppresses activation of JunB by forming a transcriptional regulatory complex with HDAC3 and TBL1XR1 / TBLR1

To explore the molecular mechanism of NCOR2-013 inhibiting the inflammatory response, we performed RNA-seq analysis on *T. marneffei*-infected NCOR2-013 overexpressing macrophages and control cells at 24 h post-infection. KEGG enrichment analysis showed the DEGs were significantly involved in NF-kappa B signaling pathway and TNF signaling pathway (Supplementary Fig. 2a), which was consistent with the GSEA results (Supplementary Fig. 2b), indicating that NCOR2-013 may inhibit the activation of NF-kappa B and TNF signaling pathways.

**Fig. 4:**
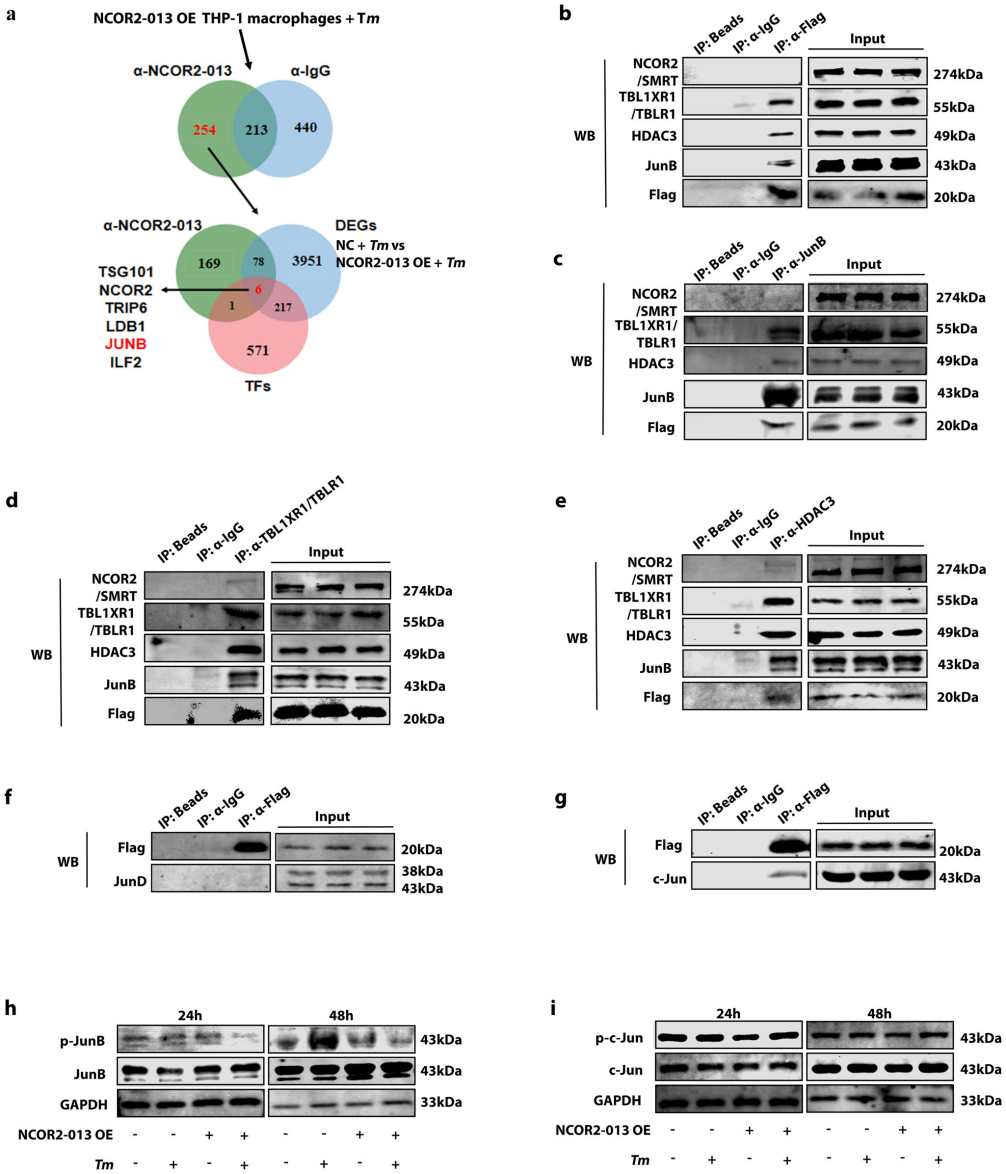
NCOR2-013 suppresses activation of JunB by forming a transcription-regulatory complex with HDAC3 and TBL1XR1/TBLR1. **a**–**g** The human NCOR2-013 overexpressing THP-1 macrophages were infected with *T. marneffei* conidia at MOI of 10 for 24 h. **a** The flowchart of potential binding protein screen for NCOR2-013. Briefly, anti-Flag antibody (for Flag-NCOR2-013) and isotype control IgG were used for immunoprecipitation of proteins, respectively, and LC-MS/MS was used to detect proteins that bound to NCOR2-013. The NCOR2-013 binding proteins were then intersected with DEGs (*T. marneffei*-infected NCRO2-013 overexpressing THP-1 macrophages vs. *T. marneffei*-infected control cells) and TFs (list of human transcription factors). **b** IP was performed with anti-Flag antibody (for Flag-NCOR2-013), and WB was used to detect NCOR2/SMRT, TBL1XR1/TBLR1, HDAC3 and JunB expression. IP assays were performed with JunB (**c**), TBL1XR1 / TBLR1 (**d**), and HDAC3 (**e**) antibody, respectively, to show interactions between NCOR2-013 and TBL1XR1/TBLR1, HDAC3, JunB, respectively. Co-IP assays show interactions between Flag-NCOR2-013 and JunD (**f**) or c-Jun (**g**). NCRO2-013 overexpressing THP-1 macrophages and control cells were infected with *T. marneffei* fo*r* 24 h and 48 h, p-JunB and JunB (**h**), p-c-Jun and c-Jun (**i**) were detected by WB. β-actin was used as the loading control. Experiment was performed thrice (*n* = 3 biological replicates) and a representative blot is shown. Supplementary material is available (Supplementary Figs. 2, 6). MOI multiplicity of infection, DEGs differential expression genes, TFs transcription factors, NCOR2-013 OE NCRO2-013 overexpression, *Tm T. marneffei*.

Previous studies have shown that NCOR2 / SMRT is a nuclear receptor that forms a co-repressor with HDAC3 and TBL1XR1 / TBLR1 to inhibit transcription factor activation^25^. Since the NCOR2-013 is predominantly located in the nucleus, we hypothesized that the regulatory mechanism of NCOR2-013 is similar to that of NCOR2 / SMRT. Therefore, we first identified 8 differentially expressed transcription factors (TWIST1, RB1, STAT1, RELA, HDGF, PPARG, E2F1 and JUN)^26^ via TRRUST analysis combined with DEGs (Supplementary Fig. 2c, d). We then infected NCOR2-013 overexpressing THP-1 macrophages with *T. marneffei* for 24 h, and screened for proteins binding to NCOR2-013 using IP and mass spectrometry (Fig. 4a). A total of 254 proteins were screened, and after intersecting with DEGs (NC + *T. marneffei* vs. NCOR2-013 overexpression + *T. marneffei*), 6 transcription factors were obtained (Fig. 4a). Combined with the results in Supplementary Fig. 2d, JunB was finally selected as the most potential transcription factor for subsequent functional research. To test the hypothesis above, we first verified the interaction between NCOR2-013 (Flag-tagged), TBL1XR1 / TBLR1, HDAC3, and JunB via Co-IP. The results showed that the 4 proteins could bind together in *T. marneffei*-infected NCOR2-013 overexpressing macrophages at 24 h post-infection (Fig. 4b–e). We also investigated whether NCOR2 / SMRT was involved in inhibition of JunB and performed Co-IP to explore the interaction of NCOR2 / SMRT with these 4 proteins. Interestingly, Co-IP results showed that NCOR2 / SMRT interacts with HDAC3, TBL1XR1 / TBLR1 (Fig. 4b–e). However, it does not interact with NCOR2-013 and JunB (Fig. 4b–e). In addition, because JunD and c-Jun are both homologous proteins of JunB^27^, we further verified whether NCOR2-013 could bind to JunD and c-Jun via Co-IP. The results showed that, in addition to JunB, NCOR2-013 also binds to c-Jun but hardly interaction with JunD (Fig. 4f, g). Next, we detected the activation levels of JunB and c-Jun by WB, respectively. The results showed that, only at 48 h post infection, not at 24 h, *T. marneffei* up-regulated the phosphorylation level of JunB, while NCOR2-013 overexpression inhibited the elevation of p-JunB, but there was no significant difference in p-c-Jun under *T. marneffei* infection or NCOR2-013 overexpression (Fig. 4h, i). Collectively, NCOR2-013 forms the NCOR2-013 - HDAC3 - TBL1XR1 / TBLR1 transcription-regulating complex and inhibits JunB activation.

### NCOR2-013 inhibits transcription of pro-inflammatory cytokines by inhibiting acetylated histone H3

**Fig. 5:**
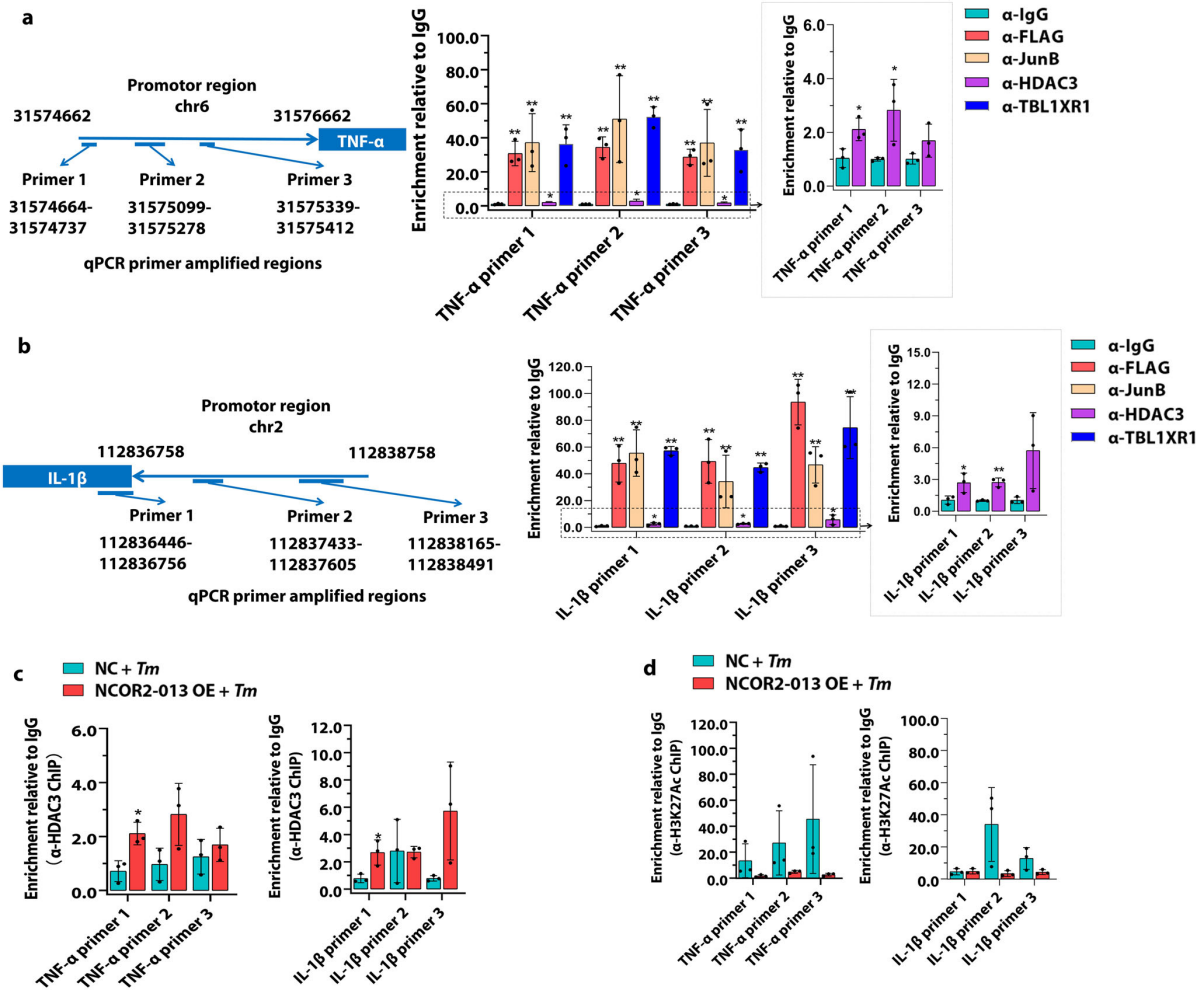
NCOR2-013 inhibits transcription of pro-inflammatory cytokines by inhibiting acetylated histone H3. The human NCOR2-013 overexpressing THP-1 macrophages were infected with *T. marneffei* conidia at MOI of 10 for 24 h. **a**, **b** ChIP analysis was performed using anti-Flag (for Flag-NCOR2-013) or HDAC3, TBL1XR1/TBLR1, and JunB antibody, respectively, to detect the enrichment levels of these proteins in the *TNF-α* and *IL-1β* promoter regions. The left panel represents three qPCR amplified regions for the promoter region of *TNF-α* (**a**) and *IL-1β* (**b**) (*n* = 3 biological replicates). The enrichment of HDAC3 (**c**) and histone H3K27Ac (**d**) in the *TNF-α* (left panel) and *IL-1β* (right panel) promoter regions were analyzed by ChIP-qPCR (*n* = 3 biological replicates). The results were computed as percent antibody bound per input and data were displayed after subtracting control IgG values. Two-tailed Student’s *t* test was used to determine significance, denoted by * (*P* < 0.05), ** (*P* < 0.01), and ns (not significant). Supplementary material is available (Supplementary Data 1). MOI multiplicity of infection, NC negative control, NCOR2-013 OE NCRO2-013 overexpression, *Tm* *T. marneffei*.

To explore whether NCOR2-013 transcription-regulating complex directly regulates transcription of pro-inflammatory cytokines, we performed ChIP-qPCR analysis using Flag, HDAC3, TBL1XR1/TBLR1 and JunB antibodies, respectively. The level of transcription factor occupancy of the promoter region of *TNF-α* and *IL-1β* in *T. marneffei*-infected NCOR2-013 overexpressing macrophages at 24 h post-infection were detected. The results showed that NCOR2-013, HDAC3, TBL1XR1 / TBLR1 and JunB were enriched in the *TNF-α* and *IL-1β* promoter regions, indicating that these proteins could act on the promoter regions of *TNF-α* and *IL-1β* genes (Fig. 5a, b).

In epigenetics, histone deacetylation via HDAC3 is a common mechanism associated with transcriptional activation by modulating chromatin condensation^28–30^. To further explore the molecular mechanism of NCOR2-013 suppressing the expression of pro-inflammatory cytokines, NCOR2-013-overexpressing macrophages and control cells were infected with *T. marneffei* for 24 h, and ChIP-qPCR assays were conducted using HDAC3 and H3K27Ac antibodies (Fig. 5c, d). The results showed that NOCR2-013 up-regulated the enrichment level of HDAC3 in promoter regions of *TNF-α* and *IL-1β*, and down-regulated the enrichment level of H3K27Ac, indicating that NOCR2-013 inhibited the histone H3 acetylation, which did not favor the binding of transcriptional factors to the corresponding promoter regions, thereby suppressing the expression of *TNF-α* and *IL-1β* (Fig. 5c, d).

### JunB overexpression or IFN-γ has little effect on NCOR2-013-mediated inhibition of pro-inflammatory response in macrophages, while LPS has partial effect

**Fig. 6:**
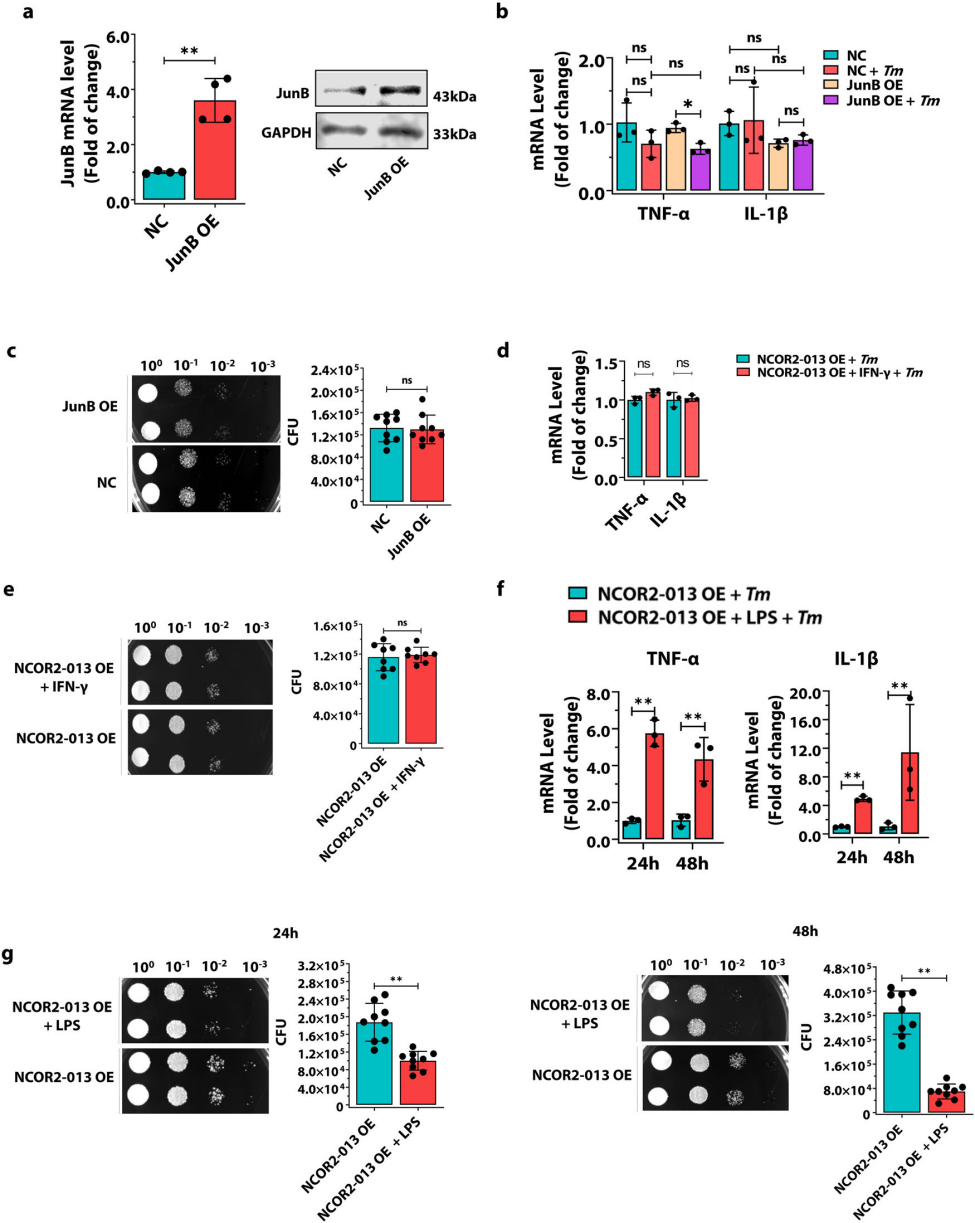
JunB overexpression or IFN-γ has little effect on NCOR2-013-mediated inhibition of inflammatory response in macrophages, while LPS has partial effect. **a** JunB overexpression in NCOR2-013 overexpressing THP-1 macrophages was confirmed by RT-qPCR and WB (*n* = 4 biological replicates, data are presented as mean values ± SD). **b**, **c** The JunB overexpressing THP-1 macrophages and control cells were infected with *T. marneffei* conidia (MOI = 10) for 24 h. The expression of *TNF-α* and *IL-1β* was detected by RT-qPCR (**b**) (*n* = 3 biological replicates, data are presented as mean values ± SD). *T. marneffei* CFUs were detected in JunB overexpressing THP-1 macrophages and control cells (**c**). Four gradient serial dilutions (10^0^, 10^−1^, 10^−2^, 10^−3^) were performed (*n* = 9 biological replicates, data are presented as mean values ± SD). **d**, **e** The NCOR2-013 overexpressing THP-1 macrophages and control cells were stimulated with IFN-γ (100 ng/mL) for 24 h, and then infected with *T. marneffei* conidia (MOI = 10) for 24 h. The expression of *TNF-α* and *IL-1β* was detected by RT-qPCR (**d**) (*n* = 3 biological replicates, data are presented as mean values ± SD). *T. marneffei* CFUs were detected in IFN-γ-stimulated NCOR2-013 overexpressing THP-1 macrophages and control cells (**e**). Four gradient serial dilutions (10^0^, 10^−1^, 10^−2^, 10^−3^) were performed (*n* = 8 biological replicates, data are presented as mean values ± SD). **f**, **g** The NCOR2-013 overexpressing THP-1 macrophages and control cells were stimulated with LPS (500 ng/mL) for 24 h, and then were infected with *T. marneffei* conidia (MOI = 10) for 24 h and 48 h. The expression of *TNF-α* and *IL-1β* was detected by RT-qPCR (**f**) (*n* = 3 biological replicates, data are presented as mean values ± SD). *T. marneffei* CFUs were detected in LPS-stimulated NCOR2-013 overexpressing THP-1 macrophages and control cells (**g**). Four gradient serial dilutions (10^0^, 10^−1^, 10^−2^, 10^−3^) were performed (*n* = 9 biological replicates, data are presented as mean values ± SD). All data are shown as mean ± SD from three independent experiments. WB were shown were the representative blot. Two-tailed Student’s *t* test was used to determine significance, denoted by *(*P* < 0.05), **(*P* < 0.01), and ns (not significant). Supplementary material is available (Supplementary Figs. 3, 6; Supplementary Data 1). MOI multiplicity of infection, NC negative control, NCOR2-013 OE NCRO2-013 overexpression, JunB OE JunB overexpression, *Tm T. marneffei*.

To explore the role of JunB in NCOR2-013-mediated inhibition of pro-inflammatory response, we overexpressed JunB in NCOR2-013-overexpressing THP-1 macrophages. However, JunB overexpression did not overcome the inhibitory effect of NCOR2-013, evidenced by the fact that JunB overexpression had no significant changes in cytokine expression and antifungal ability (Fig. 6a–c). Similarly, a parallel phenomenon was observed in NCOR2-013-untransfected cells (Supplementary Fig. 3). Subsequently, we conducted exploration experiments using IFN-γ and LPS, respectively. Interestingly, LPS, but not IFN-γ, was able to significantly upregulate the expression of *TNF-α* and *IL-1β* (Fig. 6d, e), while significantly increasing the killing ability of macrophages against *T. marneffei* (Fig. 6f, g), suggesting that distinct roles for IFN-γ and LPS in modulating the inflammatory and antifungal responses in NCOR2-013-overexpressing THP-1 macrophages, with LPS exerting a notably enhancing effect.

### TUT1-mediated generation of the *NCOR2-013* isoforms suppresses inflammatory response in *T. marneffei*-infected THP-1 macrophages

**Fig. 7:**
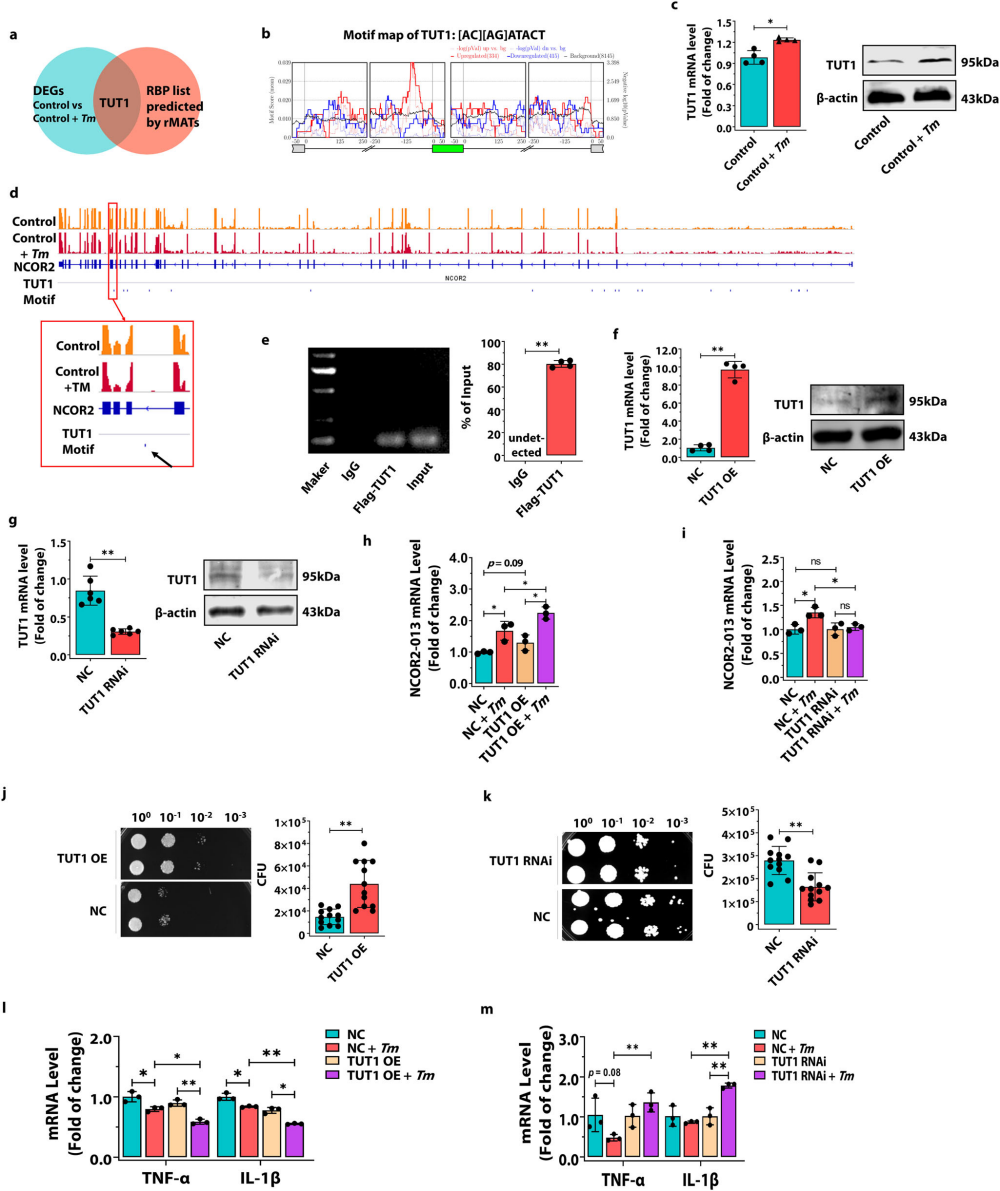
TUT1-mediated generation of the NCOR2-013 isoforms suppresses inflammatory response in *T. marneffei*-infected THP-1 macrophages. **a**–**d** The human THP-1 macrophages were infected with *T. marneffei* conidia (MOI = 10) for 24 h. **a** Identification of potential RBPs that regulating AS events. rMATS was used to predict potential RBPs and then intersected with DEGs (*T. marneffei*-infected THP-1 macrophages vs. control cells). **b** The TUT1 motif map shows the regional binding of TUT1 in the flanking introns of skipped and included exon events with change in PSI > 20%, FDR < 0.05. The red, blue, and black lines represent the motif scores for the upregulated, downregulated, and control (non-regulated background) exons, respectively. **c** RT-qPCR and WB to detect the expression levels of TUT1 in *T. marneffei*-infected or uninfected THP-1 macrophages (*n* = 4 biological replicates, data are presented as mean values ± SD). **d** Visualization of origin genome profiles was performed with IGV. The black arrow represents the position of TUT1 motif, suggesting that it may bind between exon 14 and 15 of NCOR2. **e** RIP analysis was performed to verify the interaction of TUT1 and *NCOR2* pre-mRNA. A Flag-tagged TUT1 was included as an indicator in the construction of TUT1 overexpressing macrophages. IP was performed with anti-Flag antibody, and RNA was subjected to agarose gel electrophoresis followed by RNA gel blot analysis (*n* = 4 biological replicates, data are presented as mean values ± SD). TUT1 overexpression (**f**) (*n* = 4 biological replicates, data are presented as mean values ± SD) and TUT1 knockdown (**g**) (*n* = 6 biological replicates, data are presented as mean values ± SD) in THP-1 macrophage were confirmed by RT-qPCR and WB. **h**–**m** THP-1 macrophages with TUT1 overexpression/knockdown and control cells were infected with *T. marneffei* (MOI = 10) for 24 h. The expression of *NCOR2-013* was detected by RT-qPCR in *T. marneffei*-infected or uninfected TUT1 overexpressing THP-1 macrophages (**h**) and TUT1 knockdown THP-1 macrophages (**i**) (*n* = 3 biological replicates, data are presented as mean values ± SD). *T. marneffei* CFUs in TUT1 overexpressing THP-1 macrophages (**j**) (*n* = 12 biological replicates, data are presented as mean values ± SD) and TUT1 knockdown THP-1 macrophages (**k**) (*n* = 12 biological replicates, data are presented as mean values ± SD). The levels of *TNF-α* and *IL-1β* were detected by RT-qPCR in. *T. marneffei*-infected or uninfected TUT1 overexpressing THP-1 macrophages (**l**) and TUT1 knockdown THP-1 macrophages (**m**). GAPDH was used as the loading control (*n* = 3 biological replicates, data are presented as mean values ± SD). WB were shown were the representative blot. Two-tailed Student’s *t* test was used to de*t*ermine significance, denoted by *(*P* < 0.05), **(*P* < 0.01), and ns (not significant). Supplementary material is available (Supplementary Fig. 6; Supplementary Data 1). MOI multiplicity of infection, NC negative control, TUT1 OE TUT1 overexpression, TUT1 RNAi TUT1 knockdown, *Tm T. marneffei*.

Generally, AS is mainly regulated by a series of RBPs. To identify the upstream RBPs that regulate *NCOR2* for AS, we used rMATS to predict splicing regulators, which were then intersected with DEGs derived from RNA-seq analysis, indicating that TUT1 is the likely RBP that regulates AS of *NCOR2* (Fig. 7a), with the motif of [AC][AG]ATACT (Fig. 7b). RT-qPCR and WB results confirmed that *T. marneffei* up-regulated the expression of TUT1 in THP-1 macrophages at 24 h post-infection (Fig. 7c). Subsequently, we performed the visualization of origin genome profiles via IGV, showing that the TUT1 motif can bind NCOR2 between exon 14 and 15 (Fig. 7d), suggesting that TUT1 may be involved in the AS event of exon 14 of *NCOR2* and mediate the generation of *NCOR2-013* isoforms (Fig. 2). To determine whether the effect on *NCOR2* AS was mediated by direct binding of TUT1, we performed RIP assay in *T. marneffei*-infected TUT1-overexpressing THP-1 macrophages at 24 h post-infection, and found that TUT1 binds to *NCOR2* pre-mRNA (Fig. 7e). Moreover, at 24 h post infection, the expression of *NCOR2-013* isoforms was significantly increased in the *T. marneffei*-infected TUT1-overexpressing THP-1 macrophages, but significantly decreased in the *T. marneffei*-infected TUT1-knockdown THP-1 macrophages (Fig. 7f–i). These results indicated that TUT1 plays an important role in the occurrence of AS in *NCOR2* upon *T. marneffei* infection.

**Fig. 8:**
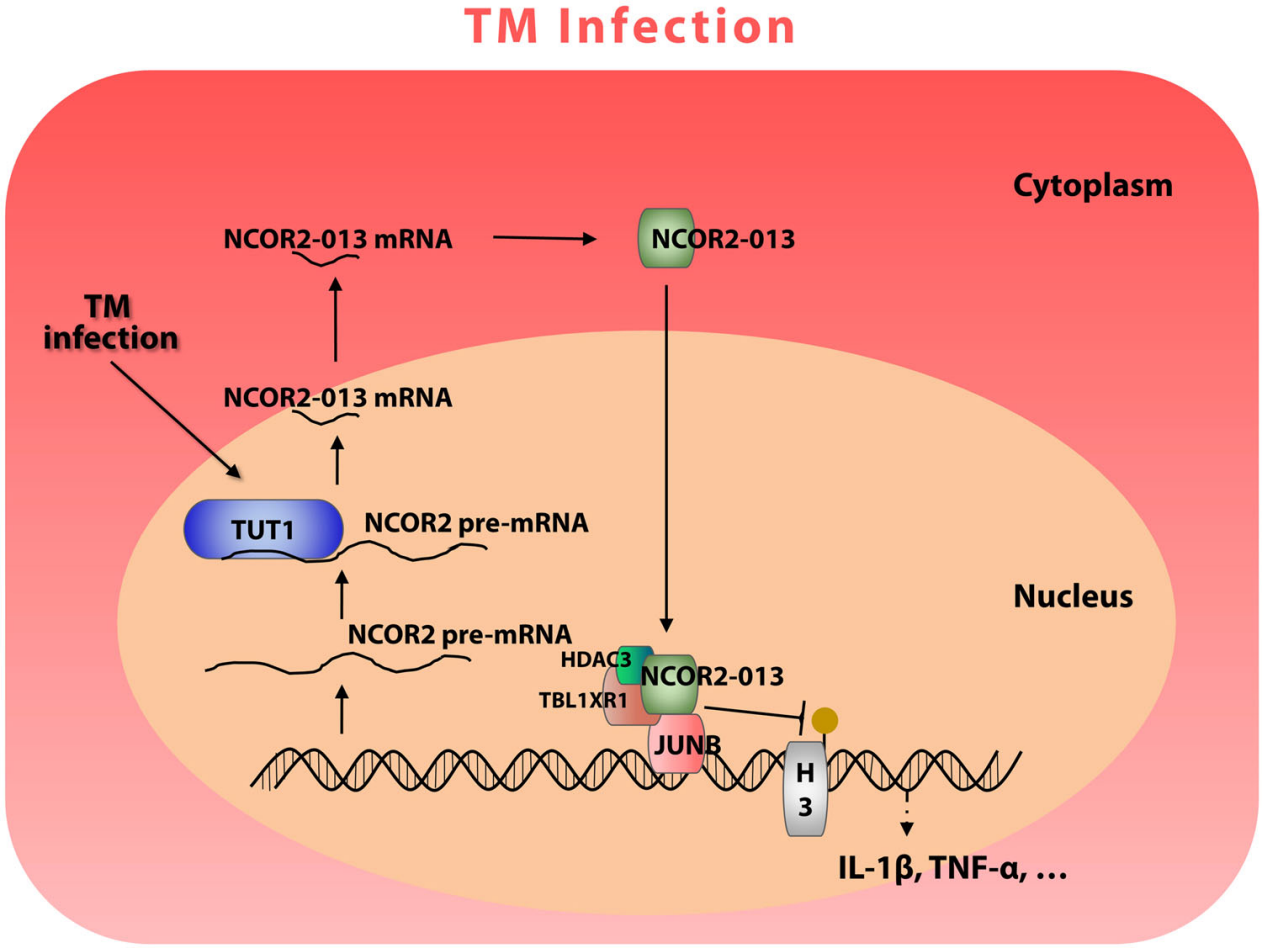
Schematic diagram: TUT1-mediated alternative splicing produces a truncated protein NCOR2-013 that suppresses *T. marneffei*-stimulated macrophage inflammation. *T. marneffei* infection of macrophages promoted AS of *NCOR2*, in which TUT1 is an AS regulator, and NCOR2-013, a truncated protein of NCOR2/SMRT, was significantly upregulated after *T. marneffei* infection. Mechanistic analysis reveals that NCOR2-013 forms a co-inhibitory complex with TBL1XR1/TBLR1 and HDAC3. Consequently, this complex inhibits the JunB-mediated transcriptional activation of pro-inflammatory cytokines by suppressing histone acetylation, thereby regulating the expression of inflammatory factors such as *IL-1β* and *TNF-α*.

We then investigated the effect of TUT1 overexpression or knockdown on *T. marneffei* clearance in THP-1 macrophages. As a result, more *T. marneffei* were detected in TUT1-overexpressing THP-1 macrophages at 24 h post-infection (Fig. 7j), and pro-inflammatory factors (*TNF-α, IL-1β*) were down-regulated at 24 h post-infection (Fig. 7l). However, the opposite trend was observed in TUT1-knockdown macrophages (Fig. 7k, m), suggesting that TUT1 mediates *T.marneffei-*impaired macrophage clearance of fungi. Collectively, these data suggest that *T. marneffei* suppresses macrophages inflammation by producing the truncated protein NCOR2-013 via TUT1-regulated AS (Fig. 8).

## Discussion

The diverse products resulting from AS play a crucial role in biological functions, particularly in the regulation of cellular immune responses, which is supported by increasing evidence^31,32^. In this study, we reported drastic changes in AS event profile of macrophage genes upon *T. marneffei* infection by high-throughput RNA sequencing, and found that *NCOR2-013* transcript, a truncated variant of NCOR2 / SMRT, was significantly upregulated during *T. marneffei* infection. Mechanistically, we found that NCOR2-013 inhibits JunB-mediated transcriptional activation of pro-inflammatory factors *TNF-α* and *IL-1β*. Furthermore, we identified an AS regulator RBP, TUT1, which is involved in promoting *T. marneffei* immune evasion via regulation of NCOR2-013 production. Taken together, these findings elucidate for the first time how *T. marneffei* escapes macrophage killing from the perspective of cellular AS.

During the evolution of pathogens, some evasion strategies have been evolved to evade the host innate immunity. The most common strategy is to inhibit innate immune pathways through the interactions between pathogen virulence proteins and host proteins^11,33–35^. In recent years, growing evidence supports the important role of AS in regulating innate immunity^31,32^. Thousands of AS events have been detected in human dendritic cells and macrophages caused by the bacterial challenges^23,36,37^. To date, little is known about the landscape of AS events associated with *T. marneffei*-infected macrophages. Therefore, we performed a comprehensive analysis of AS events in human THP-1 macrophage challenged by *T. marneffei* infection, and found 1757 different AS events in *T. marneffei*-infected macrophages compared to control cells, which is comparable to bacterial infections, such as *M. tuberculosis*^23^, suggesting increased AS occurs upon pathogen-host interactions. Subsequently, host genes that harbor differential AS events were highly enriched in pathogen immune-related signaling pathways, indicating AS of these genes may serve as another mechanism for macrophages to resist *T. marneffei* infection or *T. marneffei* immune escape. Finally, 6 immune-related regulators were found to have the significantly different AS events, suggesting that AS is involved in the key steps of regulating *T. marneffei* immune response.

Typically, protein-coded genes can produce alternatively spliced variants of multiple transcripts, including some non-functional and/or truncated variants of the proteins that mediate different biological functions. The reasons for the significant biological differences of these alternately spliced variants may include that the truncated regions have certain biological functions. In immune cells, the expression levels of different alternative spliced variants may have significant effects on the activation of signaling pathways, immune status, and antibacterial capacity. For instance, MyD88L and MyD88S are long isoforms and short isoforms of MyD88 proteins, which play a role in activating and inhibiting innate immunity, respectively^38,39^. Dengue virus deregulates innate immune response via inducing *SAT1* exon 4 skipping to produce an isoform of SAT1^22^. In addition, up-regulation of the *RAB8B* truncated variant helps *M. tuberculosis* evade killing effect of macrophage phagosome^23^. However, in the field of fungal-host interactions, spliced variants have been poorly studied.

In the present study, we identified an alternative spliced variant of NCOR2/SMRT, NCOR2-013, in *T. marneffei*-infected macrophages. Of note, normal THP-1 macrophages barely express NCOR2-013 (Fig. 3b), suggesting NCOR2-013 may not be a protein that maintains the normal cell functions. In this case, *T. marneffei* may use NCOR2-013 to implement some biological processes that favor its survival in macrophages. NCOR2/SMRT is a large nuclear receptor that form a co-repressor in the nucleus with a molecular weight up to 274 kDa^25^. Interestingly, as one of the 25 isoforms of NCOR2/SMRT, NCOR2-013 has a molecular weight of 17 kDa, which is only one-sixteenth of NCOR2/SMRT^40,41^. Structurally, NCOR2-013 retains only 4 exons compared with NCOR2/SRMT. Nevertheless, NCOR2-013 still retains some biological functions similar to NCOR2/SMRT. NCOR2-013 acts as part of a multisubunit complex that includes TBL1XR1/TBLR1 and HDAC3 to inhibit chromatin unfolding and specifically block JunB-mediated basal transcriptional activity of pro-inflammatory genes^25,42,43^. The *T. marneffei*-induced short isoform functions similarly to the long isoform, but differently from those of the other pathogens mentioned above^23,36,37^. Our data demonstrate that NCOR2-013 has a highly effective anti-inflammatory function at such a small molecular weight. Co-IP results showed that NCOR-013 did not bind to NCOR2/SMRT, indicating that NCOR2-013 had an anti-inflammatory effect independent of NCOR2/SMRT. More interestingly, even overexpression of JunB did not reverse the anti-inflammatory response of NCOR2-013, which implies a strong affinity of NCOR2-013 for JunB or a role for JunB in transcriptional regulation through phosphorylation of JunB (p-JunB). Furthermore, LPS, but not IFN-γ, partially overcomes NCOR2-013-mediated inhibition of inflammatory response in macrophages, suggesting that NCOR2-013 may only mediate inhibition of specific inflammatory pathways. Previous studies have demonstrated that the IFN-γ-induced signaling pathway selectively targets SMRT through the MAPK signaling pathway. Upon activation of MAPK/ERK kinase, SMRT undergoes phosphorylation and is subsequently redifferentiated from the nucleus to the cytoplasm. This process may lead to the relief of repression on target genes that are specific to SMRT and NCoR-SMRT response. In contrast, TLR4 induces the removal of NCoR from target genes through the p65 subunit of NF-κB^25^.. Collectively, from our results, NCOR2-013 may have similar biological effects as NCRO2/SMRT, but with its own regulatory features. It will be an interesting topic for future studies to uncover the specific molecular mechanisms responsible for the action of NCOR2-013.

In terms of epigenetic mechanism, the NCOR2-013 regulatory complex depends on HDAC3 to inhibit histone acetylation, thereby inhibiting the transcriptional activation response of pro-inflammatory factors^25^. HDAC3 is a chromatin-modifying enzyme that silences transcription via inhibition of histones acetylation. Traditionally, HDAC3 requires interacts with NCRO2/SMRT to engage its catalytic activity^44–46^. Interestingly, we found that HDAC3 can interact with NCOR2-013 rather than NCOR2 / SMRT to exert an inhibitory effect, which is similar to the results of a recent study indicating that HDAC3 is recruited to ATF2-bound sites in the absence of NCOR2 / SMRT^47^. In this study, we found that *T. marneffei*-infected NCOR2-013-overexpressing macrophages have more HDAC3 protein enrichment in promoter regions of pro-inflammatory factors, and conversely, significantly reduced histone acetylation levels in these regions, which are similar to results of other studies^48^, demonstrating that HDAC3 mediates inflammatory reactivity in macrophages.

AS processes are controlled by splicing regulators, mainly RBPs. In this study, TUT1 was identified as an anti-inflammatory factor that was important for NCOR2-013 production and *T. marneffei* immune evasion. TUT1 is a nucleotidyl transferase that functions as both a terminal uridylyltransferase and a nuclear poly(A) polymerase, which can specifically add and remove nucleotides from the 3’ end of small nuclear RNAs as well as select mRNAs, thereby affecting gene expression^49,50^. However, little is known about the role of TUT1-mediated AS in immune responses. The RIP result demonstrated that TUT1 is capable of interacting with pre-*NCOR2* mRNA, indicating that TUT1 is involved in the regulation of NCOR2-013 production. TUT1 overexpression increased the expression of NCOR2-013 and decreased the expression of pro-inflammatory factors as well as antifungal capacity of macrophages, partly by regulating AS of *NCOR2*. On the contrary, TUT1 knockdown led to the opposite results, suggesting that TUT1 acts as a splicing regulatory factor that promotes *T. marneffei* immune escape.

*T. marneffei* is a dimorphic pathogenic fungi that grows filamentously at 25 °C and yeast at 37 °C. The transformation process from conidia to yeast may be an important virulence mechanism of *T. marneffei* against killing by host phagocytes. Previous studies have demonstrated that conidia replicate intracellularly by binary fission and express yeast-phase specific antigen within 24 h of phagocytosis^51–53^. In contrast, in the absence of macrophages, *T. marneffei* conidia take at least 3 weeks to fully convert to the yeast form^54^. Given that it is unclear whether the yeasts produced by these two transformation processes are different, we prioritized the use of a conidia-cell co-culture for at least 24 h approach, which more closely mimics the in vivo environment. Nonetheless, the potential that this phenomenon of *T. marneffei* suppressing the host macrophage inflammatory response could be affected by conidia-dependence or conidia-yeast intermediate stages cannot be disregarded and warrants further investigation.

In addition, this study has several limitation that need to be considered. First, our current work leans on overexpression systems to investigate the function of NCOR2-013, this approach does not provide direct evidence that the intact form suppresses cytokine production and bactericidal activity. Future research endeavors will seek to employ more precise techniques to provide a more comprehensive understanding of the biological role of NCOR2-013. Second, how *T. marneffei* infection affects TUT1 expression still needs to be systematically investigated. Third, HIV infection was not considered in this study, but NCOR2-013-mediated *T. marneffei* survival may be different in the context of HIV infection, which should be an interesting issue for the future studies. Finally, while preliminary data indicate that LPS, a component of the bacterial cell wall, enhances the expression of NCOR2-013 and TUT1 in macrophages (Supplementary Fig. 4), suggesting that such an induction is not specific to *T. marneffei* alone. Thus, it remains essential to explore which pathogens can specifically induce TUT1-mediated NCOR2-013 AS events and to understand the underlying molecular mechanisms.

In summary, we systematically analyzed AS events in *T. marneffei*-infected macrophages and identified TUT1 as the splicing regulator that was important for the NCOR2-013 production and *T. marneffei* immune evasion. TUT1 inhibits inflammatory response via inducing AS of NCOR2 to produce a novel transcript, NCOR2-013. The truncated protein NCOR2-013 acts as an important *T. marneffei* immune escape-related factor in macrophages through recruiting TBL1XR1/TBLR1 and HDAC3 to form a regulatory complex to inhibit histone acetylation modification, which specifically blocks JunB-mediated transcriptional activity of pro-inflammatory genes (Fig. 8). TUT1 and NCOR2-013 represent the potential targets for the development of mechanism-based strategies for the prevention of talaromycosis.

## Methods

### Reagents

Human recombinant IFN-γ and GM-CSF antibodies were purchased from Solarbio Science & Technology (China), and the LPS (from *E. coli*) was from Sigma-Aldrich (USA). The IRDye 680RD donkey anti-mouse and IRDye 800CW donkey anti-rabbit antibodies were purchased from LI-COR Biosciences (USA). Anti-rabbit IgG (H + L), F(ab’)2 fragment (Alexa Fluor® 647 Conjugate), anti-FLAG (D6W5B), anti-PCNA (D3H8P), anti-β-actin (8H10D10), anti-normal Rabbit IgG, anti-HDAC3 (D2O1K), anti-TBL1XR1/TBLR1 (D4J9C), anti-JunB (C37F9), anti-phospho-JunB (Thr102 / Thr104) (D3C6), anti-JunD (D17G2), anti-c-Jun (60A8), anti-phospho-c-Jun (Ser73), anti-acetyl-histone H3 (Lys27) (D5E4), and anti-GAPDH (D16H11) antibodies were purchased from Cell Signaling Technology (USA). Anti-NCOR2 antibody was obtained from Novus Biologicals (USA). Anti-TUT1 (PA5-50151) antibody was obtained from Invitrogen (USA). For western blot, all primary antibodies were diluted at 1 : 1000 and secondary antibodies at 1 : 20,000. For immunofluorescence, the primary antibody was diluted 1 : 200. For IP, RIP, and ChipIP, 1 µg of primary antibody was used to precipitate proteins.

### *T. marneffei* strain and culture conditions

*T. marneffei* strain (ATCC18224) was obtained from American Type Culture Collection (ATCC) and used in all infection experiments. *T. marneffei* was cultivated on potato dextrose agar (Sigma-Aldrich, USA) and grew at 27 °C for 7–10 days. The conidia were harvested as previously described^52,53,55^. Briefly, colonies of *T. marneffei* were flooded with PBS, and the conidia were filtered through sterile glass wool (Corning, USA) to remove clumps and fragments. The conidia-enriched supernatant was collected by centrifugation at 14,000 × g for 3 min and enumerated using a hemacytometer. Conidia were resuspended in sterile PBS at 10^7^/mL and stored at 4 °C until use. The collected conidia were tested for viability after each collection and used within 2 weeks.

### THP-1 macrophages and human monocyte-derived macrophages (hMDMs)

THP-1 cells were obtained from ATCC and cultured in RPMI 1640 medium (Solarbio Science & Technology, China) containing 10% fetal bovine serum (FBS, Gibco, USA) and 1% penicillin / streptomycin (Solarbio Science & Technology, China). The cells were allowed to differentiate in macrophages with the treatment of 100 ng/mL phorbol myristate acetate (Sigma-Aldrich, USA) for 48 h. Human monocyte-derived macrophages (hMDMs) were obtained from human peripheral blood mononuclear cells (PBMCs) of healthy adult donors. Briefly, PBMCs were isolated by density gradient centrifugation (Ficoll 1.077 g/mL, Sigma, USA) and cultured in Dulbecco’s Modified Eagle’s Medium (DMEM, Solarbio Science & Technology, China) supplemented with 10% FBS and 1% penicillin / streptomycin. To induce differentiation to macrophages, PBMCs were cultured for 7 days in the presence of 25 ng/mL recombinant human macrophage colony-stimulating factor (rh-MCSF, Solarbio Science & Technology, China). The cells were > 95% CD14^+^ cells, as determined by flow cytometry with an anti-CD14 antibody (BD Biosciences, USA). All cells were cultured at 37°C containing 5% CO_2_. In this study, the hMDMs were differentiated from PBMCs isolated from three healthy blood donors.

### *T. marneffei* infection of macrophages

*T. marneffei* infection of macrophages followed the method described in previous studies (56). Briefly, macrophages were seeded at a density of 2 × 10^6^ cells/well in 6-well plates, 1 × 10^6^ cells/well in 12-well plate, or 1 × 10^5^ cells/well in 96-well plates, and then were infected with *T. marneffei* conidia at a multiplicity of infection (MOI) of 10. The infection lasted for 2 h, after which the medium was discarded and the cells were washed with pre-warmed PBS (37 °C). The infected cells were then cultured in 10% DMEM until the time required for the subsequent experiments.

### Lentiviral transfection

Lentiviral siRNA vector system GV248 (hU6-MCS-Ubiquitin-EGFP-IRES-puromycin) and lentiviral overexpression vector system GV208 (Ubi-MCS-3FLAG-EGFP) were constructed, packaged, and purified by GeneChem (China), and manipulated according to the protocols provided by the manufacturer. Briefly, THP-1 cells were seeded in 96-well plates and cultured overnight prior to transfection, and hMDMs were differentiated from PBMCs in 96-well plates. Subsequently, siRNA-TUT1 lentivirus, Flag-TUT1-overexpressing lentivirus, Flag-NCOR2-013-overexpressing lentivirus and their negative control (NC) empty lentivirus vector were added to serum-free RPMI 1640 medium (for THP-1 cells) or DMEM medium (for THP-1 macrophages and hMDMs) media, respectively. The THP-1 cells or THP-1 macrophages were infected with an MOI of 30 and hMDMs were infected with an MOI of 50 according to the manufacturer’s protocols. The transfection lasted for 48 h, and the medium containing lentivirus was then removed and fresh complete medium was added. The sequences of siRNA-TUT1 were designed as follows: siRNA-TUT1-F: 5’-CCGGGATCTTGACCTCTTCTTGGACTCGAGATCCAAGAAGAGGTCAAGATCTTTTTG-3’, siRNA-TUT1-R: 5’-AATTCAAAAAGATCTTGACCTCTTCTTGGATCTCGAGATCCAAGAAGAGGTCAAGATC-3’.

### Reverse transcription quantitative PCR (RT-qPCR)

Total cellular RNA was extracted using Trizol and chloroform reagents (Invitrogen, USA), followed by precipitation in isopropyl alcohol and 75% ethanol. Reverse transcription was performed using a reverse transcription kit (TAKARA, Japan). RT-qPCR was performed using SYBR Premix ExTaq^TM^ (TAKARA, Japan) by StepOnePlus real-time PCR system (Thermo Fisher Scientific, USA). Data were analyzed with 2^−ΔΔ Ct^ method. Relative gene expression was calculated as the ratio of target gene expression versus reference *GAPDH* gene expression. Primers for *NCOR2* were designed using primer-BLAST and Primer Premier 5 software to amplify the exon region of *NCOR2*. Primers for *IL-1β, TNF-α*, etc were from Primer Bank. All primers targeted exon region and were optimized before RT-qPCR experiments. Primer sequences are provided in Supplementary Table 3. The primer design principles and primer sequences for calculating NCOR2-013 expression levels are shown in Supplementary Fig. 5 and Supplementary Table 4.

### Western blot

Total proteins, nuclear proteins, or cytoplasmic proteins were extracted using the cell lysis buffer (Cell Signaling Technology, USA), nuclear protein and cytoplasmic protein extraction kit (Beyotime, China), respectively, according to the manufacturer’s instructions. Protein concentration was measured using a BCA protein assay kit (Beyotime, China). Total proteins, nuclear proteins, or cytoplasmic proteins were separated by 10–12% SDS-PAGE (BOSTER, China) and transferred to PVDF membranes (Bio-Rad, USA). After blocking with 5% nonfat milk (BD Biosciences, USA) for 1 h, the membranes were incubated with primary antibody and 5% nonfat milk overnight at 4 °C (the dilution ratio of primary antibodies is 1 : 1000). The membranes were then washed three times with TBST (10 mM Tris-HCl, 150 mM NaCl, 0.05%V/V Tween-20) and incubated with the corresponding secondary antibody for 1 h (the dilution ratio of secondary antibodies is 1 : 20,000). Images were performed using the Odyssey CLX two-color infrared laser imaging system (LI-COR Biosciences, USA). The densitometric analysis of blots was performed by Image Studio Ver 5.2 software (LI-COR Biosciences, USA). The values were normalized to those of control β-actin or GAPDH (for total protein, cytoplasmic protein), or PCNA (for nuclear protein).

### Determination of colony-forming units (CFU)

CFU method was used to quantify the amount of *T. marneffei* in macrophages as previously described^56–59^. Briefly, the culture supernatant was discarded, and the macrophages were washed twice with PBS and then lysed with sterile water to release the fungus. The intracellular *T. marneffei* were collected by centrifugation (14,000 g, 5 min) and suspended with sterile water to 200 μL. Four gradient serial dilutions (10^0^, 10^−1^, 10^−2^, 10^−3^) were performed, and 5 μL of each diluted sample was plated onto YPD agar. CFUs were counted after 24 h incubation at 30 °C. The fungal load was calculated as number of colonies × dilution gradient × 40, where 40 is the ratio of 200 μL to 5 μL.

### Immunofluorescence

Immunofluorescence was performed to detect the subcellular localization of NCOR2-013. Flag-NCOR2-013-overexpressed THP-1-derived macrophages were plated onto a cover slip. The cells were fixed with 4% paraformaldehyde for 20 min, washed 3 times with PBS, and then incubated with 1% Triton X-100 PBS solution for 20 min at room temperature and washed 3 times with PBS. After blocking with 5% nonfat milk for 1 h, the cells were incubated with 1 : 200 primary anti-Flag antibody at 4 °C overnight, washed with PBS, and then incubated with specific fluorescence-conjugated secondary antibody for 1 h at room temperature. Finally, DAPI (4, 6-diamidino-2-phenylindole) was added to label nuclei. Immunofluorescence signals were detected using fluorescence microscopy (Nikon, Japan).

### Cytometric bead array (CBA)

CBA was used to determine the levels of secreted TNF-α and IL-1β according to the manufacturer’s instructions (BD Biosciences, USA). Briefly, the culture supernatants were collected, and 50 µL of supernatant was mixed with 50 µL capturing beads and 50 µL detection reagents of the kit, then incubated for 2 h at room temperature. The beads were washed and re-suspended in 300 µL wash buffer and analyzed by flow cytometry (Beckman Coulter, USA).

### Immunoprecipitation (IP) and Co-Immunoprecipitation (Co-IP)

Cell lysates were prepared in cell lysis buffer (for immunoprecipitation, Cell Signaling Technology, USA) or Co-IP buffer (for co-immunoprecipitation, Cell Signaling Technology, USA) supplemented with phosphatase and protease inhibitors. Lysates were pre-cleared with anti-mouse or anti-rabbit IgG agarose beads for 30 min. Proteins were precipitated with 1 µg of primary antibodies for 1 h and subsequently collected with anti-mouse or anti-rabbit IgG agarose beads by overnight incubation. Bead-protein complex was washed 3 times with PBS, and then the immunoprecipitates were subsequently eluted and separated by SDS-PAGE gel electrophoresis, followed by western blot.

### Chromatin immunoprecipitation (ChIP)

Chromatin immunoprecipitation was carried out using agarose ChIP kit (Thermo Scientific, USA) according to the manufacturer’s instructions. Immunoprecipitation was performed with 1 µg of each antibody or negative control IgG. Real-time PCR was performed on the purified DNA and input DNA using SYBR Premix ExTaq^TM^ (TAKARA, Japan) by StepOnePlus real-time PCR system (Thermo Fisher Scientific, USA). The primer sequences are provided in Supplementary Table 5. The results were computed as percent antibody bound per input and data were displayed after subtracting control IgG values. Data from three independent experiments were collected, and mean value and standard deviation (SD) were presented.

### RNA-binding protein Immunoprecipitation (RIP)

RIP assay was performed using RIP RBP Immunoprecipitation kit (Sigma-Aldrich, USA) according to the manufacturer’s protocol. Briefly, cells were collected by using RIP lysis buffer and incubated with beads and antibody complexs overnight. After washing 3 times, RNAs that binding to protein were eluted and qRT-PCR was used to detect target RNAs co-immunoprecipitated with the antibody. Meanwhile, The quantification of target RNAs was carried out by agarose gel electrophoresis. Specifically, a 1% agarose gel in 1X TAE buffer was prepared and loaded with cDNA samples that were reverse transcribed from the target RNAs and mixed with loading buffer. The gel was then subjected to electrophoresis at 100 V for 45 min. Ethidium bromide was used to stain the gel, which was subsequently visualized using a UV transilluminator.

### Liquid chromatography/tandem mass spectrometry (LC-MS / MS)

Protein samples were separated by 10% SDS-PAGE and stained with Coomassie Brilliant Blue. The gel bands were manually excised and and decolorize with decolorizing solution. A solution of tryptic peptides were then obtained by proteolysis and transferred to vials for LC-MS / MS analysis. LC-MS / MS analysis was performed using a Q-Exactive mass spectrometer (Thermo Fisher Scientific, USA) in tandem with a a nano Acquity UPLC system (Waters, USA). A total of 3 μl was loaded (Thermo Fisher Scientific Acclaim PepMap trap column, C18, 100 μm × 25 cm, and Acclaim PepMap self-packed analytical column, C18, 75 μm × 25 cm), and the eluted peptides were introduced directly into the linear ion trap mass spectrometer at a flow rate of 200 nL/min and a spray voltage of 2.0 kV. The MS and MS/MS data were acquired using Xcalibur (Thermo Fisher Scientific, USA).

### RNA-seq and function enrichment analysis

Total RNA was extracted using Trizol regent (Invitrogen, USA) according to the manufacture’s protocol. RNA quality was assessed on Agilent 2100 Bioanalyzer (Agilent Technologies, USA). Then, mRNA was enriched by Oligo (dT) beads, while mRNA was enriched by removing rRNA by Ribo-ZeroTM magnetic kit (Epicenter, USA). Subsequently, the enriched mRNA was fragmented into short fragments using fragmentation buffer and reverse transcribed into cDNA. The cDNA was purified with QIAquick PCR extraction kit (Qiagen, Netherlands) and ligated to Illumina sequencing adapters. The library preparation and RNA-seq was performed by GeneChem (China). In this study, a total of 200 ng of RNA in 50 μL was used for preparation of paired-end sequencing libraries. The RNA samples were subjected to quality control, cluster generation and sequencing. The reads were de-multiplexed and converted to FASTA format. Finally, alignment and QC were performed. Transcript counts were normalized using conditional quantile normalization for transcript length and GC content. Paired-end clean reads were mapped to the reference genome using HISAT2. 2.4 to calculate read counts for each unigene. The differential expression between *T. marneffei* uninfected and infected macrophages using a two-sided Wald test in DESeq2 package. The statistical significance was set to |log2 fold of change| > 0.4 and adjusted *P* value < 0.05. The heatmap was constructed using the pheatmap R package to show the expression intensity and direction of the DEGs. The potential function of DEGs with different infection times and modules enrichment analyses were predicted using Clusterprofiler R package. Genes with |log2 fold of change| > 0.4 and adjusted *P* value < 0.05 were selected for Kyoto Encyclopedia of Genes and Genomes (KEGG) and Gene Ontology (GO) analysis. Gene set enrichment analysis (GSEA) was performed using the ‘clusterProfiler’ R package.

### Statistics and reproducibility

The results are expressed as the mean ± standard deviation (SD). The level of statistical significance was set at *P* < 0.05 using an unpaired two-tailed Student’s *t* test. *P < 0.05; **P < 0.01. All statistical analyses were performed using GraphPad Prism software. The sample and replicate size were indicated in the figure legends.

### Ethics statement

The studies involving human participants were reviewed and approved by Ethics and Human Subjects Committee of Guangxi Medical University (Ethical Review No. 20220018). The patients/participants provided their written informed consent to participate in this study. All ethical regulations relevant to human research participants were followed.

### Supplementary information

Supplementary Information

Description of Additional Supplementary Files

Supplementary Data

nr-reporting-summary

## Data Availability

Source data for Figures and Supplementary Figures are provided as Supplementary Data File (Supplementary Data 1) and uncropped Western blots are provided in Supplementary Fig. 6 along with this article. We deposited the raw fastq files in the Sequence Read Archives (SRA) of the National Center for Biotechnology Information (NCBI) under accession number GSE200512, GSE154779 of Bioproject PRJNA824858, PRJNA647412, respectively. Mass spectrometry proteomics data have been deposited on figshare under the following 10.6084/m9.figshare.24160839. All other data are available from the corresponding author on reasonable request.
