# Supplementary material for: Talaromyces marneffei suppresses macrophage inflammation by regulating host alternative splicing
Source: Commun Biol. 2023 Oct 16;6:1046. doi: 10.1038/s42003-023-05409-6 (PMC10579421; doi:10.1038/s42003-023-05409-6)
Supplement: Supplementary file 1 — Supplementary Information [file 42003_2023_5409_MOESM1_ESM.pdf]

Supplementary material for *Talaromyces marneffei* suppresses macrophage inflammation by regulating host alternative splicing

Running title: *T. marneffei* regulates host AS

Wudi Wei[1,2#], Gang Wang[1#], Hong Zhang[2#], Xiuli Bao[1#], Sanqi An[1,2], Qiang Luo[2], Jinhao He[2], Lixiang Chen[1], Chuanyi Ning[2,3*], Jingzhen Lai[2,4], Zongxiang Yuan[1], Rongfeng Chen[1,2], Junjun Jiang[1,2*], Li Ye[1,2*] and Hao Liang[1,2,4*]

[1] Guangxi Key Laboratory of AIDS Prevention and Treatment, School of Public Health, Guangxi Medical University, Nanning 530021, Guangxi, China
[2] Guangxi-ASEAN Collaborative Innovation Center for Major Disease Prevention and Treatment, Life Sciences Institute, Guangxi Medical University, Nanning 530021, Guangxi, China
[3] Nursing College, Guangxi Medical University, Nanning 530021, Guangxi, China
[4] Guangxi Biobank, Life Sciences Institute, Guangxi Medical University, Nanning 530021, Guangxi, China

#Wudi Wei, Gang Wang, Hong Zhang and Xiuli Bao contributed equally to this paper.

*Corresponding authors
Hao Liang, Email: lianghao@gxmu.edu.cn; Tel: 86-771-5323713; Fax: 86-771-5351624;
Li Ye, Email: yeli@gxmu.edu.cn; Tel: 86-771-5334215; Fax: 86-771-5351624
Junjun Jiang, Email: jiangjunjun@gxmu.edu.cn; Tel: 86-771-5334215; Fax: 86-771-5351624

Keywords: *Talaromyces marneffei*, macrophages, NCOR2-013, TUT1, alternative splicing

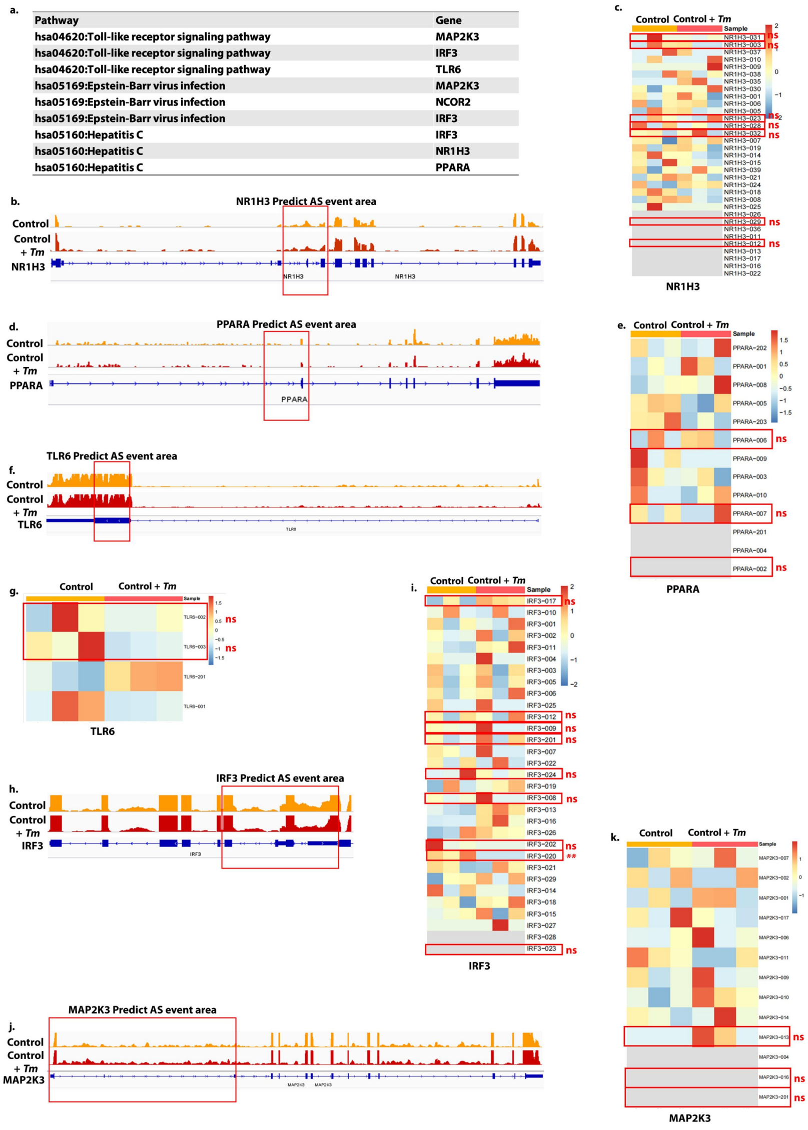

Supplementary Figure 1. The heatmaps and track plots of *NR1H3, PPARA, IRF3, MAP2K3* and *TLR6* transcripts. The human THP-1 macrophages were infected with *T. marneffei* conidia (MOI = 10) for 24 h. (a) Summary of the abundance of 3 KEGG pathways according to ES-type AS genes, with FDR < 0.05 and |Lnclevel| > 0.5 were exhibited. (b, d, f, h, j) Genome tracks plots show the reads from *NR1H3, PPARA, TLR6, IRF3, MAP2K3* genes and predicted AS areas by rMATS in *T. marneffei*-infected or uninfected macrophages at 24 h post infection. Gene coding regions and annotations are indicated in blue at the bottom. The exons and introns are represented by rectangles and straight lines, respectively. The red box represents the site of AS predicted. (c, e, g, k, i) The heatmap exhibits the expression levels of different transcripts of *NR1H3, PPARA, TLR6, IRF3, MAP2K3*. The red boxes represent the transcripts that are produced by predicted AS events and are known to exist. DESeq2 package was used to determine significance, denoted by * (*Padj* < 0.05), ** (*Padj* < 0.01), and ns (not significant). MOI, multiplicity of infection; *Tm, T. marneffei.*

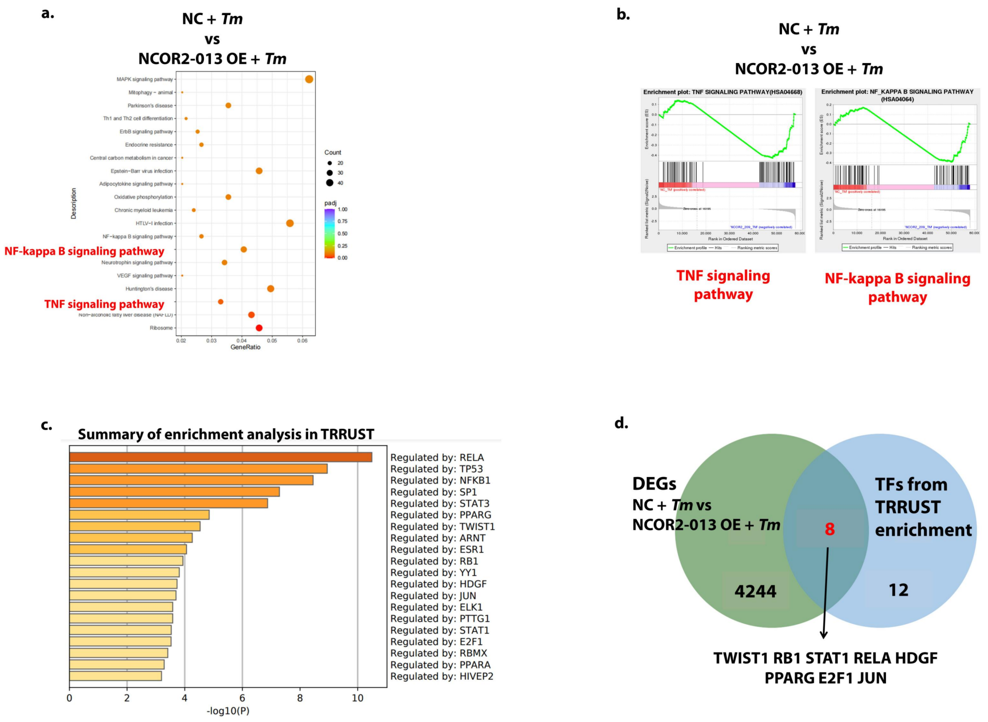

Supplementary Figure 2. Transcriptome profiles of *T. marneffei*-infected NCRO2-013 overexpressing THP-1 macrophages. The NCRO2-013 overexpressing THP-1 macrophages and control cells were infected with *T. marneffei* conidia at a MOI of 10 for 24 h. (a-b) KEGG pathway (a) and GSEA (b) enrichment analysis of DEGs (NCRO2-013 overexpressing THP-1 macrophages vs. control cells). (c)TRRUST analysis was performed to predict the set of transcription factors regulating DEGs. (d) The 20 transcription factors predicted by TRRUST analysis were intersected with DEGs, and 8 transcription factors were obtained, including TWIST1, RB1, STAT1, RELA, HDGF, PPARG, E2F1 and JUN, which may play a role in the regulation of *T. marneffei* infection by NCOR2-013. NC, negative control; NCOR2-013 OE, NCRO2-013 overexpression; TFs, transcription factors; *Tm, T. marneffei.*

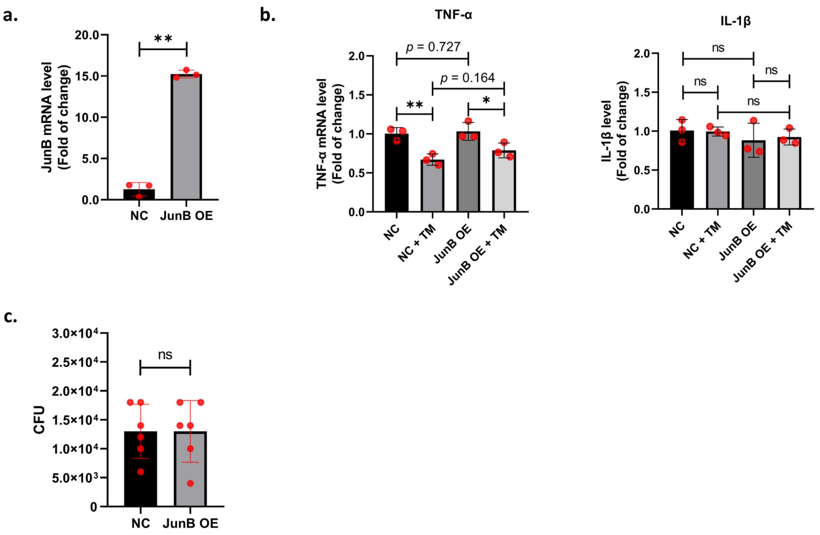

Supplementary Figure 3. JunB overexpression effects on *T. marneffei* infection in THP-1 Macrophages. (a) JunB overexpression in THP-1 macrophages was confirmed by RT-qPCR (n = 3 biological replicates, data are presented as mean values ± SD). (b, c) The JunB overexpressing THP-1 macrophages and control cells were infected with *T. marneffei* conidia (MOI = 10) for 24 h. The expression of *TNF-α and IL-1β* was detected by RT-qPCR (b) (n = 3 biological replicates, data are presented as mean values ± SD). *T. marneffei* CFUs were detected in JunB overexpressing THP-1 macrophages and control cells (c) (n = 6 biological replicates, data are presented as mean values ± SD). Two-tailed Student's *t*-test was used to determine significance, denoted by * ($P < 0.05$), ** ($P < 0.01$), and ns (not significant). MOI, multiplicity of infection; NC, negative control; JunB OE, JunB overexpression; *Tm, T. marneffei.*

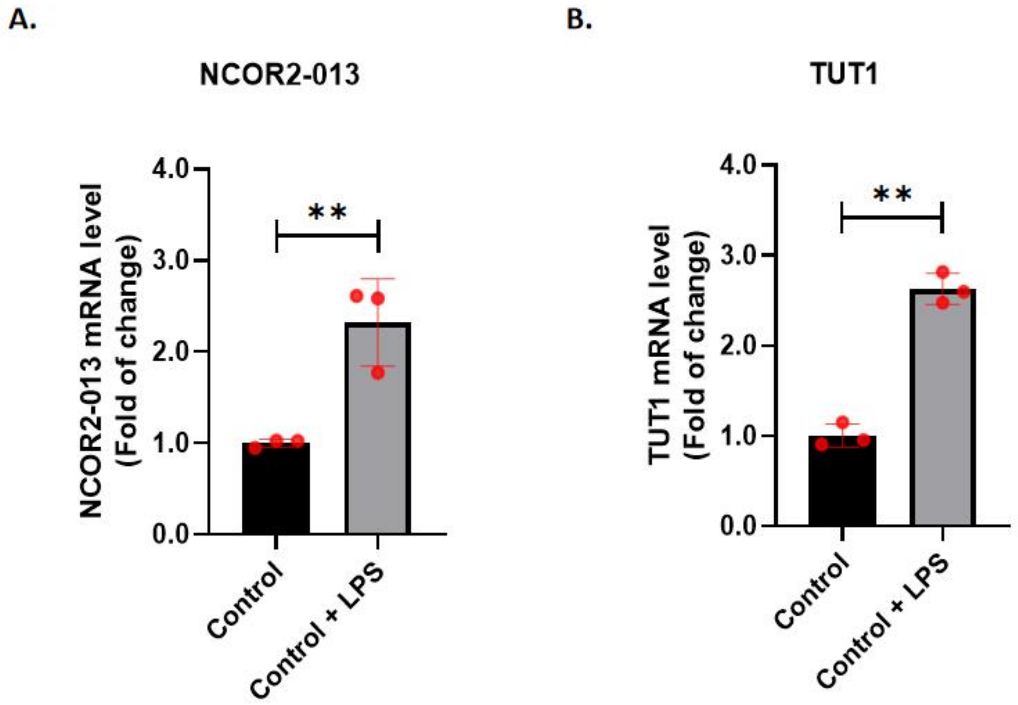

Supplementary Figure 4. THP-1 macrophages were stimulated with LPS (500 ng/mL) for 24 h. The expression of *NCOR2-013* (A) and *TUT1* (B) was detected by RT-qPCR. All data are shown as mean ± SD from three biological replicates (n = 3). Two-tailed Student's *t*-test was used to determine significance, denoted by * ($P < 0.05$), ** ($P < 0.01$), and ns (not significant).

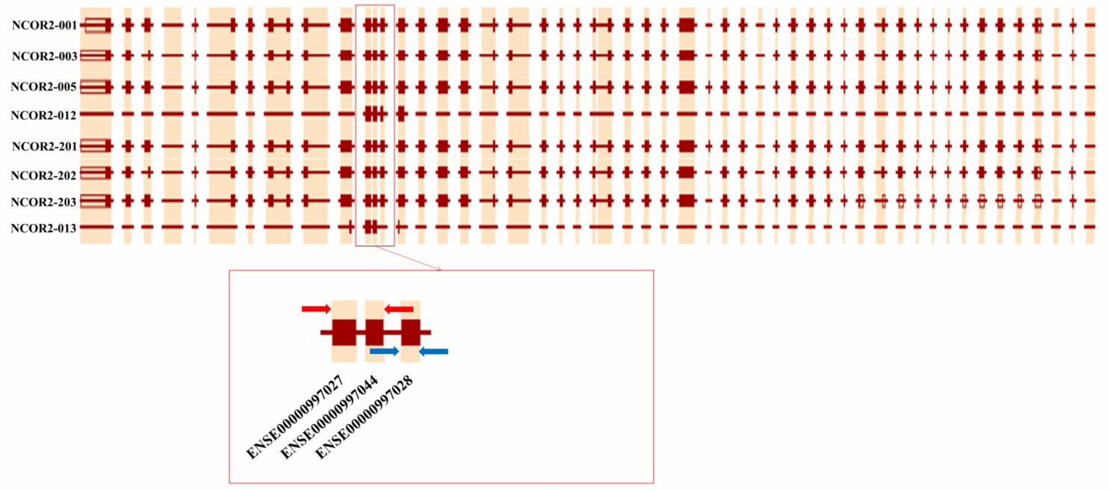

Supplementary Figure 5 . Primer design principles for calculating *NOCR2-013* expression levels. Exons 10 (ENSE00000997027) and 11 (ENSE00000997044) are all included among transcripts of NCOR2, which contain exon 12 (ENSE00000997028), while NCOR2-013 transcript contains only exons 10 and 11, but not exon 12. Therefore, two pairs of primers were designed based on the combined sequence of exons 10 and 11 and the sequence of exon 12. The expression levels of these two pairs of primers were detected by qPCR. Finally, the expression level of exon 12 was subtracted from the expression level of exon 10/11 to indirectly calculate the expression level of NCOR2-013 transcript.

Supplementary Figure 6
Unedited/uncropped western blot gels

Figure 2G

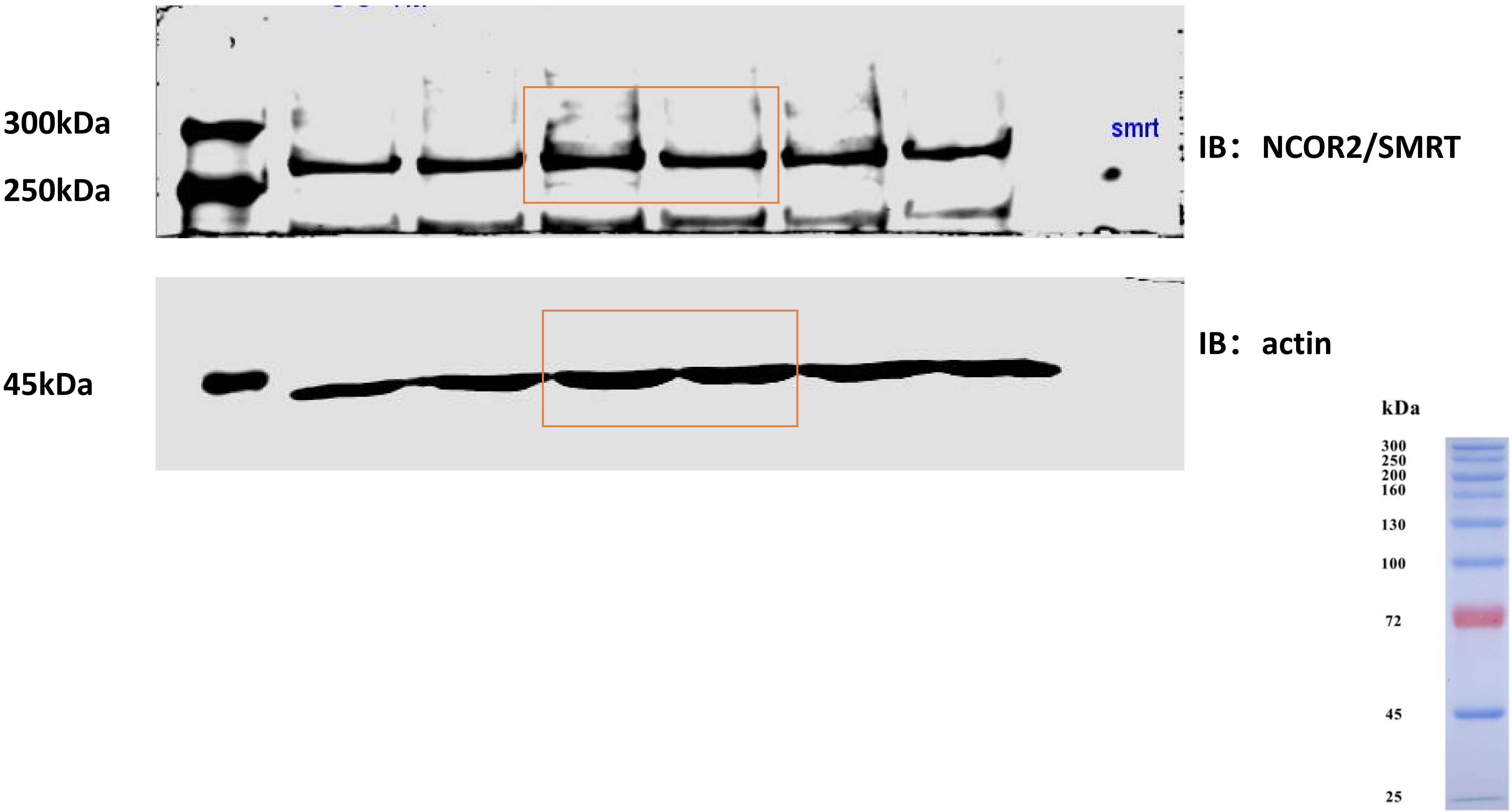

300kDa
250kDa
smrt
IB：NCOR2/SMRT
45kDa
IB：actin
kDa
300
250
200
160
130
100
72
45
25

Figure 3C

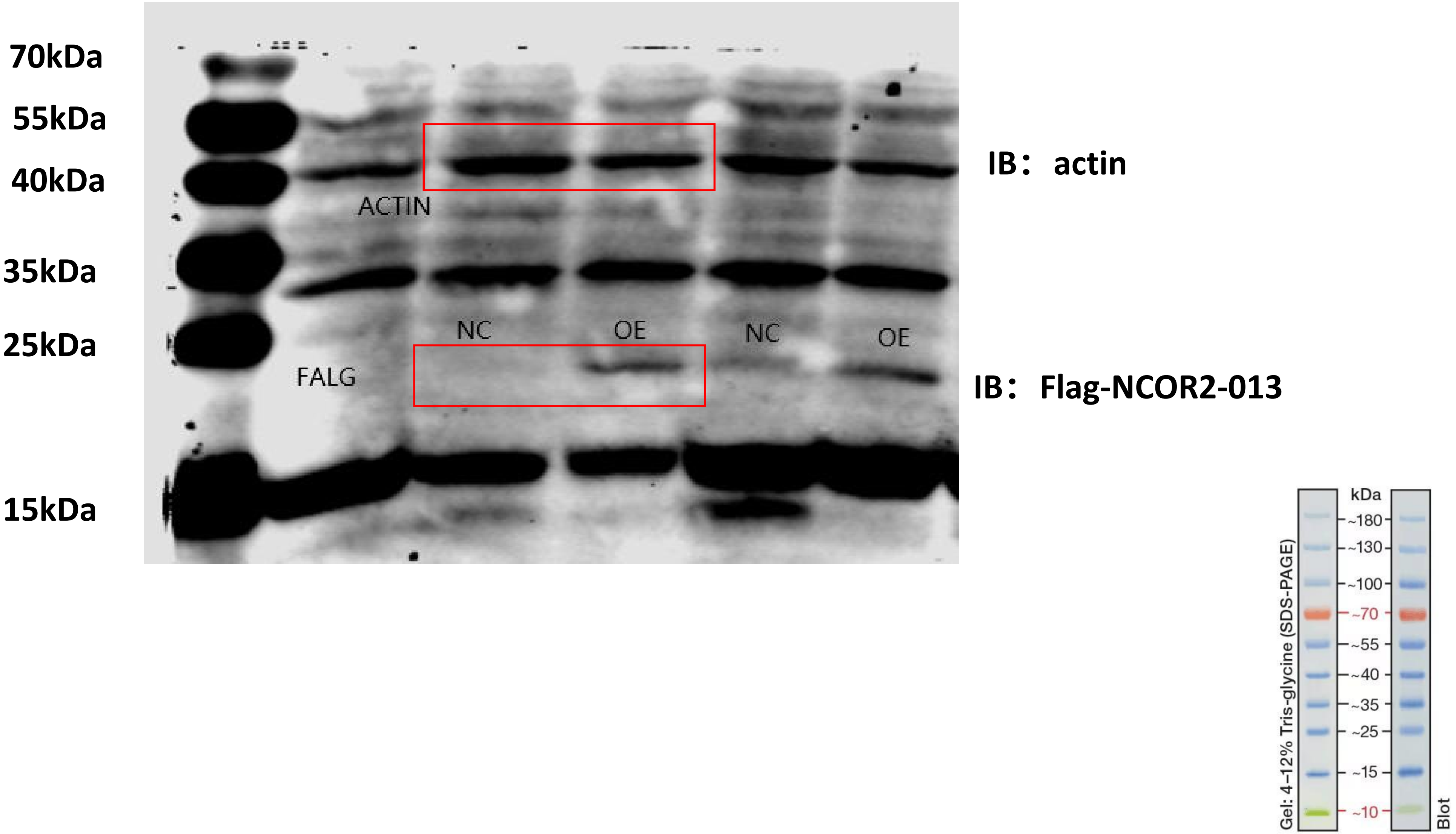
70kDa
55kDa
40kDa
35kDa
25kDa
15kDa
ACTIN
FALG
NC
OE
NC
OE
IB： actin
IB： Flag-NCOR2-013
kDa
~180
~130
~100
~70
~55
~40
~35
~25
~15
~10
Gel: 4–12% Tris-glycine (SDS-PAGE)
Blot

Figure 3E Cytoplasm

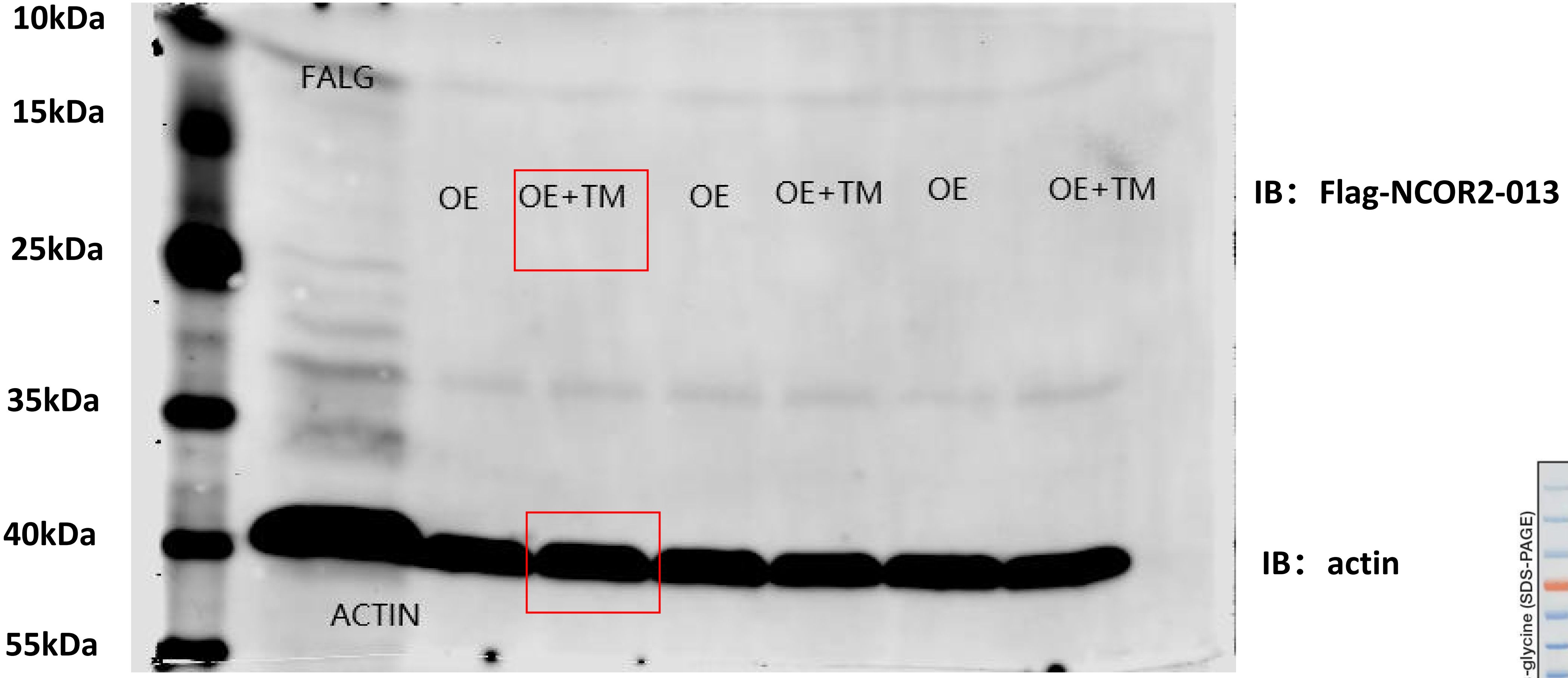

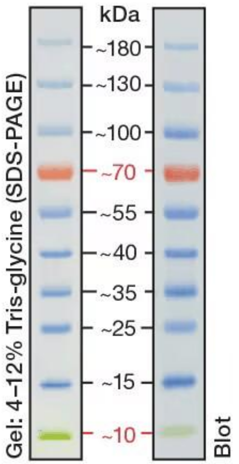

Figure 3E Nucleus

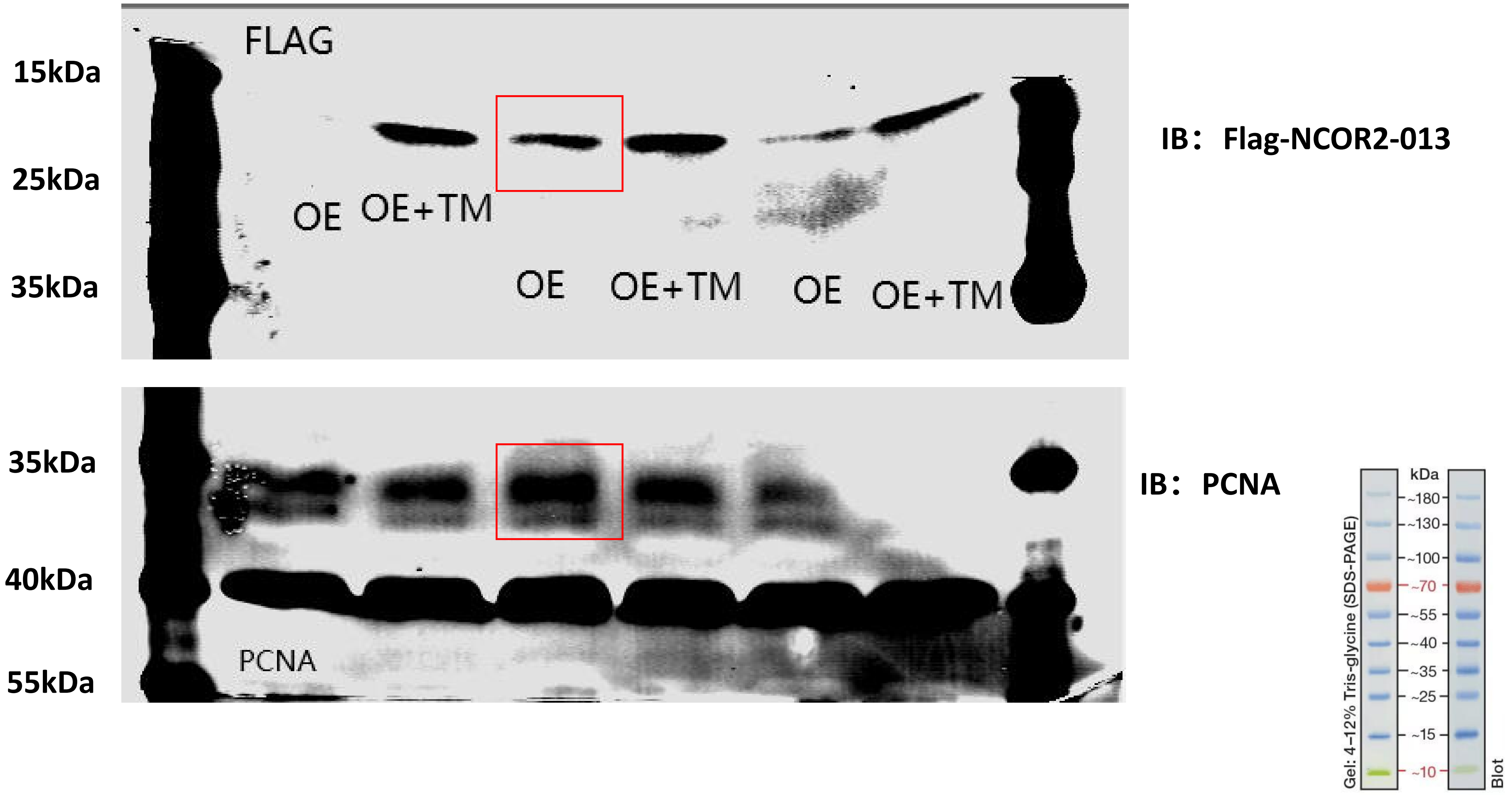

Figure 4B IP: Flag, IB： Flag-NCOR2-013, IB： JunB

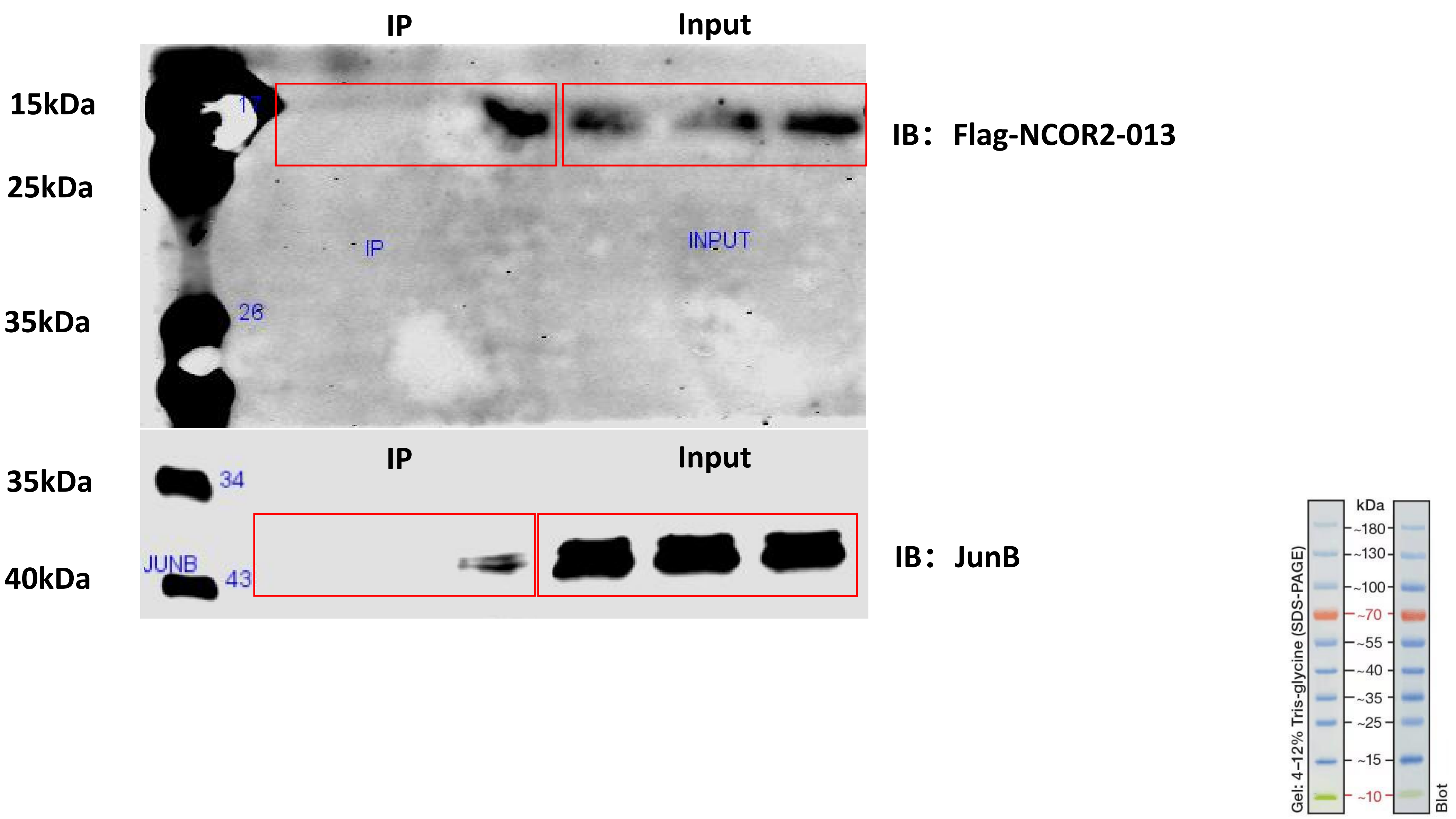

Figure 4B IP: Flag IB： TBL1XR1/TBLR1, IB： HDAC3

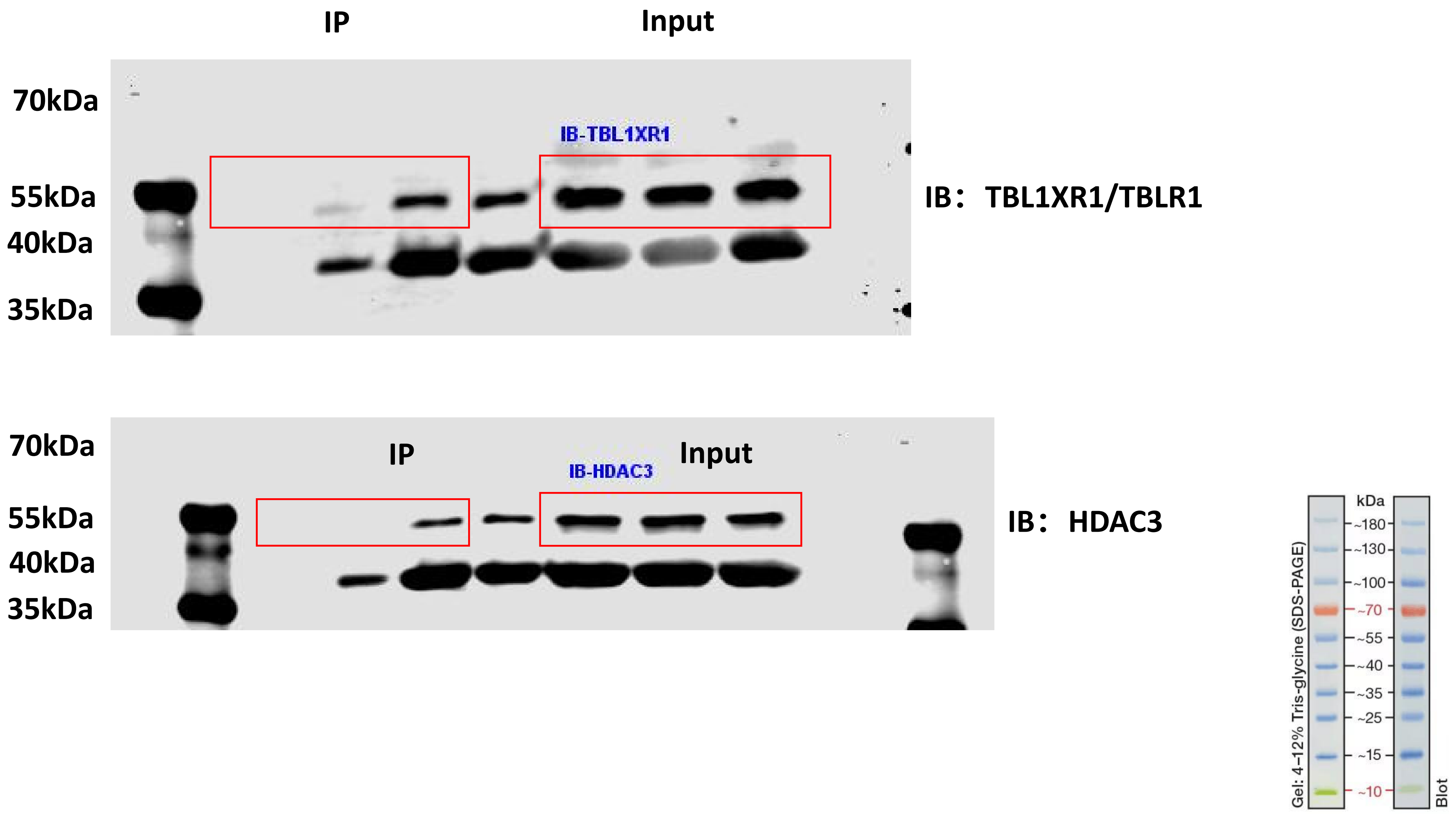

Figure 4B IP: Flag IB：NCOR2/SMRT

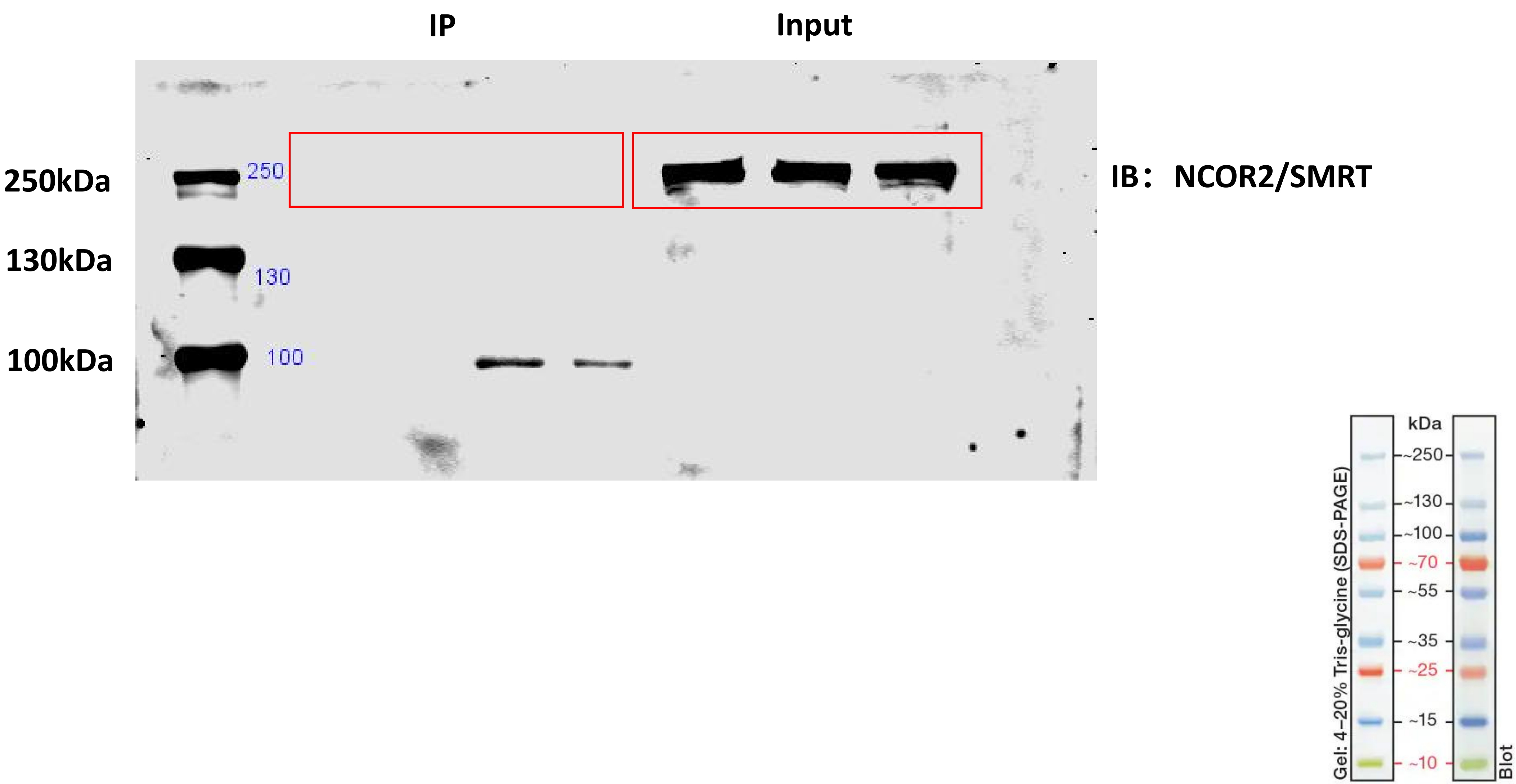

Figure 4C IP: JunB, IB: Flag-NCOR2-013, IB: JunB

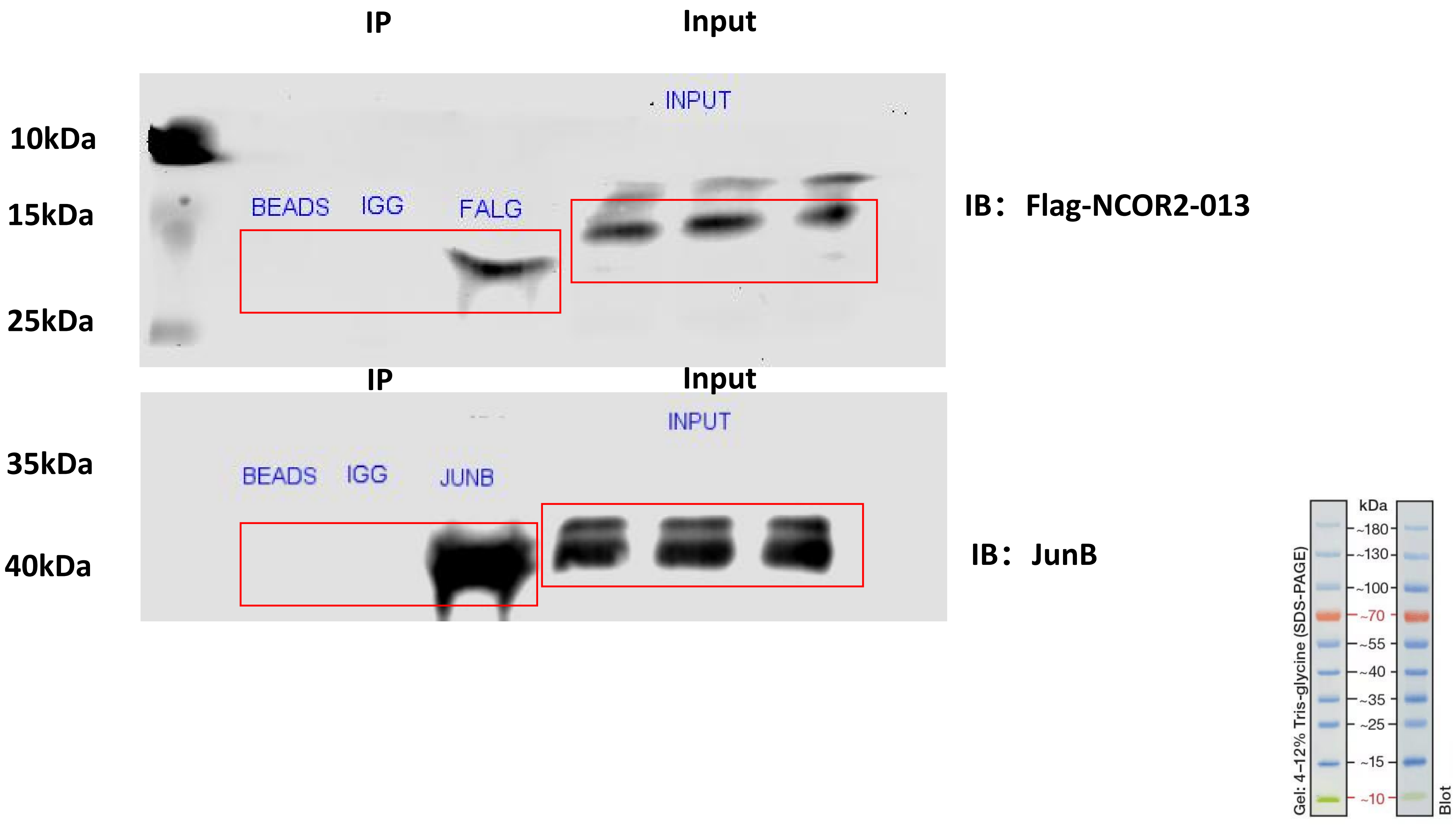

Figure 4C IP: JunB IB： TBL1XR1/TBLR1, IB： HDAC3

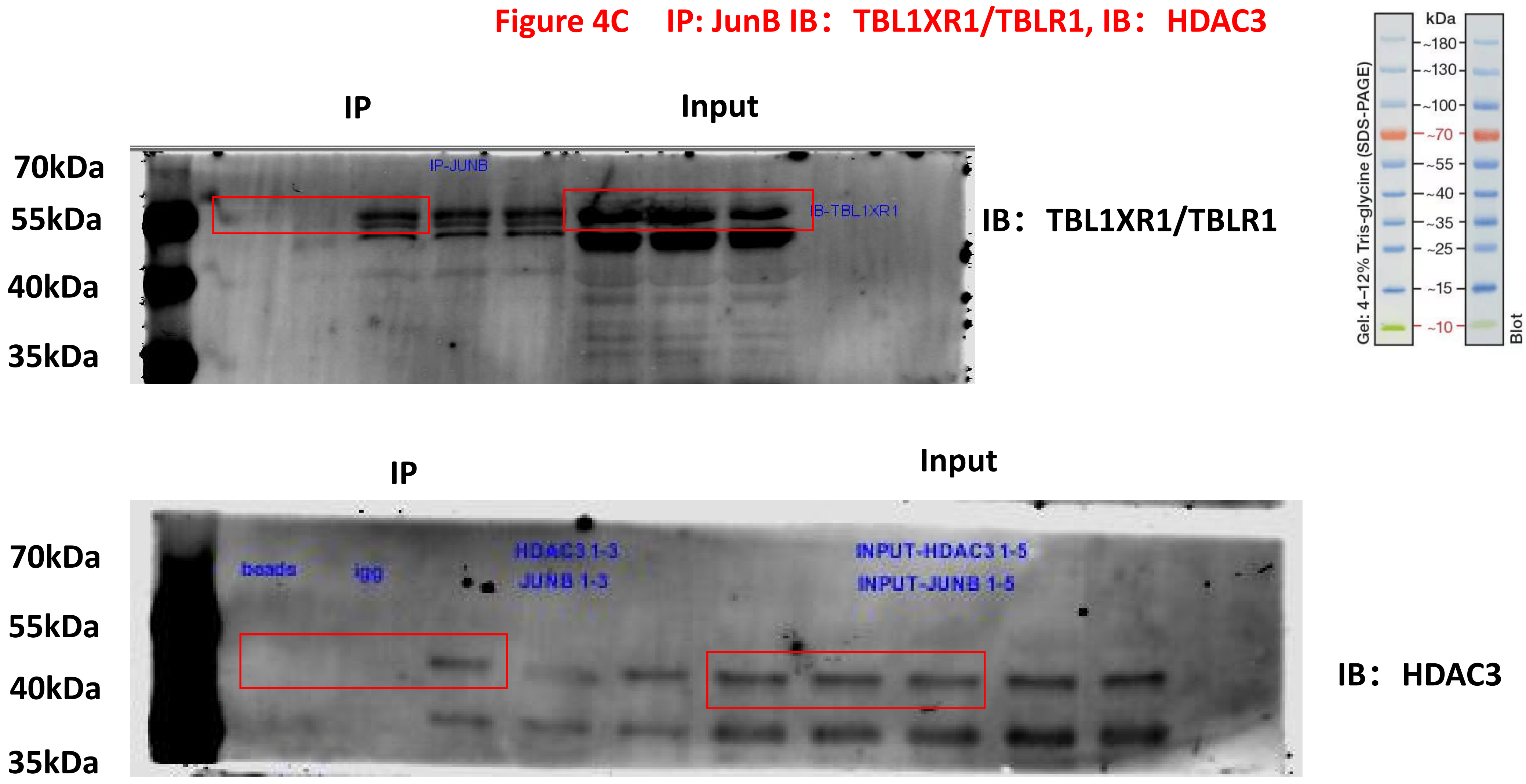

Figure 4C IP: Flag IB： NCOR2/SMRT

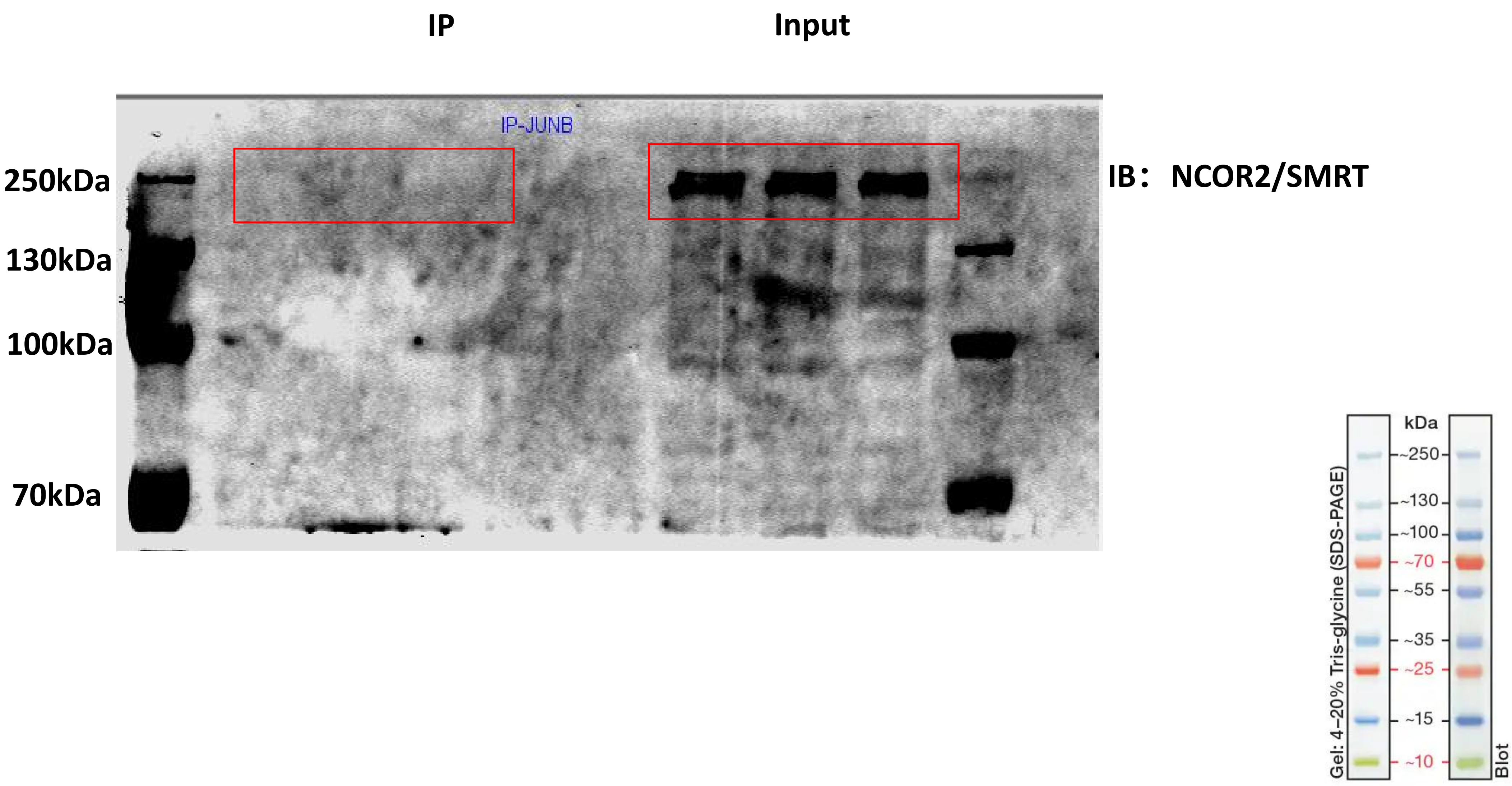

Figure 4D IP: TBL1XR1/TBLR1, IB： Flag-NCOR2-013, IB： TBL1XR1/TBLR1

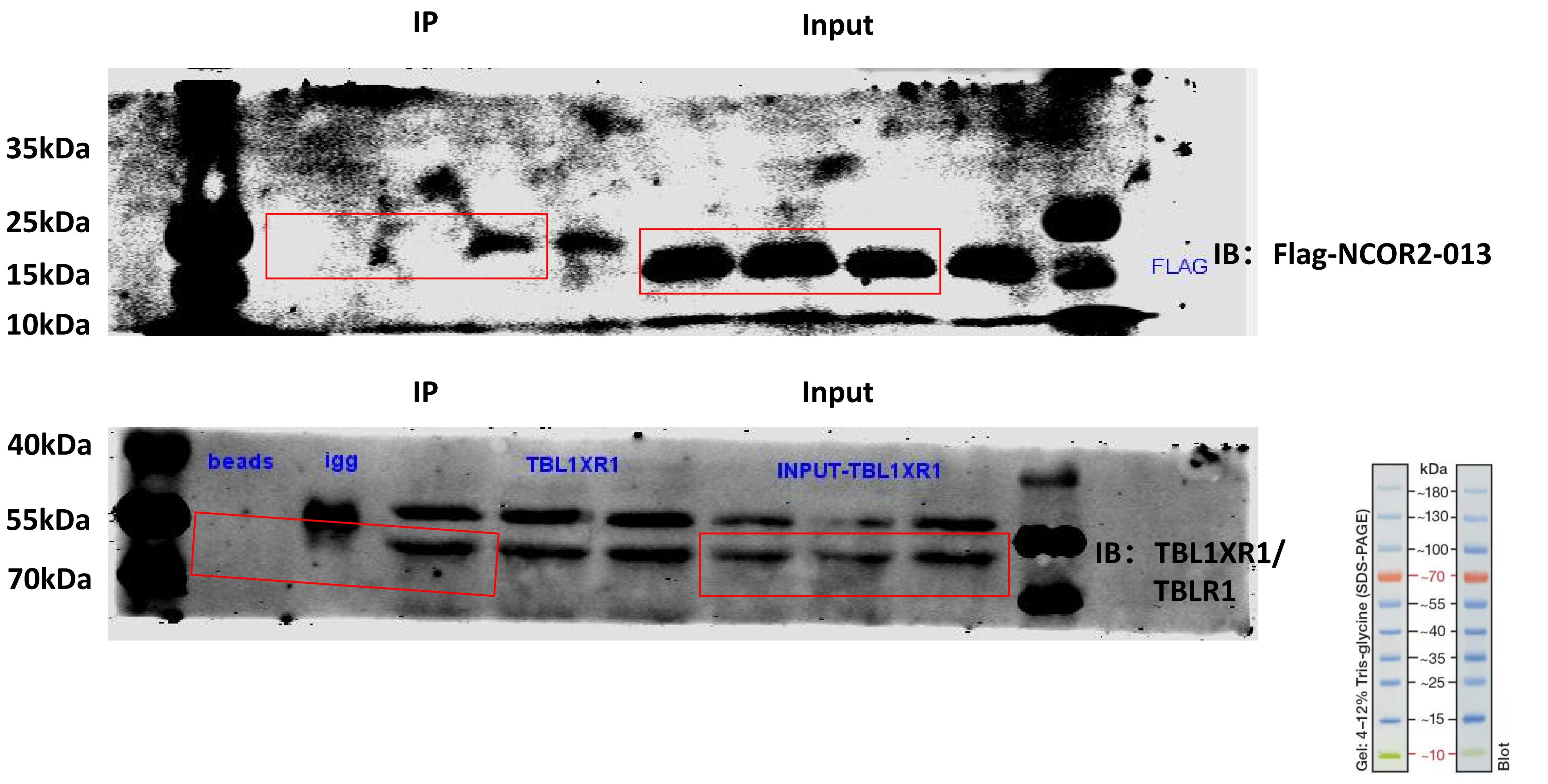

Figure 4D IP: TBL1XR1/TBLR1, IB： JunB, IB： HDAC3

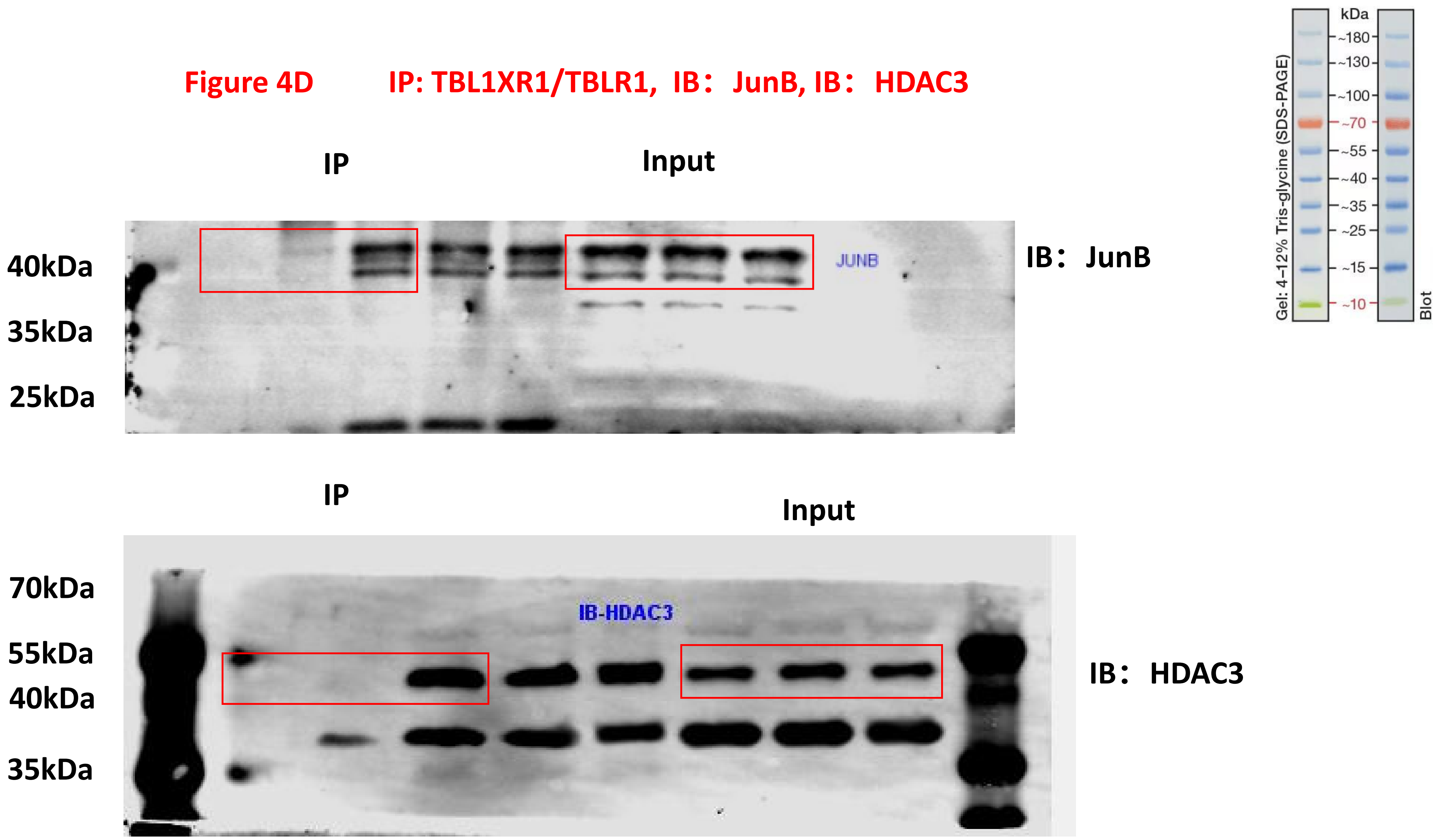

Figure 4D IP: TBL1XR1/TBLR1, IB： NCOR2/SMRT

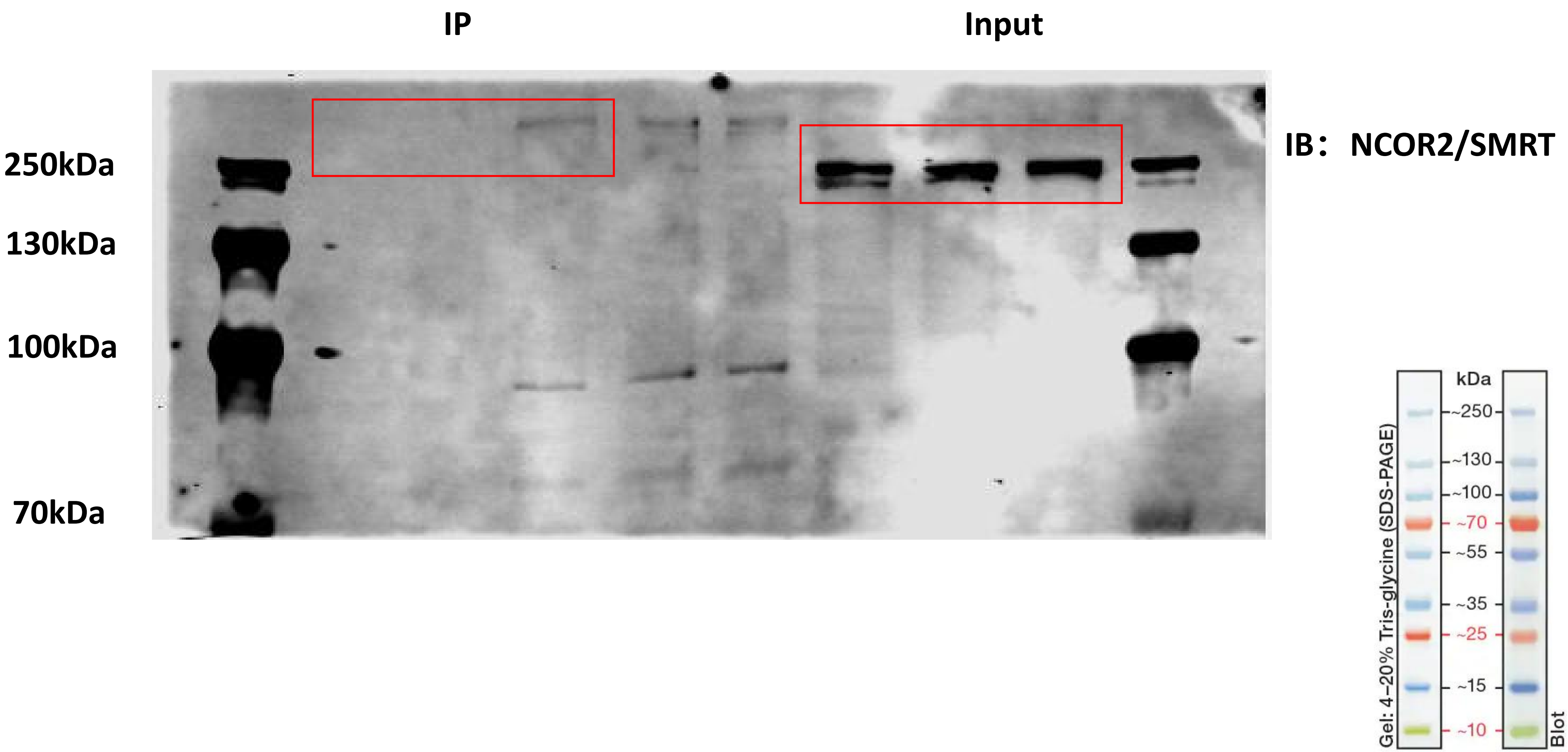

Figure 4E IP: HDAC3, IB： Flag-NCOR2-013, IB： HDAC3

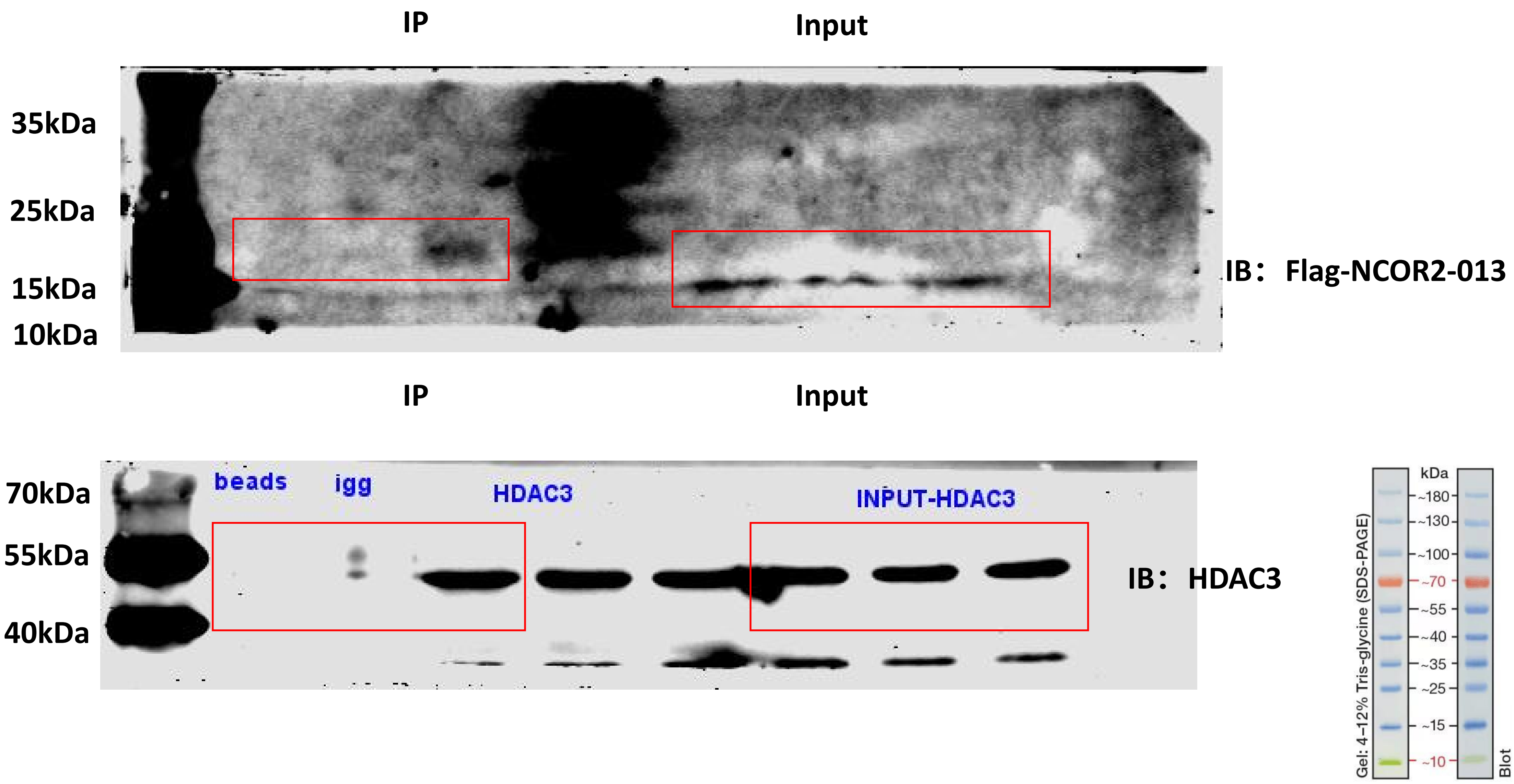

Figure 4E IP: HDAC3, IB： JunB, IB： TBL1XR1/TBLR1

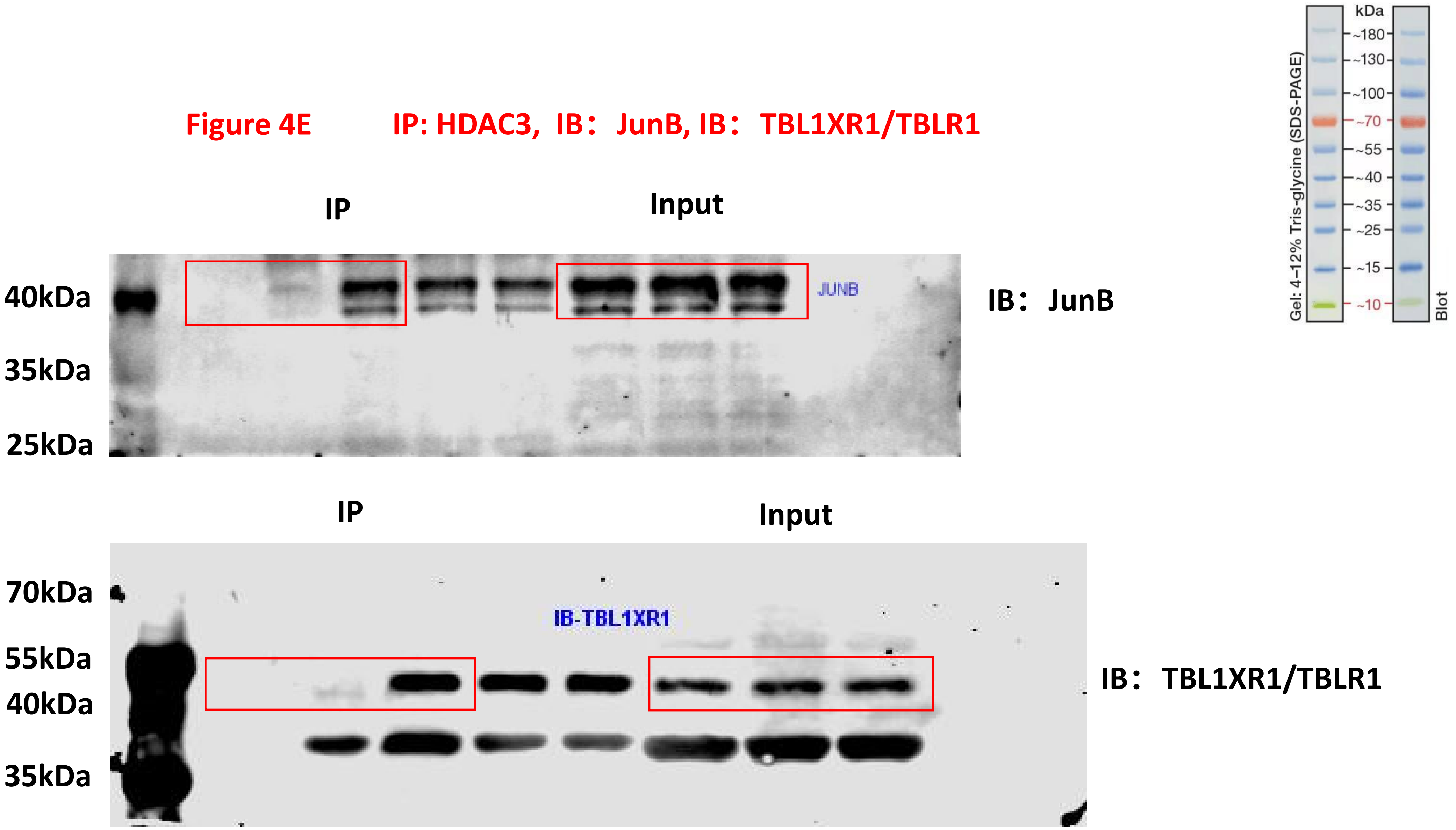

Figure 4E IP: HDAC3, IB： NCOR2/SMRT

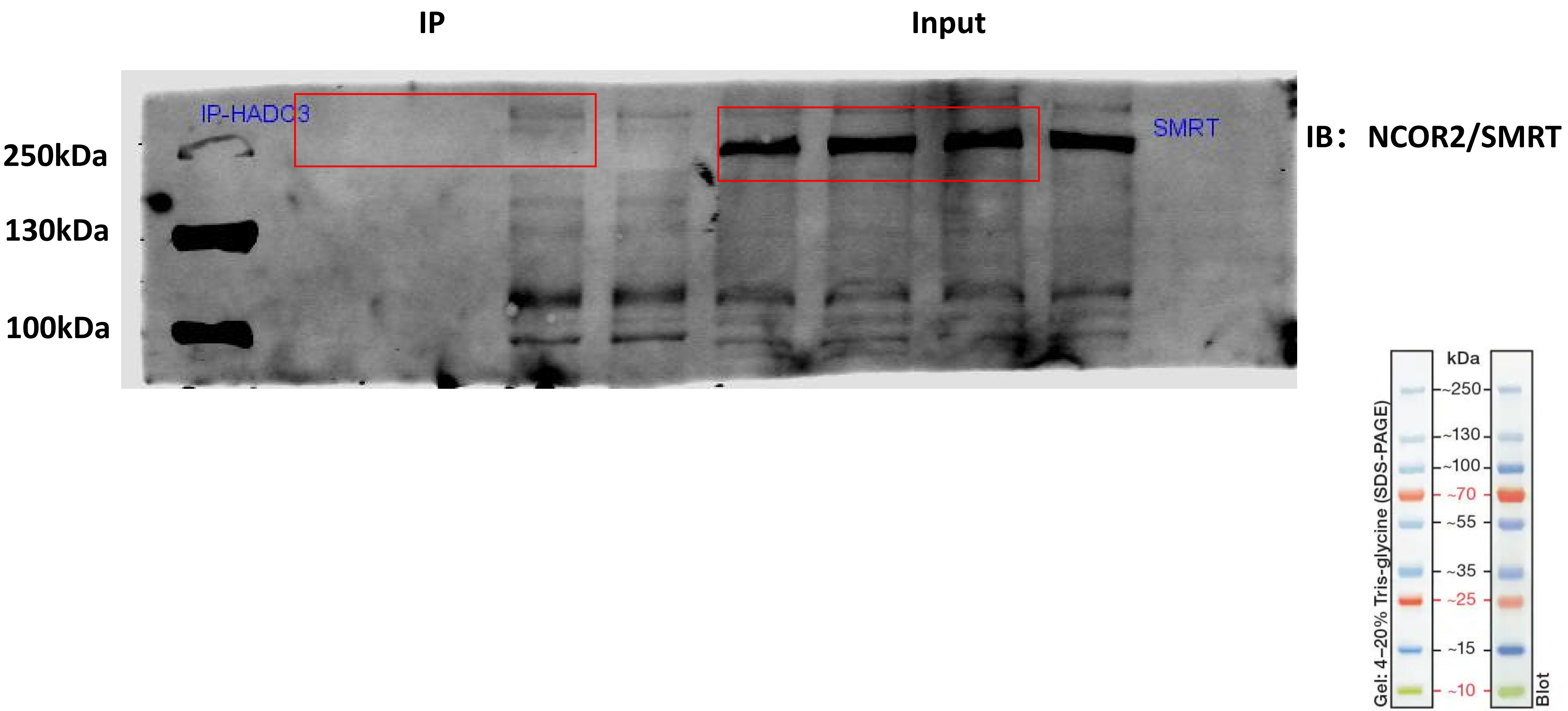

Figure 4F IP: Flag, IB： JunD， IB: Flag-NCOR2-013

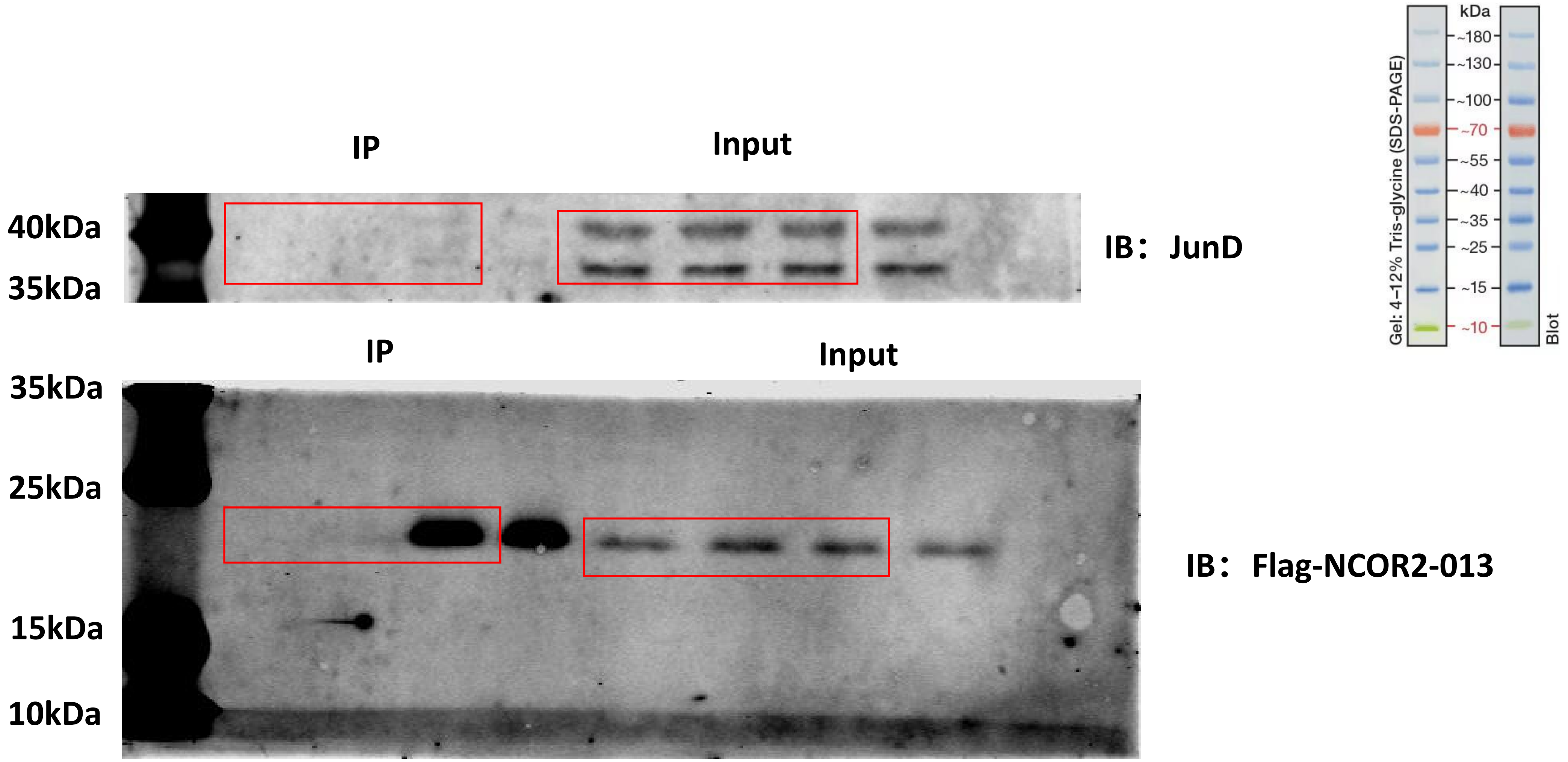

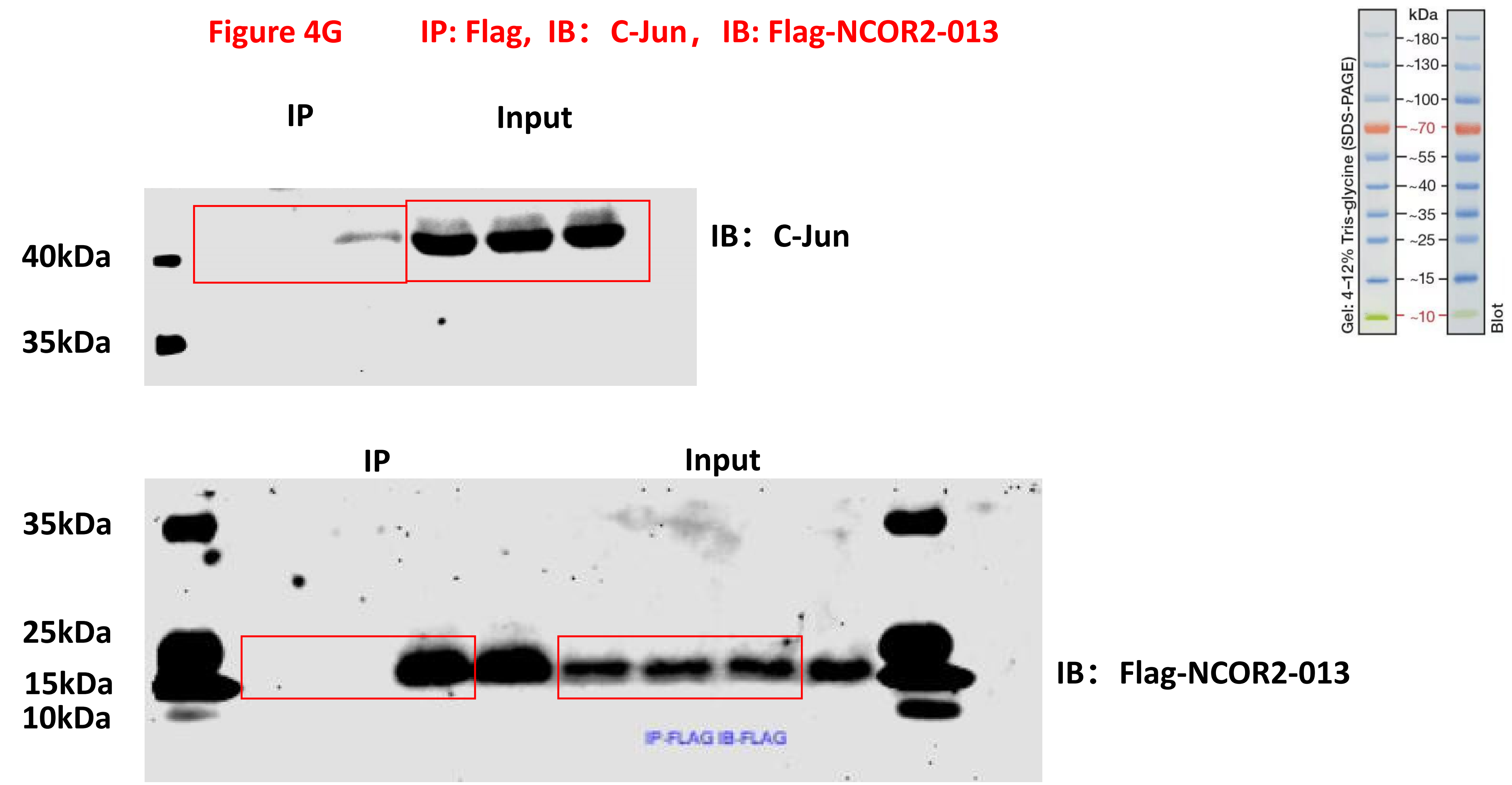

Figure 4G
IP: Flag，IB：C-Jun，IB: Flag-NCOR2-013
IP
Input
40kDa
35kDa
IB：C-Jun
IP
Input
35kDa
25kDa
15kDa
10kDa
IP-FLAG IB-FLAG
IB：Flag-NCOR2-013
kDa
~180
~130
~100
~70
~55
~40
~35
~25
~15
~10
Gel: 4–12% Tris-glycine (SDS-PAGE)
Blot

Figure 4H 24h

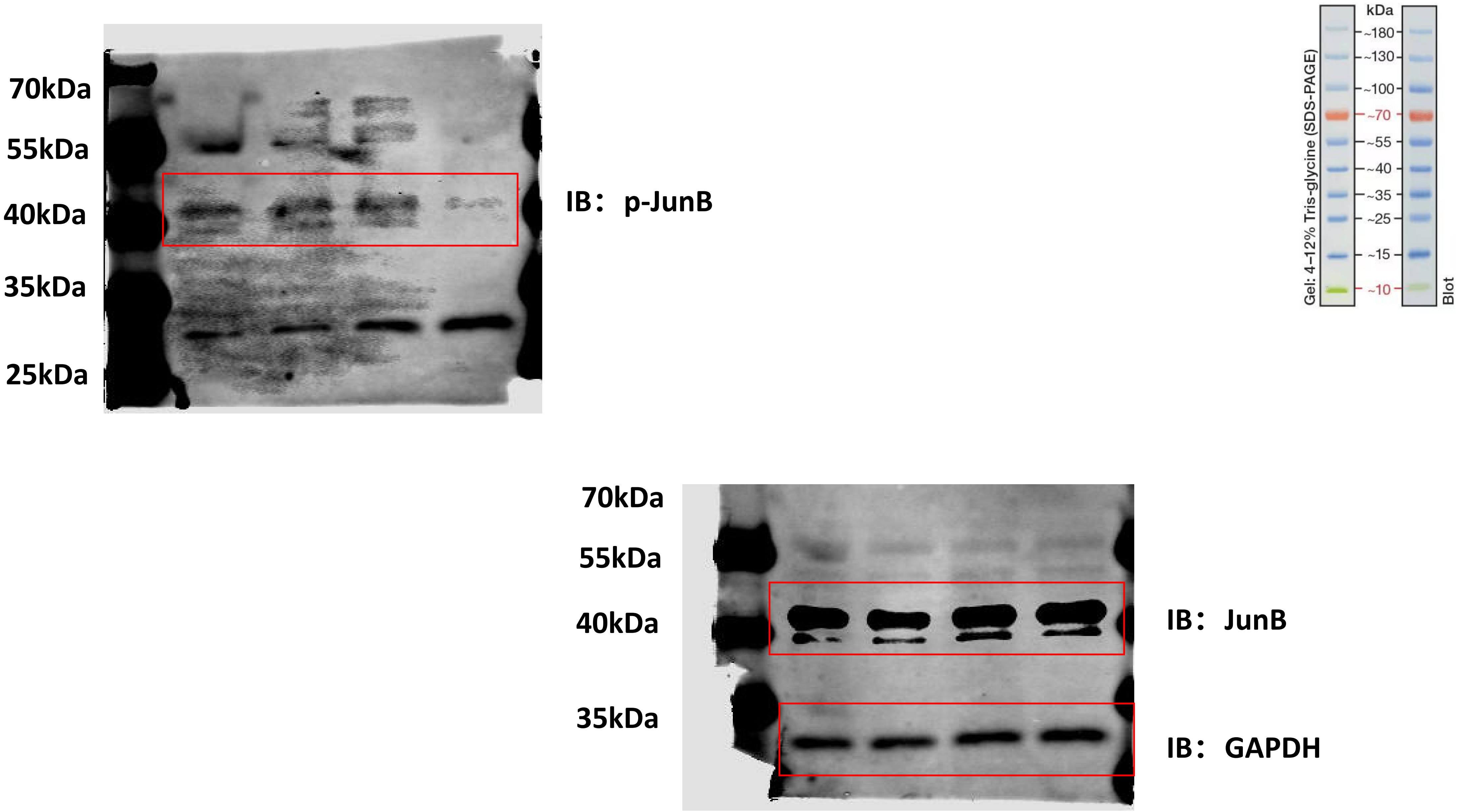

Figure 4H 48h

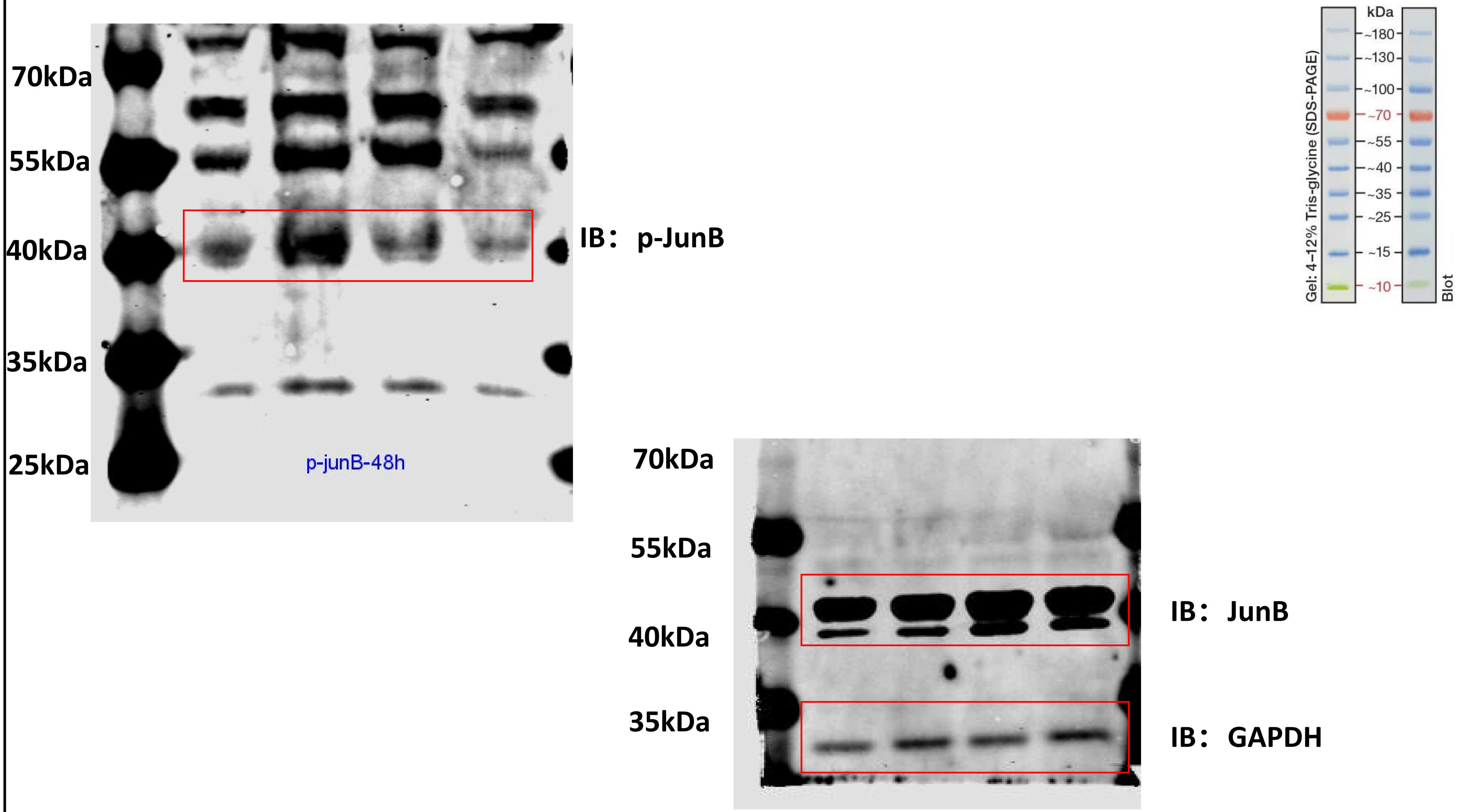

Figure 4I 24h

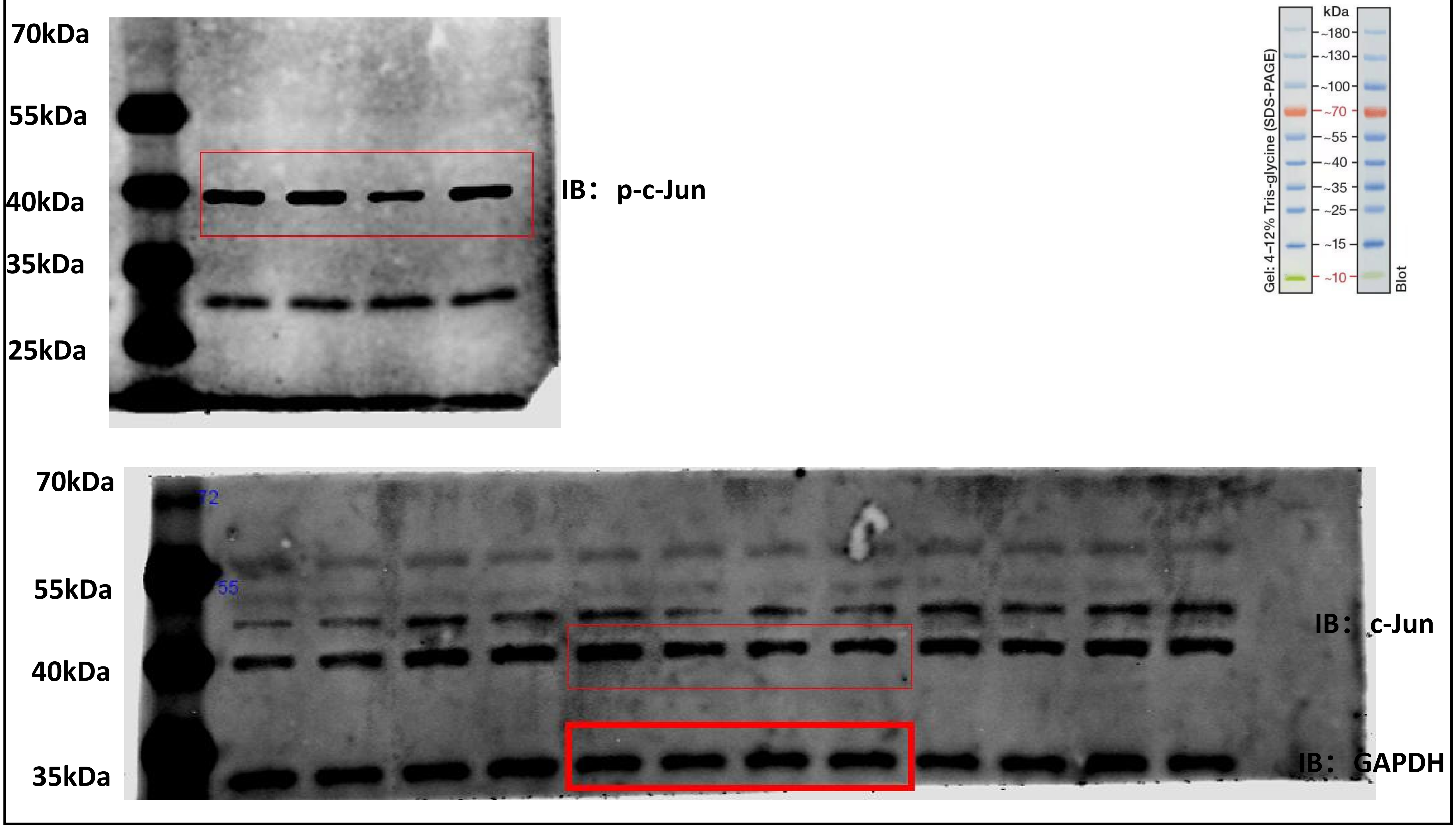

Figure 4I 48h

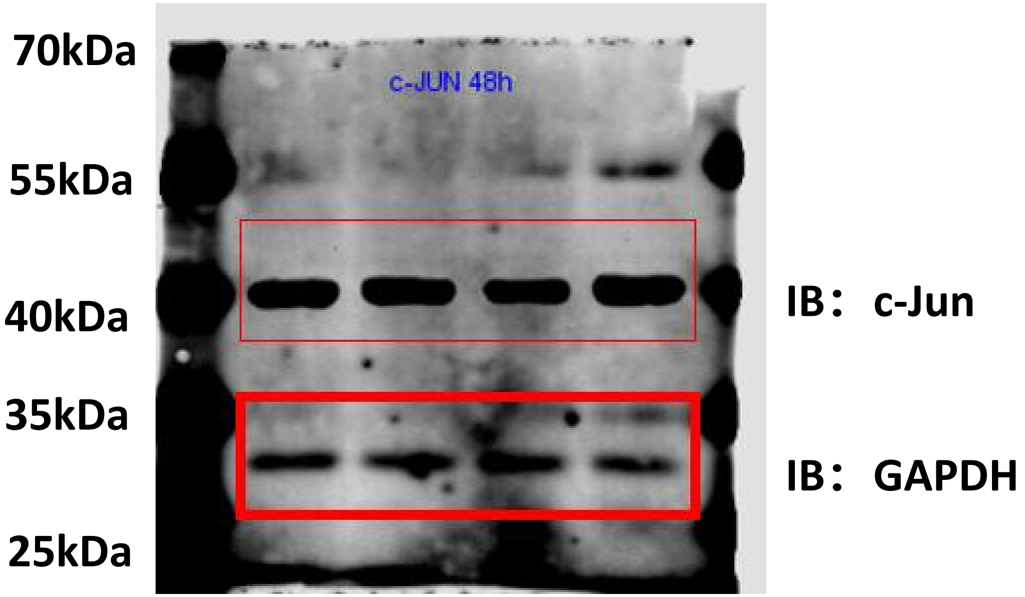

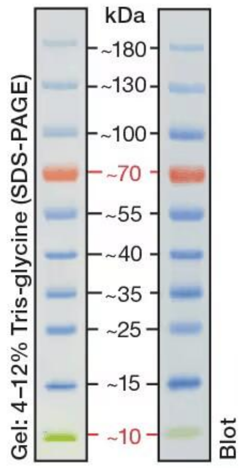

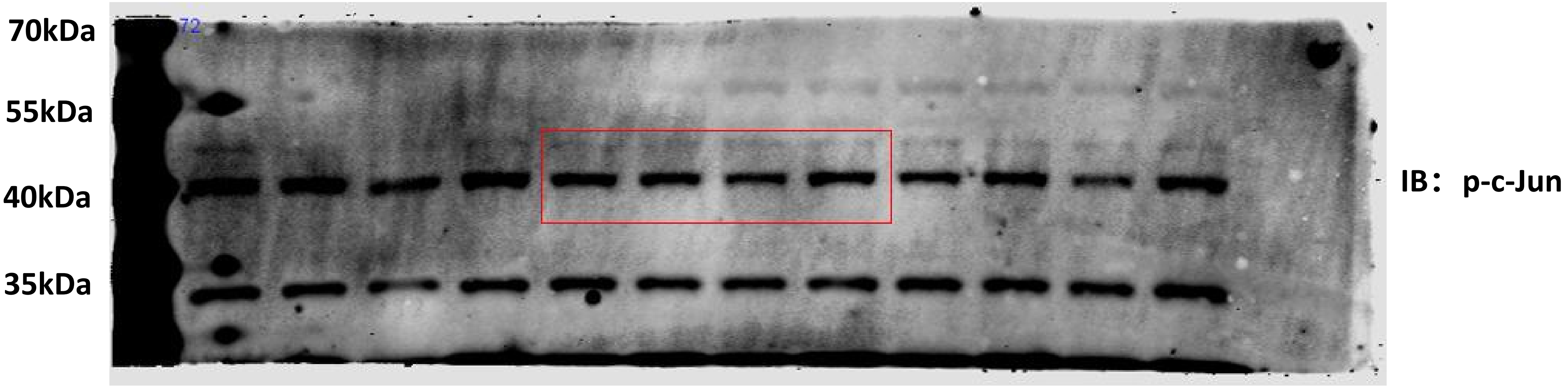

Figure 6A

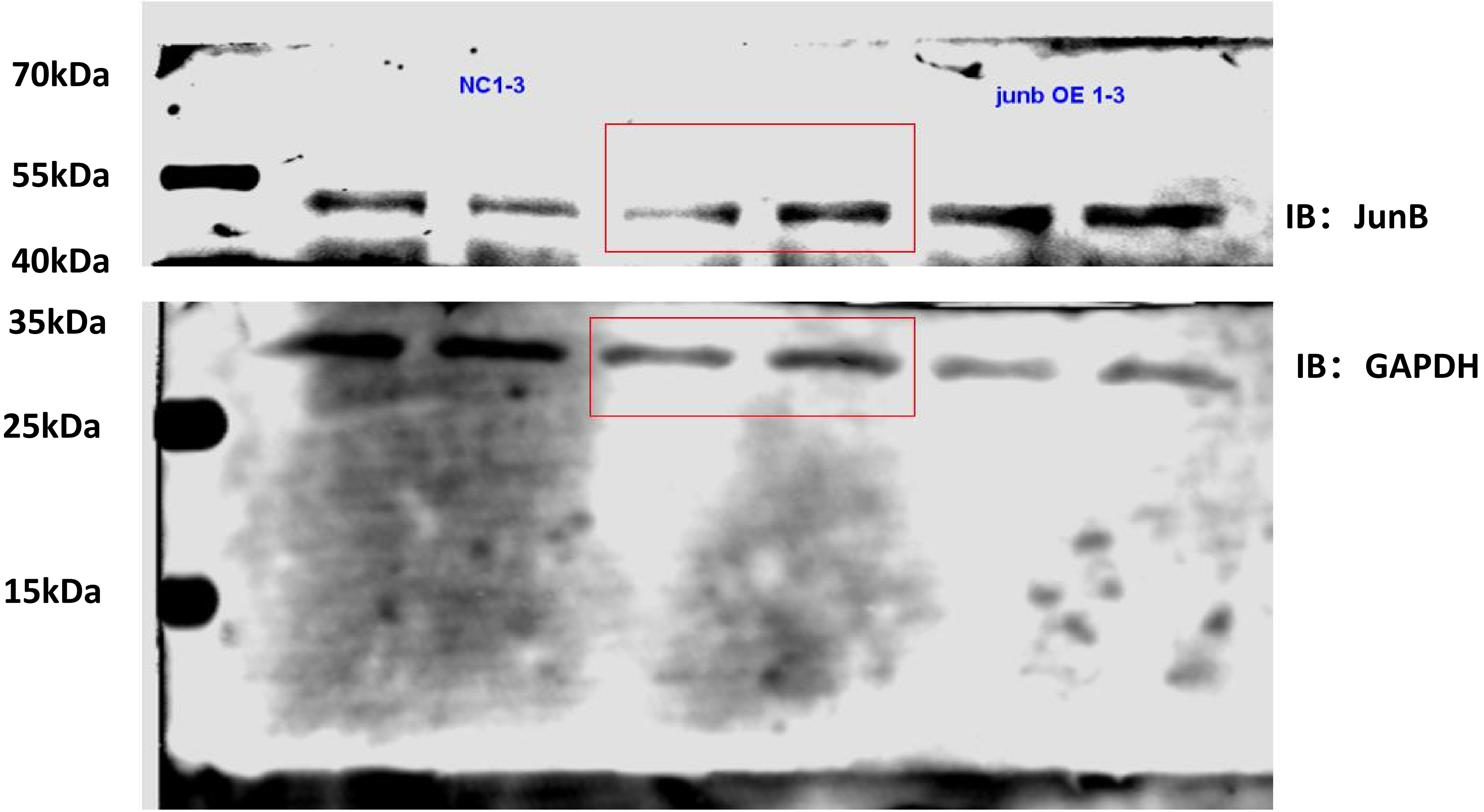

70kDa
55kDa
40kDa
35kDa
25kDa
15kDa
NC1-3
junb OE 1-3
IB：JunB
IB：GAPDH

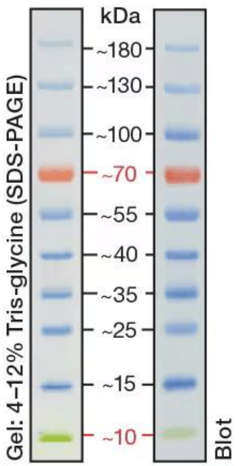

kDa
~180
~130
~100
~70
~55
~40
~35
~25
~15
~10
Gel: 4–12% Tris-glycine (SDS-PAGE)
Blot

Figure 7C

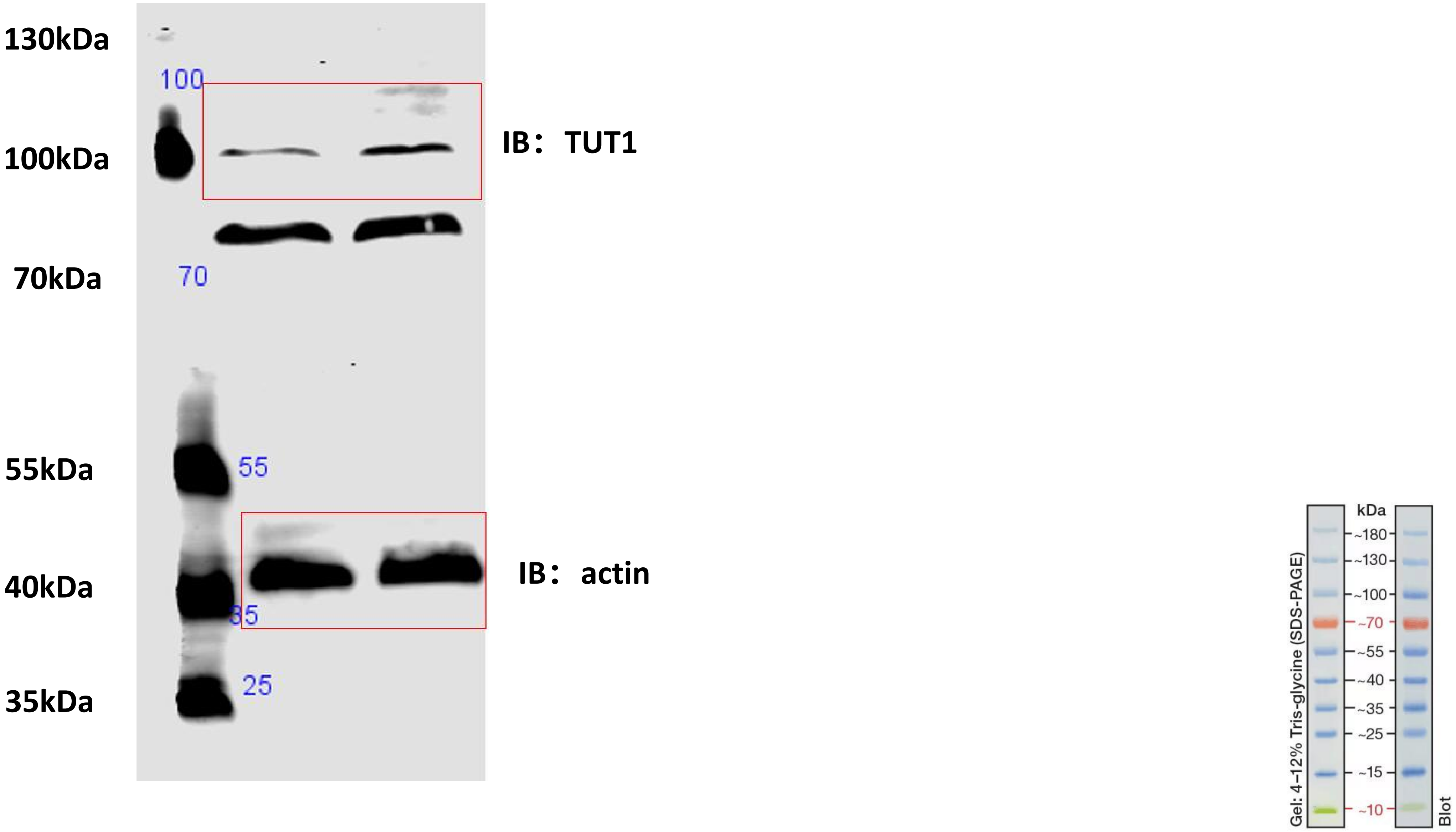
130kDa
100kDa
70kDa
55kDa
40kDa
35kDa
100
70
55
35
25
IB： TUT1
IB： actin
kDa
~180
~130
~100
~70
~55
~40
~35
~25
~15
~10
Gel: 4–12% Tris-glycine (SDS-PAGE)
Blot

Figure 7F

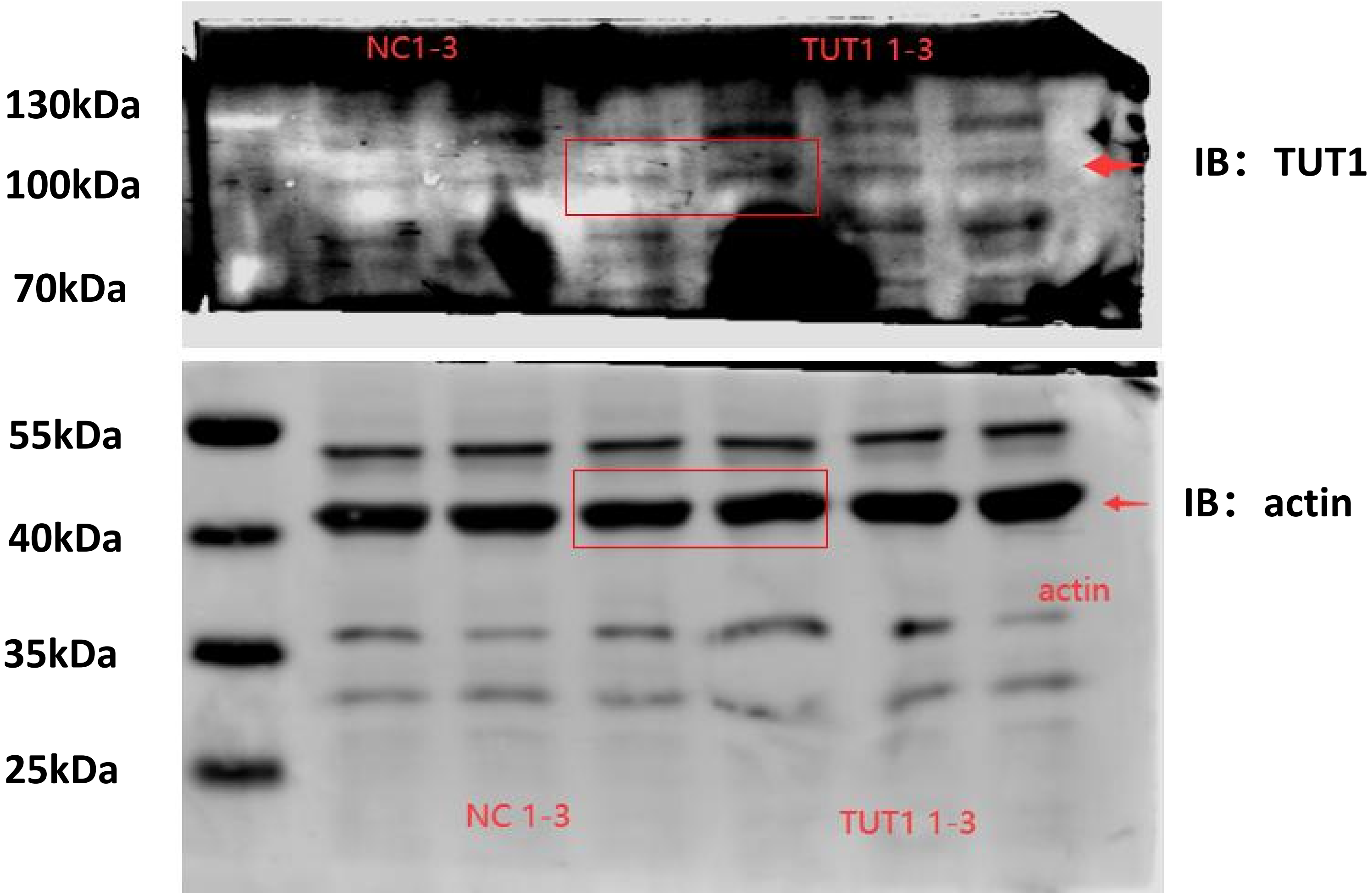

NC1-3
TUT1 1-3
130kDa
100kDa
70kDa
IB： TUT1
55kDa
40kDa
35kDa
25kDa
IB： actin
actin
NC 1-3
TUT1 1-3

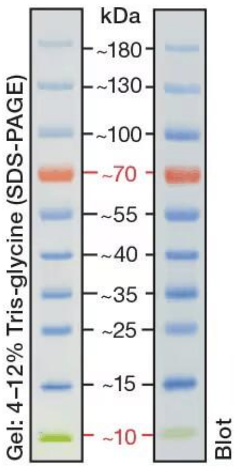

kDa
~180
~130
~100
~70
~55
~40
~35
~25
~15
~10
Gel: 4–12% Tris-glycine (SDS-PAGE)
Blot

Figure 7G

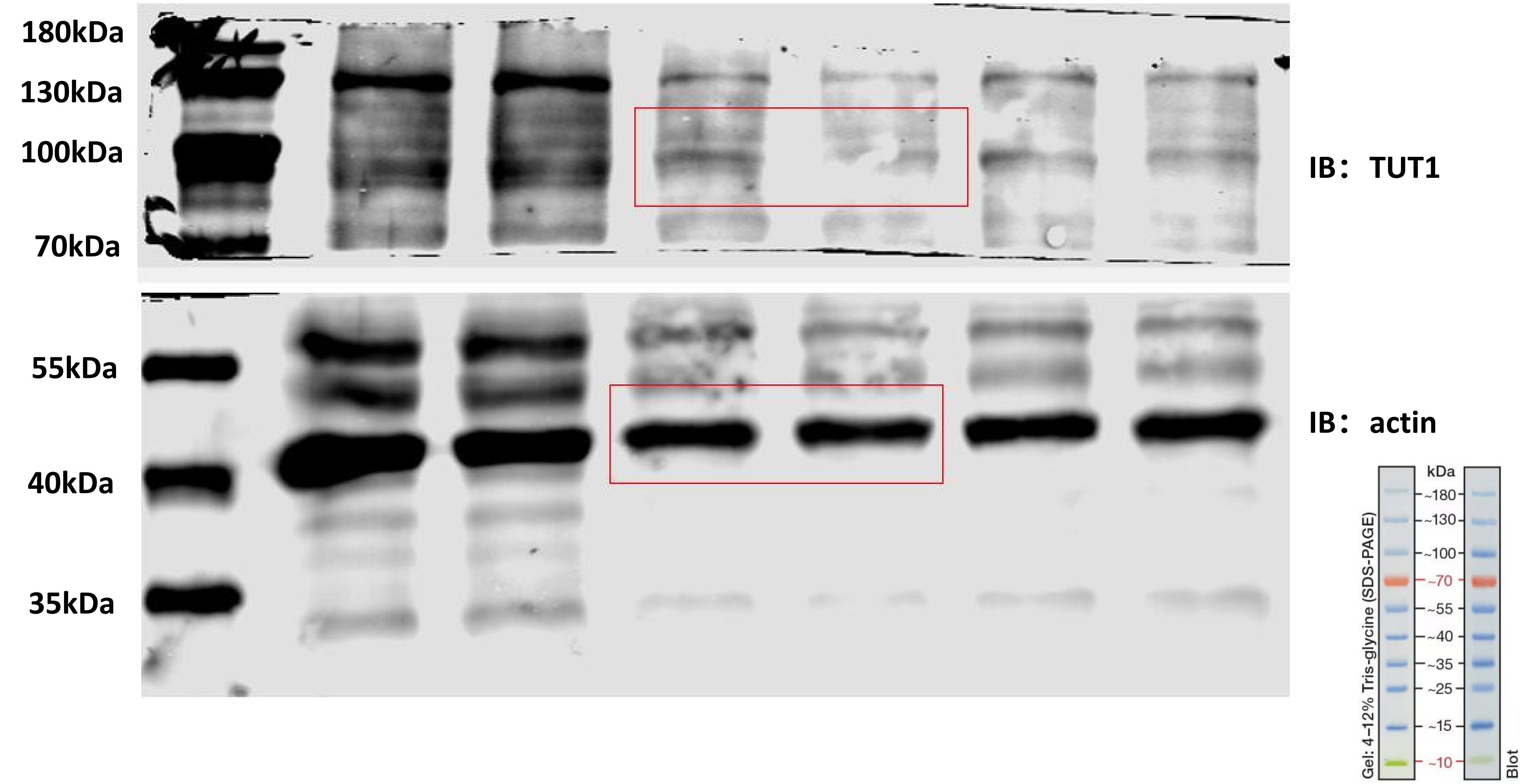
180kDa
130kDa
100kDa
70kDa
55kDa
40kDa
35kDa
IB： TUT1
IB： actin
kDa
~180
~130
~100
~70
~55
~40
~35
~25
~15
~10
Gel: 4–12% Tris-glycine (SDS-PAGE)
Blot

Supplementary Table 1. The summary of the truncated transcripts generated by ES-type AS event predicted by rMATs

Gene	Predicted transcript(s) matched the currently known transcript(s)	Matched currently known transcript(s)	Matched transcript(s) NOT detected in uninfected cells	Matched transcript(s) differentially highly expressed in infected cells (*padj*<0.05)
NR1H3	Yes	*NR1H3-028, 029, 031, 003, 023, 012, 032*	*NR1H3-029, 012,* yes; others, no	No
PPARA	Yes	*PPARA-002, 006, 007*	*PPARA-002,* yes; others, no	No
TLR6	Yes	*TLR6-002, 003*	Yes	No
IRF3	Yes	*IRF3-017, 009, 008, 012, 201, 023, 024, 202, 020*	*IRF3-023*, yes; others, no	*IRF3-020*, yes; others, no
MAP2K3	Yes	*MAP2K3-201, 016, 013*	Yes	No
NCOR2	Yes	*NCOR2-013*	Yes	Yes

The strategy for the screening of particular truncated transcript. (1) The truncated transcripts generated by ES-type AS event predicted by rMATs should be matched the currently known transcripts. (2) The truncated transcript is differentially highly expressed in *T. marneffei*-infected macrophages. (3) The truncated transcript can not be detected in uninfected cells.

Due to the expression in uninfected cells or/and insignificant difference in transcript expression, *NR1H3, PPARA, TLR6, IRF3 and MAP2K3* were excluded.

Supplementary Table 2. The differential expression analysis results of *NR1H3, PPARA, IRF3, MAP2K3* and *TLR6* transcripts in T. marneffei-infected THP-1 macrophages at 24 h post-infection.

transcript_id	transcript_name	Control1	Control2	Control3	*Tm*1	*Tm*2	*Tm*3	*pvalue*	*padj*
ENST00000440187	*NCOR2-013*	0	0	0	17.75560209	11.44595335	26.84260587	7.46E-06	0.00156278
ENST00000453428	*NCOR2-012*	0	0	0	0	0	0	NA	NA
ENST00000420698	*NCOR2-004*	0	0	0	5.07302917	3.5218318	0	0.3066841	NA
ENST00000356219	*NCOR2-201*	541.1004671	7563.025857	294.3280142	10154.51338	357.4659277	9554.050361	NA	NA
ENST00000429285	*NCOR2-001*	6080.09621	0	7615.977832	0	6804.179037	0	0.78642596	0.99998262
ENST00000397355	*NCOR2-202*	978.1431521	0	922.4201491	0	23.77236465	0	0.05290452	0.78650535
ENST00000405201	*NCOR2-005*	567.1149127	1.23136207	0	2227.059805	1686.076974	357.5818568	0.21311123	0.99998262
ENST00000404621	*NCOR2-002*	415.1905507	522.0975193	0.96185626	1051.808048	229.7995249	1950.882248	0.28105049	0.99998262
ENST00000404121	*NCOR2-203*	9.36520039	24.62724147	5.77113753	9.30055347	4.40228975	4.79332248	0.20918319	0.99998262
ENST00000458234	*NCOR2-003*	468.2600196	1.23136207	542.4869282	0	426.1416478	0	0.68561495	0.99998262
ENST00000542927	*NCOR2-024*	21.85213425	18.47043111	19.23712511	14.37358264	22.8919067	16.29729642	0.71939617	0.99998262
ENST00000443451	*NCOR2-022*	347.5529924	395.2672257	382.8187897	618.9095585	415.5761524	533.976124	0.05208474	0.78205829
ENST00000448008	*NCOR2-019*	41.62311286	38.17222429	30.77940018	34.66569931	86.28487909	35.47058633	0.36907157	0.99998262
ENST00000418829	*NCOR2-017*	2.08115564	0	15.38970009	29.59267015	9.68503745	9.58664495	0.31795707	0.99998262
ENST00000440337	*NCOR2-015*	54.11004671	64.03082783	91.37634428	100.6150785	46.66427135	40.26390881	0.77230653	0.99998262
ENST00000461081	*NCOR2-006*	118.6258716	156.3829834	75.02478794	214.7582348	61.63205649	65.18918569	0.96260225	0.99998262
ENST00000542565	*NCOR2-008*	19.77097861	75.1130865	14.42784383	149.6543605	15.8482431	126.5437134	0.1921929	0.99998262
ENST00000448614	*NCOR2-009*	1.04057782	0	26.93197516	0.84550486	6.16320565	1.91732899	0.39779333	0.99998262
ENST00000536195	*NCOR2-020*	65.55640275	39.40358636	19.23712511	27.05615556	11.44595335	54.64387624	0.61549353	0.99998262
ENST00000413172	*NCOR2-018*	11.44635604	0	8.6567063	43.96625279	9.68503745	13.42130294	0.1887402	0.99998262

transcript_id	transcript_name	Control1	Control2	Control3	*Tm*1	*Tm*2	*Tm*3	*pvalue*	*padj*
ENST00000494460	*NCOR2-007*	21.85213425	12.31362074	8.6567063	16.91009723	20.25053285	5.75198697	0.99158308	0.99998262
ENST00000474079	*NCOR2-014*	16.64924514	2.46272415	9.61856256	9.30055347	5.2827477	20.1319544	0.78911548	0.99998262
ENST00000493859	*NCOR2-010*	20.81155643	2.46272415	5.77113753	11.83706806	12.3264113	29.71859936	0.37539192	0.99998262
ENST00000473999	*NCOR2-011*	41.62311286	33.24677599	63.48251287	81.16846669	54.5883929	64.23052119	0.2045823	0.99998262
ENST00000464377	*NCOR2-016*	12.48693386	13.54498281	10.58041881	15.2190875	9.68503745	43.13990229	0.24919321	0.99998262
ENST00000495866	*NR1H3-026*	0	0	0	0	0	0	NA	NA
ENST00000529540	*NR1H3-029*	0	0	0	0	0	0	NA	NA
ENST00000395397	*NR1H3-031*	0	307.8405184	26.93197516	25.36514584	0	47.93322477	0.39153591	0.99998262
ENST00000405576	*NR1H3-003*	0	40.63494843	22.12269388	24.51964098	0	0	0.6631147	0.99998262
ENST00000531660	*NR1H3-037*	0	0	19.23712511	11.83706806	0	0	0.85208738	0.99998262
ENST00000532630	*NR1H3-036*	0	0	0	0	0	0	NA	NA
ENST00000407404	*NR1H3-002*	0	0	0	0	0	0	NA	NA
ENST00000444396	*NR1H3-010*	0	16.00770696	0	0	0	24.92527688	0.86423579	0.99998262
ENST00000457932	*NR1H3-011*	0	0	0	0	0	0	NA	NA
ENST00000412937	*NR1H3-012*	0	0	0	0	0	0	NA	NA
ENST00000449369	*NR1H3-013*	0	0	0	0	0	0	NA	NA
ENST00000486991	*NR1H3-009*	0	0	0	0	0	49.85055376	0.1020103	0.93455796
ENST00000437276	*NR1H3-017*	0	0	0	0	0	0	NA	NA
ENST00000436029	*NR1H3-016*	0	0	0	0	0	0	NA	NA
ENST00000476086	*NR1H3-038*	0	0	26.93197516	19.44661181	0	11.50397395	0.94870555	0.99998262
ENST00000530310	*NR1H3-035*	0	0	0	6.76403889	9.68503745	0	0.17488353	0.99815157

transcript_id	transcript_name	Control1	Control2	Control3	*Tm*1	*Tm*2	*Tm*3	*pvalue*	*padj*
ENST00000441012	*NR1H3-030*	218.5213425	0	196.2186761	188.5475841	180.4938797	407.4324106	0.5918476	0.99998262
ENST00000467728	*NR1H3-001*	182.1011187	124.3675694	172.1722698	239.2778758	169.9283843	71.89983716	0.98939671	0.99998262
ENST00000405853	*NR1H3-006*	39.54195721	0	24.04640639	27.90166042	31.6964862	0	0.96615561	0.99998262
ENST00000481020	*NR1H3-005*	3.12173346	25.85860355	17.3134126	19.44661181	6.16320565	8.62798046	0.66398459	0.99998262
ENST00000481889	*NR1H3-023*	1.04057782	30.78405184	13.46598758	6.76403889	0.88045795	39.30524431	0.97353898	0.99998262
ENST00000527464	*NR1H3-028*	9.36520039	0	6.73299379	0.84550486	3.5218318	0	0.34026224	0.99998262
ENST00000527949	*NR1H3-032*	30.17675682	32.01541392	15.38970009	27.90166042	53.70793495	15.33863193	0.63267705	0.99998262
ENST00000436778	*NR1H3-007*	7.28404475	7.38817244	1.92371251	8.45504861	5.2827477	8.62798046	0.57155624	0.99998262
ENST00000420369	*NR1H3-019*	78.0433366	11.08225866	72.13921917	75.24993266	54.5883929	0	0.8529354	0.99998262
ENST00000419652	*NR1H3-014*	31.21733464	44.32903465	27.89383141	27.90166042	20.25053285	15.33863193	0.12523099	0.96447689
ENST00000461778	*NR1H3-015*	2.08115564	0	4.80928128	1.69100972	0.88045795	0	0.45726338	NA
ENST00000525441	*NR1H3-039*	13.52751168	0	9.61856256	6.76403889	17.609159	8.62798046	0.69163111	0.99998262
ENST00000473222	*NR1H3-022*	0	0	0	0	0	0	NA	NA
ENST00000483882	*NR1H3-021*	32.25791246	61.56810369	81.75778173	74.4044278	51.0665611	29.71859936	0.76830051	0.99998262
ENST00000462051	*NR1H3-024*	24.97386771	4.92544829	0.96185626	21.98312639	16.72870105	21.0906189	0.40743449	0.99998262
ENST00000498548	*NR1H3-018*	13.52751168	16.00770696	13.46598758	9.30055347	10.5654954	9.58664495	0.26107015	0.99998262
ENST00000487913	*NR1H3-008*	15.60866732	34.47813806	38.47425022	36.35670904	21.1309908	16.29729642	0.67049247	0.99998262
ENST00000494018	*NR1H3-025*	1.04057782	8.61953452	0.96185626	0.84550486	0.88045795	0.9586645	0.2055638	NA
ENST00000262735	*PPARA-201*	0	0	0	0	0	0	NA	NA
ENST00000460086	*PPARA-004*	0	0	0	0	0	0	NA	NA
ENST00000396000	*PPARA-202*	75.96218096	9.85089659	33.66496895	7.60954375	16.72870105	130.3783714	0.77610553	0.99998262

**transcript_id**	**transcript_name**	**Control1**	**Control2**	**Control3**	***Tm*1**	***Tm*2**	***Tm*3**	***pvalue***	***padj***
ENST00000407236	*PPARA-001*	3.12173346	588.5910712	229.8836451	1713.838354	1075.039157	0	0.44855789	0.99998262
ENST00000402126	*PPARA-008*	10.40577821	27.08996562	35.58868146	36.35670904	27.29419645	86.27980459	0.17561615	0.99906307
ENST00000493286	*PPARA-005*	142.5591615	169.9279662	166.4011322	74.4044278	49.3056452	162.0142997	0.1895055	0.99998262
ENST00000434345	*PPARA-203*	112.3824047	119.4421212	202.9516699	0	71.31709394	0	0.25866364	0.99998262
ENST00000481567	*PPARA-006*	4.16231129	12.31362074	4.80928128	10.14605834	10.5654954	3.83465798	0.77895214	0.99998262
ENST00000415785	*PPARA-009*	13.52751168	0	5.77113753	0	0	0	0.12759294	0.96684162
ENST00000420804	*PPARA-003*	10.40577821	6.15681037	3.84742502	4.22752431	7.0436636	0.9586645	0.45941472	0.99998262
ENST00000440343	*PPARA-002*	0	0	0	0	0	0	NA	NA
ENST00000496865	*PPARA-010*	34.33906811	22.16451733	22.12269388	19.44661181	26.4137385	32.59459284	0.98407133	0.99998262
ENST00000484619	*PPARA-007*	5.20288911	0	4.80928128	0	0	10.54530945	0.98219648	0.99998262
ENST00000508254	*TLR6-002*	0	44.32903465	17.3134126	8.455048613	7.924121549	14.37996743	0.506220585	0.999982619
ENST00000514655	*TLR6-003*	88.44911482	50.48584502	202.9516699	5.918534029	19.3700749	29.71859936	0.002360778	0.146457759
ENST00000436693	*TLR6-201*	3953.155143	3221.243185	2689.350091	5714.767358	6466.083184	6560.141142	8.24E-05	0.011919942
ENST00000381950	*TLR6-001*	514.0454438	4361.484465	3498.271202	294.2356917	353.9440959	699.8250817	0.003398385	0.186589606
ENST00000483928	*MAP2K3-007*	0	25.7803813	15.4225221	12.70700861	36.17402188	11.53819082	0.698221361	0.999947878
ENST00000496046	*MAP2K3-004*	0	0	0	0	0	0	NA	NA
ENST00000526076	*MAP2K3-016*	0	0	0	0	0	0	NA	NA
ENST00000361818	*MAP2K3-201*	0	0	0	0	0	0	NA	NA
ENST00000316920	*MAP2K3-013*	0	0	0	5.929937349	3.529172867	0	0.264985751	NA
ENST00000395491	*MAP2K3-002*	2733.46814	200.1048644	3350.542927	88.94906024	56.46676587	3452.803602	0.633274201	0.999947878
ENST00000342679	*MAP2K3-001*	510.9674486	3185.718547	345.0789321	4043.370138	4075.312368	458.643085	0.368618072	0.999947878

**transcript_id**	**transcript_name**	**Control1**	**Control2**	**Control3**	***Tm*1**	***Tm*2**	***Tm*3**	***pvalue***	***padj***
ENST00000583508	*MAP2K3-017*	104.8937242	14.73164646	234.2295545	73.70064991	15.8812779	0	0.266877176	0.999947878
ENST00000479129	*MAP2K3-006*	1.038551725	0	41.44802816	166.8853797	0	24.99941344	0.412020888	0.999947878
ENST00000529517	*MAP2K3-011*	17.65537932	11.04873484	7.711261052	0	0	11.53819082	0.392564918	0.999947878
ENST00000477540	*MAP2K3-009*	140.2044828	52.78839981	84.82387158	212.6306107	63.5251116	80.76733573	0.617352873	0.999947878
ENST00000534743	*MAP2K3-010*	24.92524139	12.27637205	26.02550605	52.52230223	39.70319475	29.80699295	0.066270357	0.852310358
ENST00000527123	*MAP2K3-014*	22.84813795	18.41455807	11.56689158	16.09554423	37.93860832	18.26880213	0.459534279	0.999947878
ENST00000596822	*IRF3-017*	0	44.19493938	0	65.22931084	54.70217943	48.07579508	0.372383814	0.999947878
ENST00000598108	*IRF3-028*	0	0	0	0	0	0	NA	NA
ENST00000595034	*IRF3-023*	0	0	0	0	0	0	NA	NA
ENST00000593337	*IRF3-027*	0	0	0	0	10.5875186	0	0.289332567	NA
ENST00000598808	*IRF3-010*	638.7093107	2032.967211	374.9600687	241.4331635	2105.151615	294.2238659	0.857626689	0.999947878
ENST00000377139	*IRF3-001*	341.6835175	55.24367422	252.5437995	160.9554423	96.16996062	472.1043076	0.863052384	0.999947878
ENST00000601291	*IRF3-002*	219.1344139	94.52806478	138.8026989	462.5351132	0	360.5684631	0.636023397	0.999947878
ENST00000593922	*IRF3-011*	96.5853104	14.73164646	0	154.1783711	127.9325164	343.2611768	0.134448832	0.976872822
ENST00000597198	*IRF3-004*	2.07710345	0	0	6.777071256	0	0	0.662573211	NA
ENST00000597636	*IRF3-003*	894.193035	310.5922129	598.5866392	881.8663972	161.4596587	798.0581983	0.971115948	0.999947878
ENST00000599223	*IRF3-005*	244.0596553	0	158.0808516	279.5541893	0	179.8034736	0.940869351	0.999947878
ENST00000309877	*IRF3-006*	252.3680691	29.46329292	169.6477432	219.4076819	34.40943545	418.2594172	0.617524806	0.999947878
ENST00000596756	*IRF3-025*	10.38551725	0	0	16.09554423	0	0	0.866862799	0.999947878
ENST00000600022	*IRF3-012*	119.4334483	0	149.4056829	91.49046196	0	219.2256255	0.934158483	0.999947878
ENST00000599144	*IRF3-009*	147.4743449	73.6582323	31.80895184	501.5032729	0	0	0.717627621	0.999947878

**transcript_id**	**transcript_name**	**Control1**	**Control2**	**Control3**	***Tm*1**	***Tm*2**	***Tm*3**	***pvalue***	***padj***
ENST00000377135	*IRF3-201*	45.69627589	0	17.35033737	104.1974706	0	72.11369261	0.526132551	0.999947878
ENST00000600911	*IRF3-007*	76.85282763	45.42257658	0	165.1911119	0	0	0.898683083	0.999947878
ENST00000593818	*IRF3-022*	13.50117242	0	17.35033737	0	28.23338293	0	0.966059713	0.999947878
ENST00000601373	*IRF3-024*	25.96379312	33.14620453	254.4716147	91.49046196	23.82191685	67.30611311	0.485902207	0.999947878
ENST00000601809	*IRF3-019*	24.92524139	46.65021379	28.91722895	33.88535628	37.93860832	44.22973147	0.609286321	0.999947878
ENST00000596765	*IRF3-008*	16.6168276	0	10.60298395	74.54778382	0	0	0.647312123	0.999947878
ENST00000594387	*IRF3-013*	127.7418621	119.0808089	113.7411005	155.025505	175.5763501	153.8425442	0.034333565	0.672167551
ENST00000595240	*IRF3-016*	8.308413798	8.593460435	8.675168684	18.63694595	26.4687965	12.49970672	0.040389643	0.71721687
ENST00000596788	*IRF3-026*	3.115655174	3.682911615	8.675168684	7.624205163	6.176052517	5.769095409	0.677700892	0.999947878
ENST00000442265	*IRF3-202*	41.54206899	0	3.855630526	0	0	0	0.057026029	0.812591336
ENST00000600453	*IRF3-020*	39.46496554	25.7803813	54.942735	0	0	0	1.04E-08	4.31E-06
ENST00000599680	*IRF3-021*	24.92524139	19.64219528	0.963907632	1.694267814	30.88026258	19.23031803	0.893960369	0.999947878
ENST00000597180	*IRF3-029*	6.231310349	14.73164646	15.4225221	20.33121377	10.5875186	20.19183393	0.437751422	0.999947878
ENST00000597369	*IRF3-014*	207.710345	114.1702601	181.2146347	132.1528895	132.3439825	112.4973605	0.235294529	0.999947878
ENST00000602190	*IRF3-018*	12.4626207	17.18692087	0.963907632	17.78981205	15.8812779	28.84547705	0.300013039	0.999947878
ENST00000596644	*IRF3-015*	11.42406897	14.73164646	17.35033737	30.49682065	0.882293217	24.03789754	0.763881833	0.999947878

Supplementary Table 3. RT-qPCR primers sequences for the study.

TNF-α	Forward	GAGGCCAAGCCCTGGTATG
	Reverse	CGGGCCGATTGATCTCAGC
IL-1β	Forward	ATGATGGCTTATTACAGTGGCAA
	Reverse	GTCGGAGATTCGTAGCTGGA
TUT1	Forward	TCACTTCCTATCGGATCGGC
	Reverse	CTGTACCGACAAAAACACAAGC
JunB	Forward	ACGACTCATACACAGCTACGG
	Reverse	GCTCGGTTTCAGGAGTTTGTAGT

Supplementary Table 4. RT-qPCR primers sequences for *NCOR2-013.*

NCOR2-013 **-exon 10 to 11**	Forward	TGCTCTGCTCTGTACCAGGTCTC
	Reverse	ACAAAACCAACCACCACGTCCTC
NCOR2-013 **-exon 12**	Forward	AGGTAGGCAAGGCGGTCCATG
	Reverse	GCATCATCGACCTGTCCCAAGTG

Supplementary Table 5. ChIP-qPCR primers sequences for the study.

TNF-α-1 (chr6: 31574664-31574737)	Forward	GTCCAGGGCTATGGAAGTCG
	Reverse	GGTCCTGGAGGCTCTTTCAC
TNF-α-2 (chr6: 31575099-31575278)	Forward	CAACCCCGTTTTCTCTCCCTCAAG
	Reverse	GGCCACTGACTGATTTGTGTGTAGG
TNF-α-3 (chr6: 31575339-31575412)	Forward	GGGAGTGTGAGGGGTATCCT
	Reverse	CTGCACCTTCTGTCTCGGTT
IL-1β-1 (chr2: 112836446-112836756)	Forward	CTGGAGCAGAGGCTTTGACA
	Reverse	AACCTCTTCGAGGCACAAGG
IL-1β-2 (chr2: 112837433-112837605)	Forward	CAGGTGCTGTTACCCTAGCC
	Reverse	ACTTTGCTGGTGTCTCGGTT
IL-1β-3 (chr2: 112838165-112838491)	Forward	GTTTGCTGCTTGAATGGGTGAATGG
	Reverse	AGGAGGGCTCAGTGTTAGGAATGG
