# Supplementary material for: Talaromyces marneffei suppresses macrophage inflammation by regulating host alternative splicing
Source: Commun Biol. 2023 Oct 16;6:1046. doi: 10.1038/s42003-023-05409-6 (PMC10579421; doi:10.1038/s42003-023-05409-6)
Supplement: Supplementary file 4 — nr-reporting-summary [file 42003_2023_5409_MOESM4_ESM.pdf]

nature portfolio

Corresponding author(s): Hao Liang, Li Ye, Junjun Jiang

Last updated by author(s): Sep 19, 2023

Reporting Summary

Nature Portfolio wishes to improve the reproducibility of the work that we publish. This form provides structure for consistency and transparency in reporting. For further information on Nature Portfolio policies, see our Editorial Policies and the Editorial Policy Checklist.

Statistics

For all statistical analyses, confirm that the following items are present in the figure legend, table legend, main text, or Methods section.

n/a	Confirmed	
☐	☒	The exact sample size (n) for each experimental group/condition, given as a discrete number and unit of measurement
☐	☒	A statement on whether measurements were taken from distinct samples or whether the same sample was measured repeatedly
☐	☒	The statistical test(s) used AND whether they are one- or two-sided<br>*Only common tests should be described solely by name; describe more complex techniques in the Methods section.*
☐	☒	A description of all covariates tested
☐	☒	A description of any assumptions or corrections, such as tests of normality and adjustment for multiple comparisons
☒	☐	A full description of the statistical parameters including central tendency (e.g. means) or other basic estimates (e.g. regression coefficient) AND variation (e.g. standard deviation) or associated estimates of uncertainty (e.g. confidence intervals)
☐	☒	For null hypothesis testing, the test statistic (e.g. F, t, r) with confidence intervals, effect sizes, degrees of freedom and P value noted<br>*Give P values as exact values whenever suitable.*
☒	☐	For Bayesian analysis, information on the choice of priors and Markov chain Monte Carlo settings
☒	☐	For hierarchical and complex designs, identification of the appropriate level for tests and full reporting of outcomes
☒	☐	Estimates of effect sizes (e.g. Cohen's d, Pearson's r), indicating how they were calculated

Our web collection on statistics for biologists contains articles on many of the points above.

Software and code

Policy information about availability of computer code

Data collection	T. marneffei strain (ATCC18224) was obtained from American Type Culture Collection (ATCC) and used in all infection experiments. THP-1 cells were obtained from American Type Culture Collection (ATCC) and cultured in RPMI 1640 medium (Solarbio, China) containing 10% fetal bovine serum (FBS, Gibco, USA) and 1% penicillin/streptomycin (Solarbio, China). T. marneffei infection of macrophages followed the method described in previous studies. Lentiviral siRNA vector system GV248 (hU6-MCS-Ubiquitin-EGFP-IRES-puromycin) and lentiviral overexpression vector system GV208 (Ubi-MCS-3FLAG-EGFP) were constructed, packaged, and purified by GeneChem (Shanghai, China), and manipulated according to the protocols provided by the manufacturer. Total proteins, nuclear proteins, or cytoplasmic proteins were separated by 10-12% SDS-PAGE (BOSTER, China) and transferred to PVDF membranes (Bio-Rad, USA), and the images were performed using the Odyssey CLX two-color infrared laser imaging system (Odyssey LI-COR, USA). Cell lysates were prepared in cell lysis buffer (for immunoprecipitation, Cell Signaling Technology #9806) or Co-IP buffer (for co-immunoprecipitation, Cell Signaling Technology #87787) supplemented with phosphatase and protease inhibitors. Chromatin immunoprecipitation was carried out using agarose ChIP kit (Thermo Scientific, USA). RIP assay was performed using RIP RNA-Binding Protein Immunoprecipitation kit (Sigma, USA). The library preparation and RNA-seq was performed by GeneChem Co., Ltd (Shanghai, China).
Data analysis	Each experiment was repeated at least 3 times and performed independently. Data were present as the mean ± SD and analyzed using Student's t test or one-way ANOVA analysis. $P < 0.05$ was considered statistically significant. Normality of data was tested using the Shapiro-Wilk test. For RNA-seq analyses, DEGs were calculated in DESeq2 using the Wald test with Benjamini-Hochberg correction to determine FDR. The analyses were performed using SPSS 23.0, Graphpad Prism 8.0, or R studio.

For manuscripts utilizing custom algorithms or software that are central to the research but not yet described in published literature, software must be made available to editors and reviewers. We strongly encourage code deposition in a community repository (e.g. GitHub). See the Nature Portfolio guidelines for submitting code & software for further information.

Data

Policy information about availability of data

All manuscripts must include a data availability statement. This statement should provide the following information, where applicable:

- Accession codes, unique identifiers, or web links for publicly available datasets
- A description of any restrictions on data availability
- For clinical datasets or third party data, please ensure that the statement adheres to our policy

We deposited the raw fastq files in the Sequence Read Archives (SRA) of the National Center for Biotechnology Information (NCBI) under accession number GSE200512, GSE154779 of Bioproject PRJNA824858, PRJNA647412, respectively.

Research involving human participants, their data, or biological material

Policy information about studies with human participants or human data. See also policy information about sex, gender (identity/presentation), and sexual orientation and race, ethnicity and racism.

Reporting on sex and gender	Not applicable
Reporting on race, ethnicity, or other socially relevant groupings	Not applicable
Population characteristics	Not applicable
Recruitment	Not applicable
Ethics oversight	Not applicable

Note that full information on the approval of the study protocol must also be provided in the manuscript.

Field-specific reporting

Please select the one below that is the best fit for your research. If you are not sure, read the appropriate sections before making your selection.

☒ Life sciences ☐ Behavioural & social sciences ☐ Ecological, evolutionary & environmental sciences

For a reference copy of the document with all sections, see nature.com/documents/nr-reporting-summary-flat.pdf

Life sciences study design

All studies must disclose on these points even when the disclosure is negative.

Sample size	Not applicable
Data exclusions	Not applicable
Replication	Not applicable
Randomization	Not applicable
Blinding	Not applicable

Reporting for specific materials, systems and methods

We require information from authors about some types of materials, experimental systems and methods used in many studies. Here, indicate whether each material, system or method listed is relevant to your study. If you are not sure if a list item applies to your research, read the appropriate section before selecting a response.

Materials & experimental systems

n/a	Involved in the study
☐	☒ Antibodies
☐	☒ Eukaryotic cell lines
☒	☐ Palaeontology and archaeology
☒	☐ Animals and other organisms
☒	☐ Clinical data
☒	☐ Dual use research of concern
☒	☐ Plants

Methods

n/a	Involved in the study
☒	☐ ChIP-seq
☒	☐ Flow cytometry
☒	☐ MRI-based neuroimaging

Antibodies

Antibodies used

Human recombinant IFN-γ and GM-CSF antibodies were purchased from Sinobio Biotechnology, and the LPS (from E. coli) was from Sigma-Aldrich. The IRDye 680RD donkey anti-mouse and IRDye 800CW donkey anti-rabbit antibodies were purchased from LI-COR Biosciences. Anti-rabbit IgG (H+L), F(ab')2 fragment (Alexa Fluor® 647 Conjugate), anti-FLAG (D6W5B), anti-PCNA (D3H8P), anti-β-actin (8H10D10), anti-normal Rabbit IgG, anti-HDAC3 (D2O1K), anti-TBL1XR1/TBLR1 (D4J9C), anti-JunB (C37F9), anti-phospho-JunB (Thr102/Thr104) (D3C6), anti-JunD (D17G2), anti-c-Jun (60A8), anti-phospho-c-Jun (Ser73), anti-acetyl-histone H3 (Lys27) (D5E4), and anti-GAPDH (D16H11) antibodies were purchased from Cell Signaling Technology. Anti-NCOR2 antibody was obtained from Novus Biologicals. Anti-TUT1 (PA5-50151) antibody was obtained from Invitrogen.

Validation

All primary antibodies have shown efficacy and the validation results are satisfactory.

Eukaryotic cell lines

Policy information about cell lines and Sex and Gender in Research

Cell line source(s)

THP-1 cells were obtained from American Type Culture Collection (ATCC) and cultured in RPMI 1640 medium (Solarbio, China) containing 10% fetal bovine serum (FBS, Gibco, USA) and 1% penicillin/streptomycin (Solarbio, China). The cells were allowed to differentiate in macrophages with the treatment of 100 ng/mL phorbol myristate acetate (PMA, Sigma, USA) for 48 h.

Authentication

Verification of THP-1 cells using Short Tandem Repeat (STR) technology.

Mycoplasma contamination

All cell lines tested negativefor mycoplasma contamination

Commonly misidentified lines
(See ICLAC register)

Not applicable
